# Synthesis and Biological Evaluation of 3-(Pyridine-3-yl)-2-Oxazolidinone Derivatives as Antibacterial Agents

**DOI:** 10.3389/fchem.2022.949813

**Published:** 2022-07-18

**Authors:** Bo Jin, Tong Wang, Jia-yi Chen, Xiao-qing Liu, Yi-xin Zhang, Xiu-ying Zhang, Zun-lai Sheng, Hong-Liang Yang

**Affiliations:** ^1^ Department of Veterinary Medicine, Northeast Agricultural University, Harbin, China; ^2^ Heilongjiang Key Laboratory for Animal Disease Control and Pharmaceutical Development, Harbin, China

**Keywords:** 3-(pyridine-3-yl)-2-oxazolidinone, antibacterial activity, molecular docking, biofilm formation inhibitory activity, drug resistance development

## Abstract

In this research, a series of 3-(pyridine-3-yl)-2-oxazolidinone derivatives was designed, synthesized, and evaluated for *in vitro* antibacterial activity, which included bacteriostatic, morphological, kinetic studies, and molecular docking. The results demonstrated that compounds **21b**, **21d**, **21e** and **21f** exhibited strong antibacterial activity similar to that of linezolid toward five Gram-positive bacteria. After observing the effect of the drug on the morphology and growth dynamics of the bacteria, the possible modes of action were predicted by molecular docking. Furthermore, the antibiofilm activity and the potential drug resistance assay was proceeded. These compounds exhibited universal antibiofilm activity and compound **21d** showed significant concentration-dependent inhibition of biofilm formation. Compound **21d** also showed a stable effect on *S. pneumoniae* (ATCC 49619) with less drug resistance growth for 15 days, which is much longer than that of linezolid. Overall, these results can be used to guide further exploration of novel antimicrobial agents.

## Introduction

The misuse and overuse of antibiotics in medical treatments and animal husbandry leads to the development of bacterial resistance, which greatly reduces the efficacy of antibiotics (Zhang et al., 2018). Bacteria have become resistant to almost all existing antibiotics by changing binding targets, producing resistant enzymes or via other resistance mechanisms (R. Laxminarayan et al., 2013; M. Madhu Sekhar et al., 2018). Nowadays, high doses of antibiotics are always needed to deal with common bacterial infections. Infections caused by resistant bacteria are spreading rapidly all over the world, which has become a major threat to global public health. A consequence is that common infections are likely to be incurable in the future. What is more serious is that no new anti-bacterial agents have been found (M.S. Butler et al., 2017). Therefore, it is of great significance to develop novel antimicrobials, as well as modify the developed drugs to overcome drug resistance (Yin et al., 2014; Kirk et al., 2018).

Linezolid, as the first oxazolidinone antibacterial agent, inhibits the biosynthesis of bacterial protein by acting on the 50S subunit of ribosome (Ippolito et al., 2008), and has extraordinary efficacy in infection caused by Gram-positive bacteria such as methicillin-resistant *Staphylococcus aureus* (MRSA), penicillin-resistant *Streptococcus pneumoniae* (PRSP), and vancomycin-resistant *Enterococcus* (VRE) (Brickner et al., 2008). However, due to the long-term use of linezolid, a variety of linezolid-resistant bacteria such as *Staphylococcus aureus*, *Enterococcus faecium* and many others have developed (Tsiodras et al., 2001; Auckland et al., 2002; Morales et al., 2010). Over the past 2 decades, medicinal chemists have carried out lots of structural modification studies on linezolid, in order to develop new antibiotics with better activities and fewer side effects (Deshmukh and Jain, 2017; Siddiqui et al., 2018). Duffy reported that the A ring, the N atom on the C-5 side chain and the aromatic B ring of linezolid contribute greatly to drug binding (Duffy et al., 2008). There have been many studies on the adjustments of the C ring, A ring and C-5 side chain of linezolid (Wu et al., 2019; Wu et al., 2018; Palumbo Piccionello et al., 2012; Bai et al., 2018; Cruz et al., 2021; De Rosa et al., 2013; Ferrazzano et al., 2016; Fortuna et al., 2013; Gadekar et al., 2016; Naresh et al., 2014), but less on the B ring (Cui et al., 2005; Sbardella et al., 2004). Why is this? Will the effect be improved if the B ring is replaced with other heteroaromatic rings?

**FIGURE 1:**
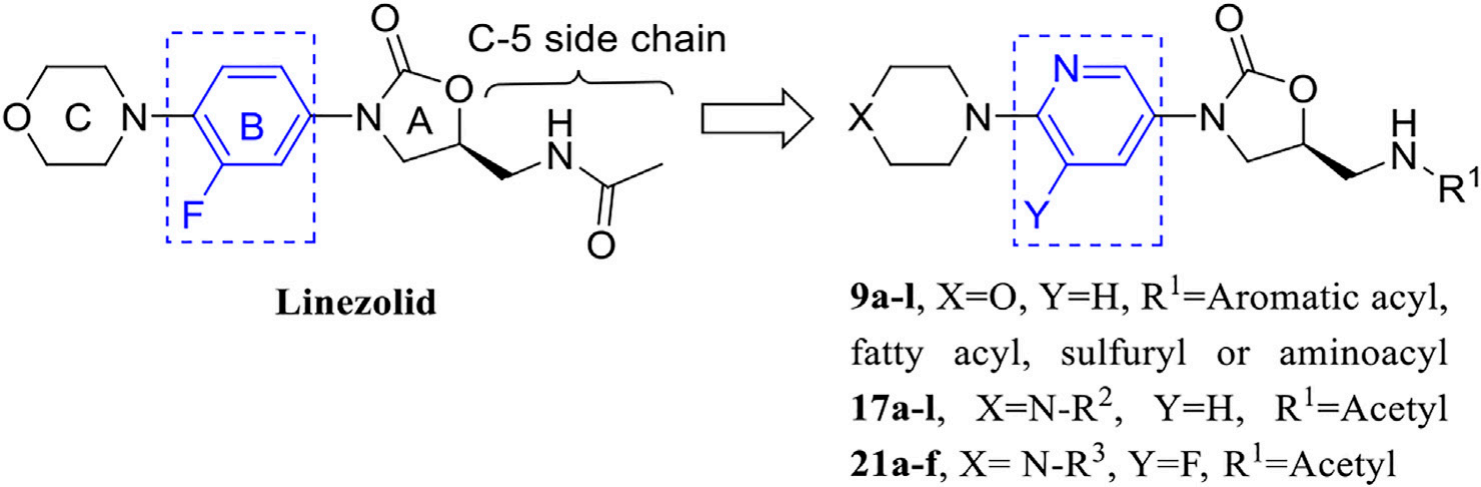
Optimization strategy of 3-(pyridine-3-yl)-2-oxazolidinone derivatives.

In order to explore this problem, we report a strategy to modify the B ring of linezolid (Figure 1). On the one hand, pyridine heterocycle, as a bioisostere of the benzene ring, is widely used in various small molecule drugs due to its excellent pharmacokinetic property and stability in human and the ease of N-atom bonding with nucleotide through hydrogen bonding (Vara Prasad et al., 2006; Perry et al., 2001; Cappuccino and Sherman, 2000). On the other hand, we have previously synthesized many pyridine heterocycle derivatives and they have potential activity (Jin et al., 2018; Yang et al., 2019; Jin et al., 2022). That’s why we report these syntheses and antibacterial activities of 3-(pyridine-3-yl)-2-oxazolidinone derivatives. Their antibacterial activity, structure-activity relationships, time-growth kinetics, biofilm formation inhibitory activity, potential drug resistance and the preliminary mechanisms of the action are explored.

## Results and Discussion

### Chemistry

**SCHEME 1:**
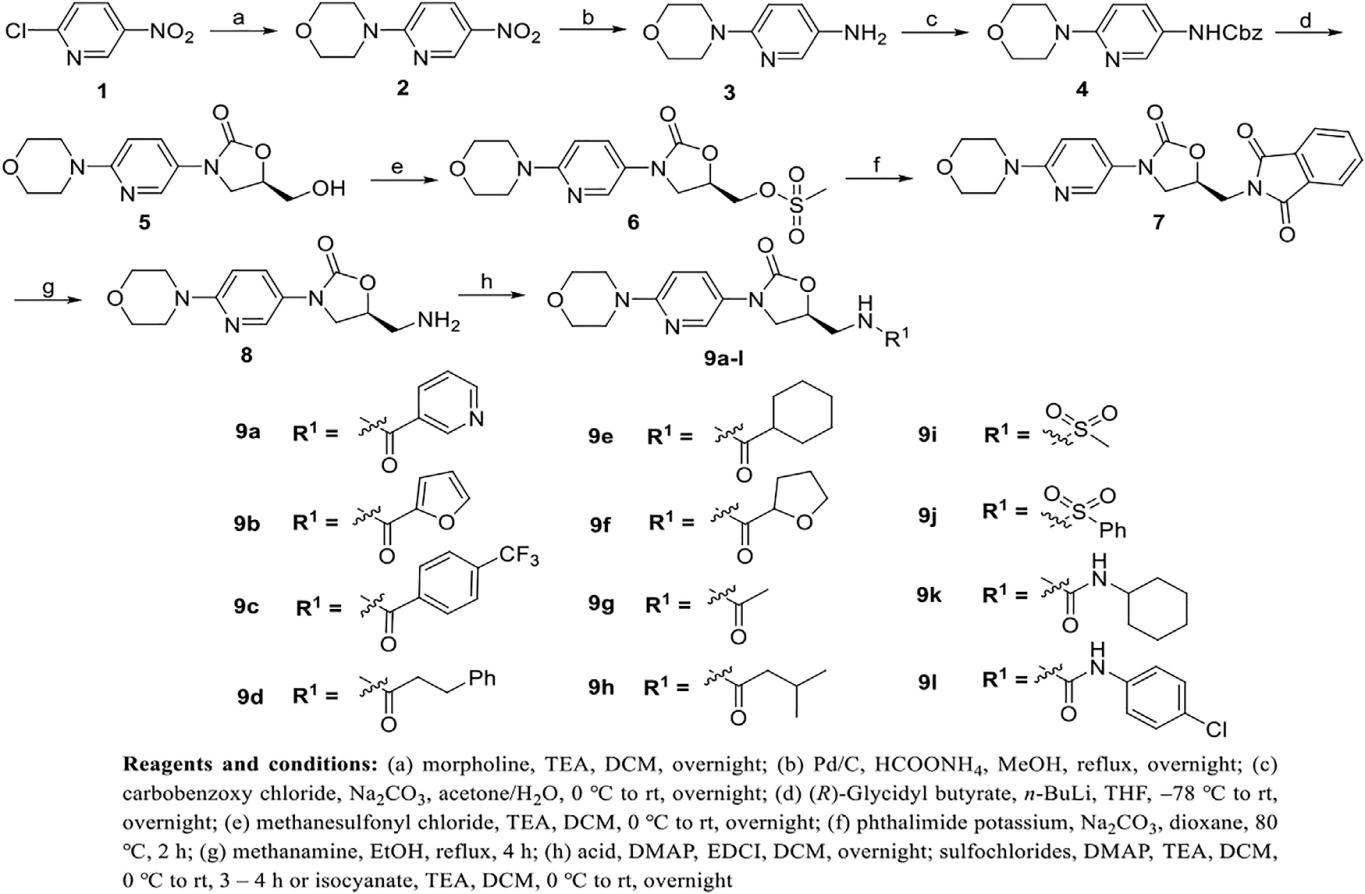
Synthesis of target compounds **9a-l**.

The long linear synthetic pathway employed to prepare derivatives **9a-l** is illustrated in Scheme 1. Commercially available 2-chloro-5-nitropyridine (**1**) was reacted with morpholine at room temperature to give intermediate **2**. Carbamate intermediate **4** was obtained with carbobenzoxy chloride at 0 °C after hydrogenation of nitro by Pd-C/HCOONH_4_ in methanol at reflux. Oxazolidinone intermediate **5** was accomplished by use of *n*-butyl lithium and (*R*)-glycidyl butyrate in dry THF at -78 °C. Then the methane sulfonyl and the Pht group were connected and removed to prepare the key intermediate **8**. Finally, various acids, sulfonyl chlorides and isocyanates were introduced to yield compounds **9a-l**.

**SCHEME 2:**
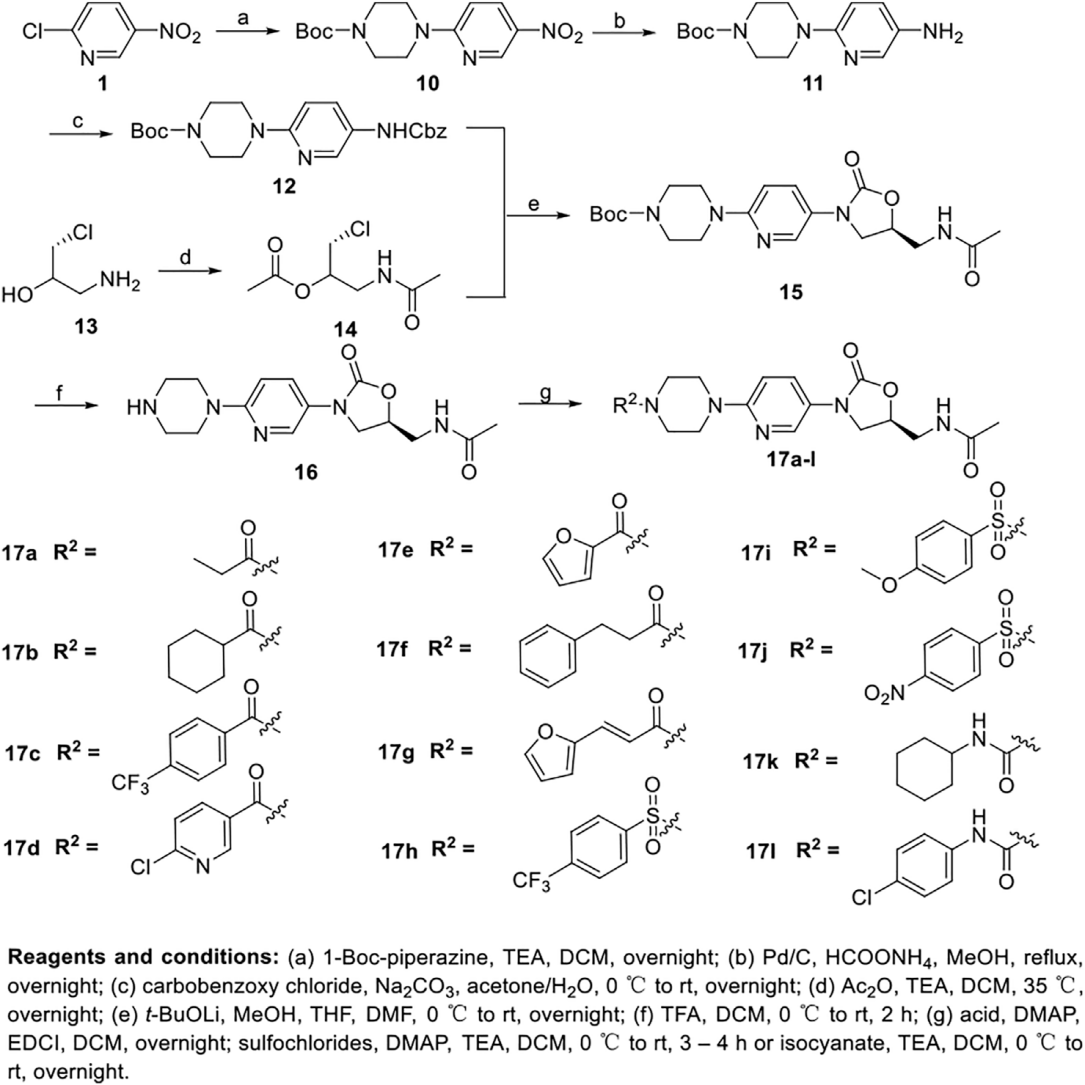
Synthesis of target compounds **17a-l**.

The synthetic route to prepare derivatives **17a-l** is presented in Scheme 2. 2-chloro-5-nitropyridine **1** reacted with 1-Boc-piperazine to afford **10**. Intermediate **12**, obtained in the same way as depicted above, was reacted with diacetylation intermediate **14** to give oxazolidinone **15**. Subsequently, the Boc protection group was removed and final products **17a-l** were prepared.

**SCHEME 3:**
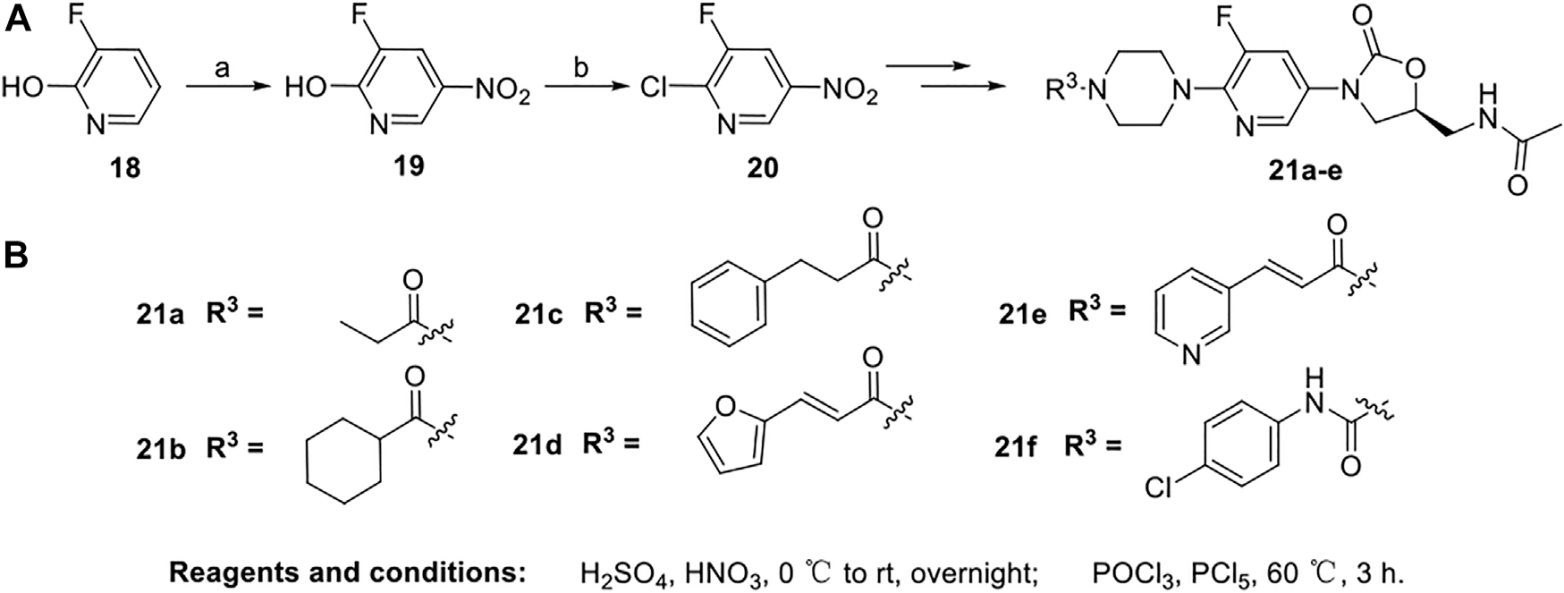
Synthesis of target compounds **21a-f**.

The syntheses of derivatives **21a-f** are outlined in Scheme 3. Commercially available 3-fluoro-2-hydroxypyridine **18** was treated with concentrated sulfuric acid and concentrated nitric acid to give **19** at 0 °C. Compound **20** was obtained in POCl_3_ in the presence of PCl_5_ at 60 °C. Derivatives 21a-f were synthesized in the same manner as products **17a-l**.

### Antimicrobial Activity

As linezolid is more effective on Gram-positive bacteria than Gram-negative bacteria (Vara Prasad et al., 2006), here the target compounds were examined considering five Gram-positive bacteria *S. aureus* (ATCC25923), *S. pneumonia* (ATCC49619), *E. faecalis* (ATCC29212), *B. subtilis* (ATCC6633), *S. xylosus* (ATCC35924) by the standard serial dilution method. Linezolid was used as the standard drug and the minimum inhibitory concentrations (MICs in μg/mL) of all the synthesized compounds as well as the positive control are depicted in Table 1.

**TABLE 1:** MICs (μg/ml) of compounds **9a-l**, **17a-l** and **21a-f** against five Gram-positive bacteria.

Comp	Sa^a^	Sp^b^	Ef^c^	Bs^d^	Sx^e^
**9a**	64	>256	>256	32	>256
**9b**	64	128	128	>256	>256
**9c**	64	>256	>256	>256	>256
**9d**	32	>256	>256	>256	>256
**9e**	64	>256	>256	>256	>256
**9f**	64	>256	>256	>256	>256
**9g**	32	64	64	32	256
**9h**	64	>256	>256	64	>256
**9i**	64	>256	>256	>256	>256
**9j**	64	>256	>256	>256	>256
**9k**	64	>256	>256	>256	>256
**9L**	64	>256	>256	>256	>256
**17a**	32	64	128	128	128
**17b**	32	32	128	128	128
**17c**	32	128	128	256	128
**17d**	128	32	128	256	128
**17e**	64	64	128	128	128
**17f**	32	32	128	128	128
**17g**	16	8	128	128	128
**17h**	256	256	128	128	128
**17i**	>256	>256	>256	128	>256
**17j**	>256	256	>256	>256	>256
**17k**	128	64	128	128	128
**17L**	64	16	128	128	128
**21a**	8	16	32	128	8
**21b**	4	8	64	4	8
**21c**	16	64	32	32	32
**21d**	4	4	8	8	8
**21e**	4	8	32	64	8
**21f**	4	8	16	4	8
**Linezolid**	2	2	2	2	2
**Chlortetracycline**	2	4	8	8	8
**Vancomycin**	1	2	2	1	1
**Methicillin**	2	1	2	2	4

a Sa, *Staphylococcus aureus* (ATCC25923).

b Sp, *Streptococcus pneumoniae* (ATCC49619).

c Ef, *Enterococcus faecalis* (ATCC29212).

d Bs, *Bacillus subtilis* (ATCC6633).

e Sx, *Staphylococcus xylosus* (ATCC35924). MICs, were determined in three independent experiments.

Firstly, while replacing the B ring of linezolid with pyridine, various pharmaceutical molecular fragments on the 5-amino side chain of the A ring (NHR^1^) were examined. The results showed most of these compounds had moderate antibacterial activities against *S. aureus* (MIC = 32–64 μg/ml) and poor effects on the four other bacteria, which indicated that *S. aureus* was sensitive to these compounds. From the results, it was seen that only **9g** had universal antibacterial activity against the tested bacteria, which suggested that only the small acetyl substituent group could be tolerated at the 5-amino side chain of the A ring, and other adipose or aromatic substituent groups were not suitable. Therefore, it was speculated that there might only be a narrow binding pocket at the 5-position side chain of the A ring, which can accommodate no other larger groups.

Although it has a broad antibacterial spectrum, compound **9g** showed only moderate antibacterial activity (MIC = 32–256 μg/ml). We suspected the B ring and C ring in this structure may not play a full role in the binding with the target. So, structure modification of the C ring was carried out and derivatives **17a-l** were synthesized and tested accordingly. It was found that these compounds displayed better antibacterial activities than **9a-l**. Most of the compounds showed certain inhibitory activities against *E. faecalis, B. subtilis* and *S. xylosus*, and more significant inhibitory effects against *S. aureus* and *S. pneumoniae*. The biological activities of aliphatic amide derivatives **17a**, **17b** were slightly better than that of aromatic amide derivatives **17c-e**, while sulfonamide derivatives **17h-j** had the worst effect. Compound **17g** showed substantial antibacterial activity, with MICs in the low to mid-single digit microgram range. Aromatic urea derivative **17L** had slightly better activity than aliphatic urea derivative **17k**. Based on the results above, it was considered that the substituent groups on piperazine have remarkable influence on the efficacy. Presumably, there may be a large binding pocket in the binding region of the piperazine ring, which can accommodate different groups. The activity of the compound **17g** may be related to the double bond in the amide side chain.

After the side chain exploration of the A ring and C ring, the fluorine atom was introduced back into the B ring to obtain compounds **21a-f**. The test results showed that these compounds had significantly increased antibacterial activity against the five bacteria, among which compounds **21b**, **21d** and **21f** showed significant inhibitory effects against a variety of bacteria. The introduction of the fluorine atom has a significant effect on the improvement of antibacterial activity, and there are many reports showing that the fluorine atom can promote the bioactivity of drugs (Kirk, 2006). Here we speculated that the electron cloud density of the pyridine ring was reduced due to the introduction of fluorine, which may improve the drug binding to the target, or make the drug molecules penetrate biofilm more easily and enhance drug distribution in the target.

### Morphological Observation

To observe the morphological changes of bacteria after being treated by promising compounds **21b**, **21d** and **21f** more intuitively, *S. pneumoniae* and *S. aureus* were treated at a concentration of 1/2 × MIC and observed by Scanning electron microscopy (SEM).

**FIGURE 2:**
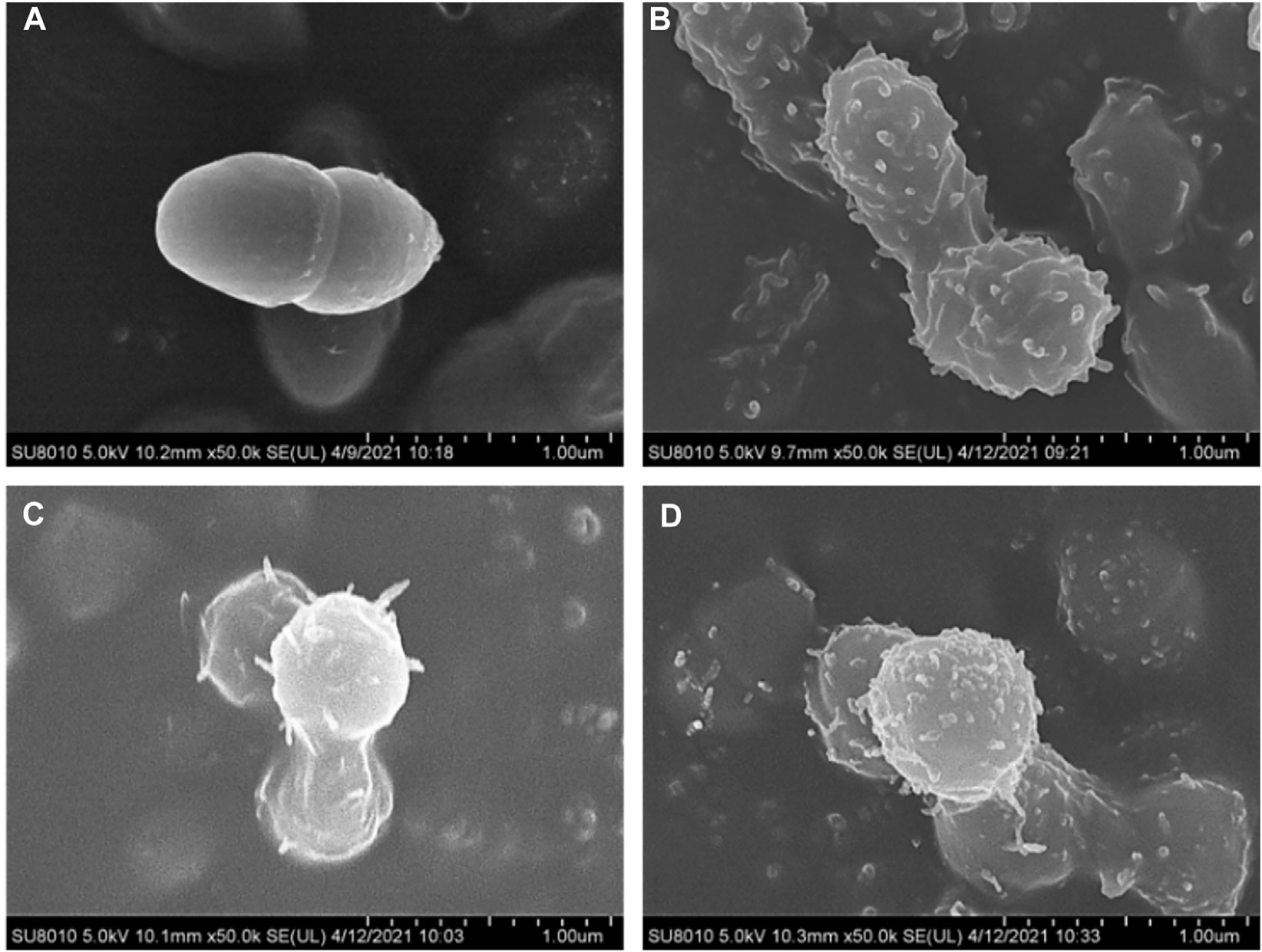
SEM observation of *S. pneumoniae* ATCC 49619, before **(A)** and after treatment with 1/2 × MIC concentration of compounds **21b (B)**, **21d (C)** and **21f (D)**.

As shown in Figure 2A, the untreated *S. pneumoniae* revealed a coccus, with a clear outline, a complete membrane and a smooth surface. After contacting with active compounds, the form of *S. pneumoniae* was significantly changed as shown in Figures 2B–D. The injuries to the cell walls could be observed precisely, and the cell permeability was disordered. As a result, the shape of the bacteria was no longer round and became twisted and irregularly folded. Furthermore, there was visible damage on the surface of the cells, such as bushy hairlike deformations and adhesion between multiple cells.

**FIGURE 3:**
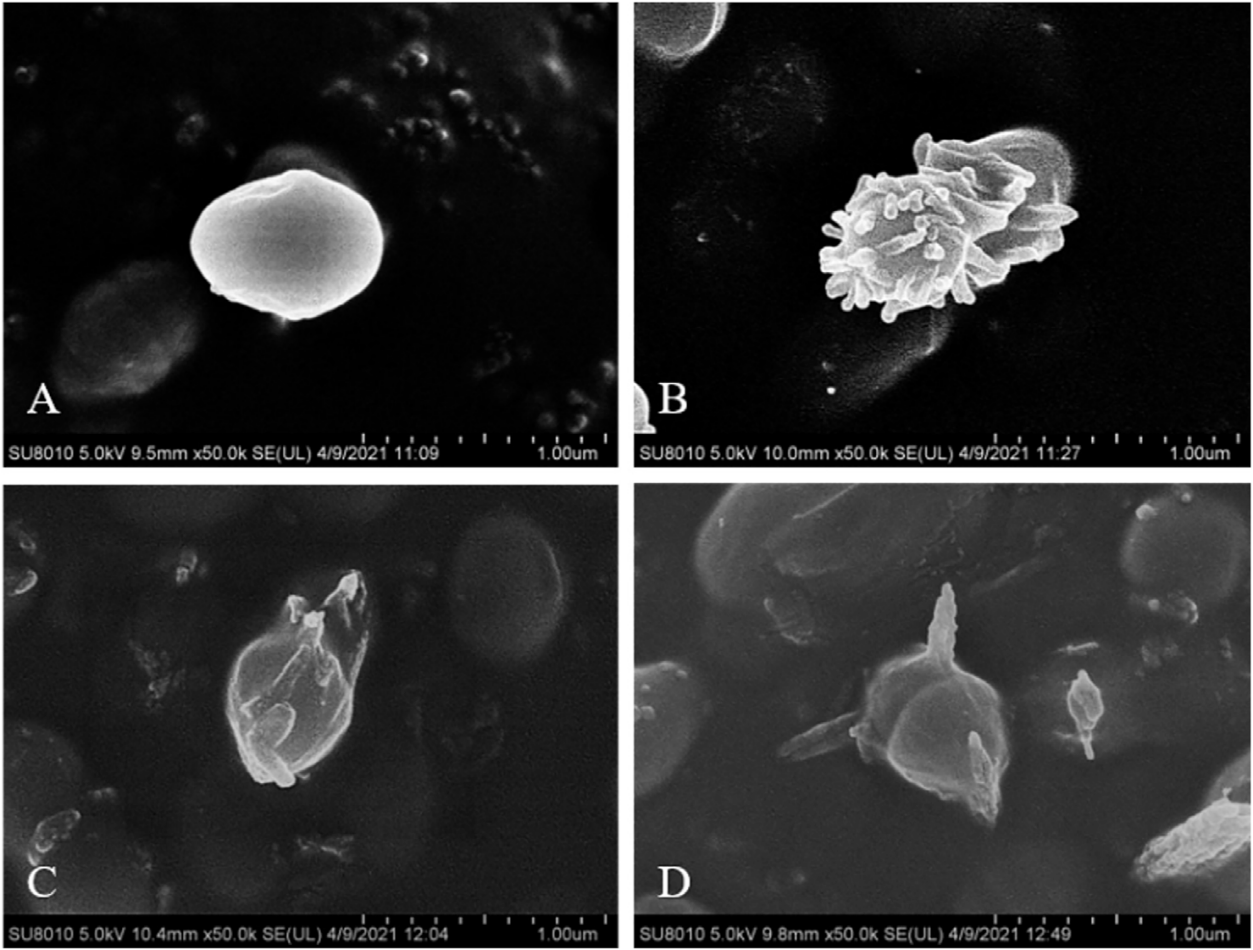
SEM observation of *S. aureus* ATCC 25923, before **(A)** and after treatment with 1/2 × MIC concentration of compounds **21b (B)**, **21d (C)** and **21f (D)**.

The untreated *S. aureus* was observed by SEM as a coccus with clear edges, and smooth and full surfaces, as shown in Figure 3A. The bacteria treated with these three compounds were all seriously deformed, and even the surfaces showed dense villous changes after being treated with **21b**. The cell walls were digested, with a spiked deformation on the surface and the bio-membrane showed a tendency of melting after being treated with compounds **21d** and **21f**.

### Time-Growth Kinetics of the Bacteriostatic Activity

**FIGURE 4:**
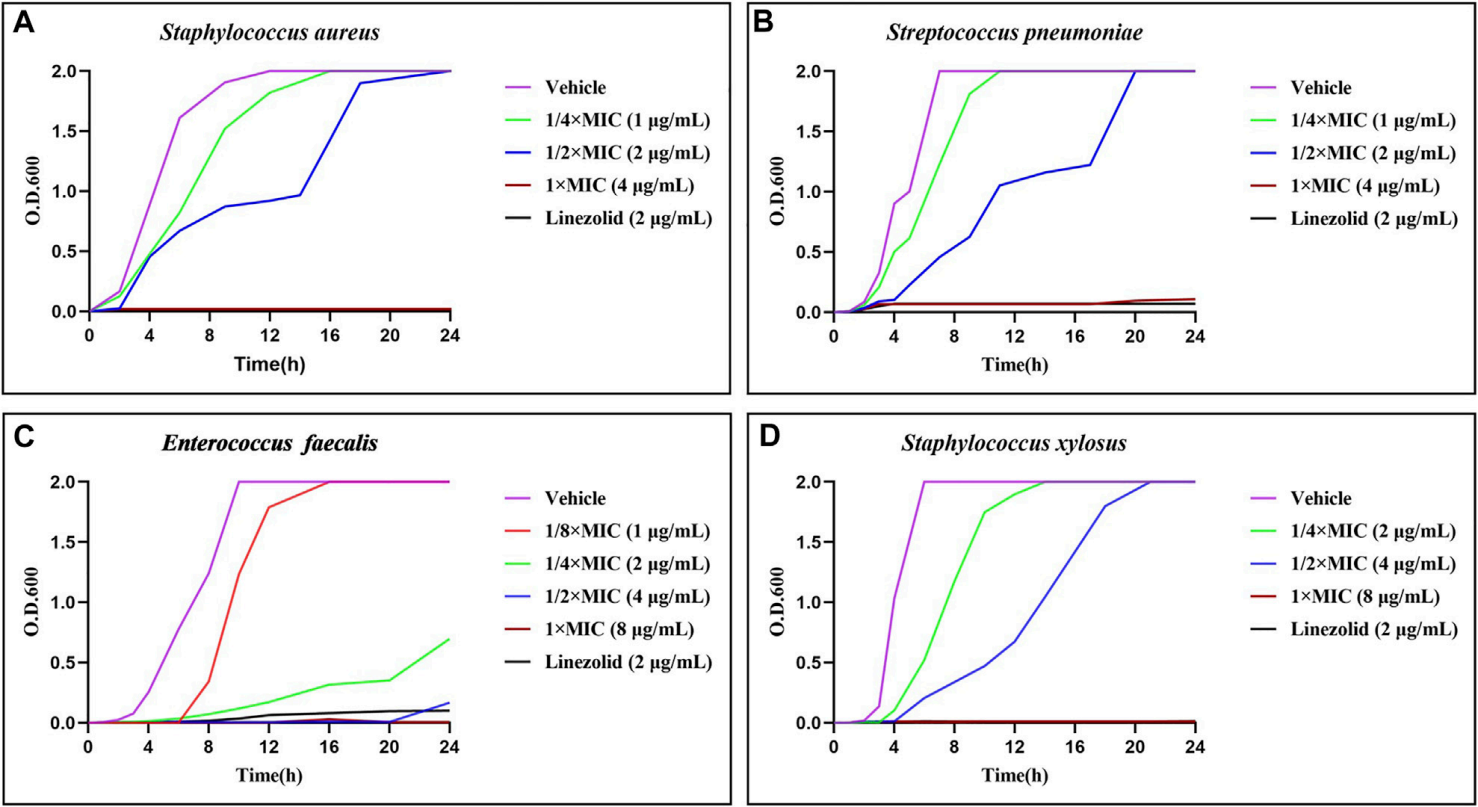
Time-growth kinetics for the compound **21d** and linezolid (1/2 × MIC) against *S. aureus* **(A)**, *S. pneumoniae* **(B)**, *E. faecalis* **(C)** and *S. xylosus* **(D)** within 24 h.

The time-growth kinetics approach for Gram-positive bacteria strains was used to investigate the antimicrobial kinetics of the promising compound (Theophel et al., 2014; Koh et al., 2013). As shown in Figure 4, except *E. faecalis*, the other three bacteria increased sharply in the counts of O.D.600 without treatment or in the presences of 1/4 × MIC of **21d**. With the increase of concentration, the bacteriostatic efficacy was enhanced until it was similar to that of linezolid. The killing efficacy on *E. faecalis* was shown on the curve as a longer lag period and lagged log period, especially at 1/4 × MIC. These results indicated that **21d** could be used as an excellent concentration-dependent broad-spectrum antimicrobial agent.

### Molecular Docking Study

To explore the initial binding modes of these derivatives, compound **17g** was selected to dock with the crystal structure of the 50S ribosome subunit of *Haloarcula marismortui* by Auto Dock tool.

**FIGURE 5:**
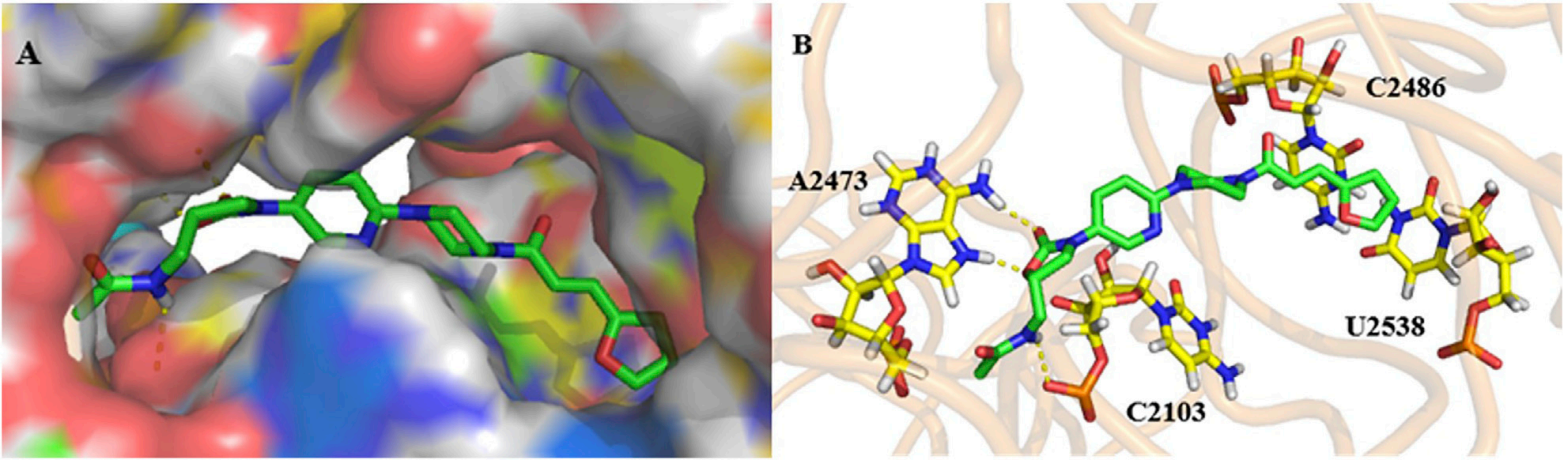
Docking study: compound **17g** (green) docked with ribosomal peptidyl transferase center (PTC) of 50S ribosomal subunit from *Haloarcula marismortui* (PDB ID: 3CPW). **(A)** compound 17g in an active pocket; **(B)** compound 17g docking with a nucleotide residue.

As shown in Figure 5A, compound **17g** is located in a long and narrow pocket in the ribosomal peptidyl transferase center (PTC), with a fully extended state. In addition, there is only a shallow hydrophobic pocket at the 5-position amide side chain of the A ring, where large substituent groups cannot be accommodated, this may be the reason why **9g** is more effective than the other compounds. Moreover, a long hydrophobic pocket is observed at the chain side of piperazine, which could accommodate different substituent groups. Owing to the molecular length, series **17** can be more closely combined with the pocket and have better efficacy than series **9**.

The interaction mode of compound **17g** with the target was predicted, as shown in Figure 5B. It can be seen that two hydrogen bonds are formed between two hydrogen atoms of A2473 and oxygen atoms on the A ring and the carbonyl group. The third one was predicted between the hydrogen atom on the chain amide at the 5-position of the A ring and the oxygen atom of C2103. Furthermore, it can be speculated that both the double bond and furan ring on the amide side chain have conjugation with pyrimidine of C2486 and U2538, which may be the reason why compound **17g** has better activity.

**FIGURE 6:**
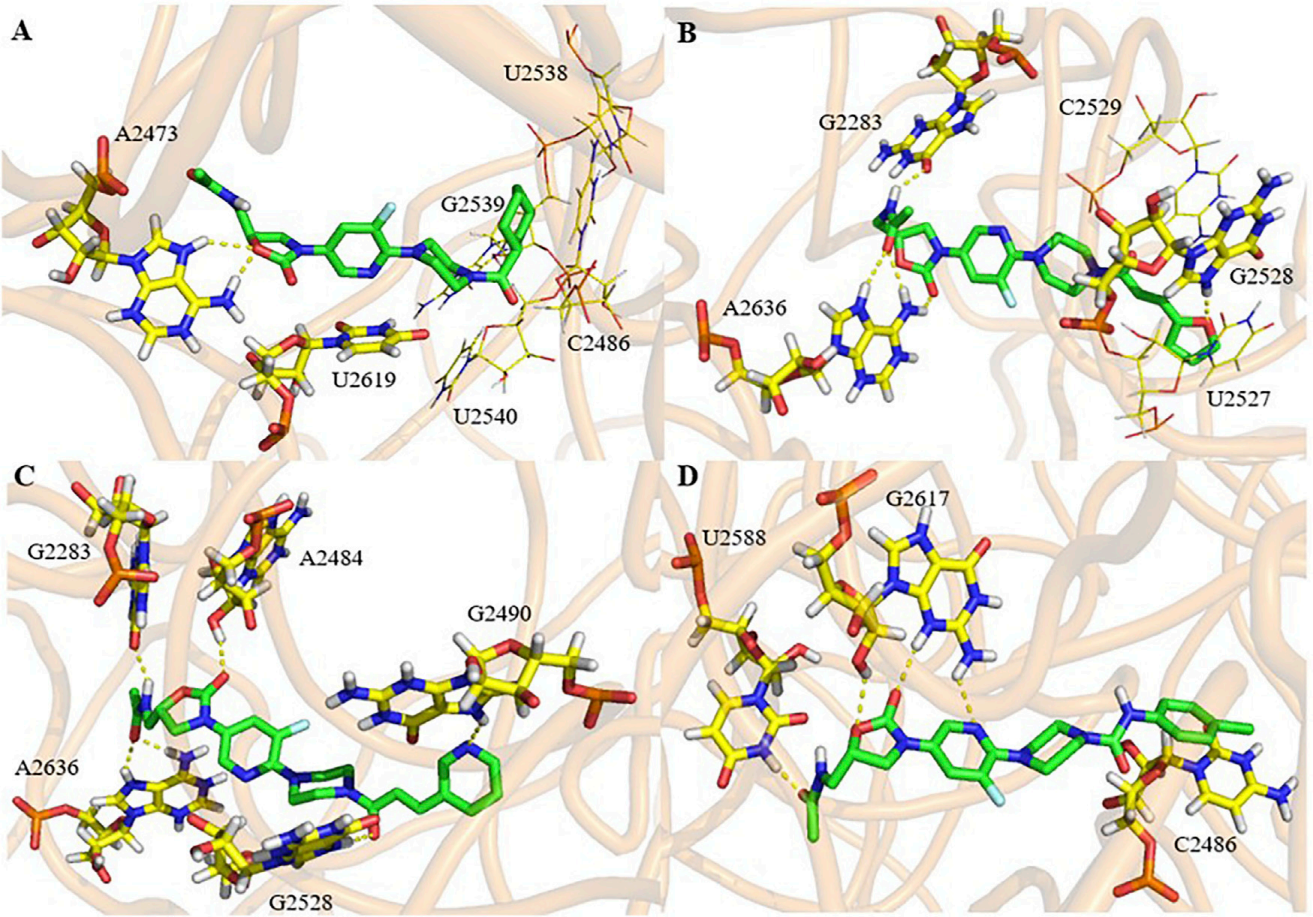
Docking study of compounds **21b (A)**, **21d (B)**, **21e (C)** and **21f (D)** with ribosomal peptidyl transferase center (PTC) of 50S ribosomal subunit from *Haloarcula marismortui* (PDB ID: 3CPW).

The binding mode of the active compounds **21b**, **21c**, **21d** and **21f** to the target is also predicted in Figure 6. It can be seen that all the molecules are in a relatively extended state and most of the hydrogen bonding takes place on the A ring and the 5-amide side chain with purine or pyrimidine residues. The furan and pyridine ring of compounds **21b** and **21d** also participate in the formation of hydrogen bonds. The cyclohexyl group of **21b** has hydrophobic interaction with the residues of U2538, G2539, U2540 and C2846 (shown in lines). The furan ring and double bond on the side chain of piperazine amide in **21d** conjugates with pyrimidine of U2527 and C2529 (shown in lines), and a similar interaction occurs between the benzene ring of **21f** and pyrimidine of C2486.

The predicted binding energy, dissociation constants and the bases involved in the formation of hydrogen bonds are listed in Table 2. It can be seen that all the selected compounds display good binding energy and H-bond interactions with the target. Among them, compounds **21b** and **21f** exhibited higher binding energy and better dissociation constants, which indicates that they will have good target affinity. It can be concluded that the fluorine atom in these molecules is not involved in hydrogen bonding or other interaction. Therefore, it is possible to enhance the drug efficacy by improving the permeability of the cell membrane or drug distribution.

**TABLE 2:** Molecular docking binding affinities to antibacterial targets.

	Binding Energy, △G (kcal/Mol)	Dissociation Constant (kI) (μM)	Interacting Residues
**17g**	–6.97	7.75	A2473, C2103
**21b**	–7.36	4.00	A2473
**21d**	–6.27	25.28	A2636, G2283, G2528
**21e**	–6.13	32.04	A2636, G2283, A2484
**21f**	–7.46	3.40	U2588, G2617

### Antibiofilm Activity

Biofilm, a protective membrane formed by the aggregation of metabolites, is known as the main reason for the failure of antibacterial therapy (Kim and Lee, 2016; Parrino et al., 2019; Lebeaux et al., 2014). Here the minimum biofilm inhibitory concentrations (MBICs) of **21a-f** were evaluated to quantify the inhibitory effect of different compounds on biofilm formation (Anbarasi et al., 2020). As shown in Table 3, these compounds exhibited broad-spectrum anti-biofilm activity. Among them compound **21d** showed the best effect on *S. pneumoniae* (MBIC = 0.5 μg/ml). The MBICs value of many compounds were only 1/4 or even 1/32 of their MIC value, indicating that at this concentration, they had little effect on bacterial growth, but strong biological inhibition.

**TABLE 3:** MBICs (μg/ml) of compounds **21a-f** against five Gram-positive bacteria.

Comp	Sa ^a^	Sp ^b^	Ef ^c^	Bs ^d^	Sx ^e^
**21a**	8	8	2	8	8
**21b**	4	2	2	4	8
**21c**	4	8	2	8	8
**21d**	4	0.5	1	8	8
**21e**	2	1	1	4	4
**21f**	2	4	1	4	4

a Sa, *Staphylococcus aureus* (ATCC25923).

b Sp, *Streptococcus pneumoniae* (ATCC49619).

c Ef, *Enterococcus faecalis* (ATCC29212).

d Bs, *Bacillus subtilis* (ATCC6633).

e Sx, *Staphylococcus xylosus* (ATCC35924). MICs, were determined in three independent experiments.

**FIGURE 7:**
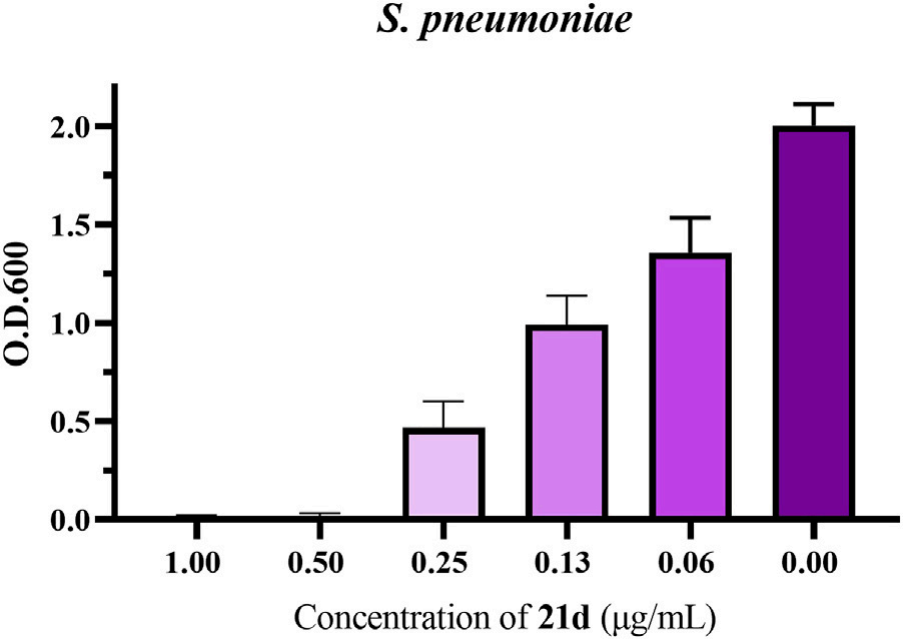
The influence of *S. pneumoniae* biofilm formation treated with **21d**. Biofilm formation (O.D.600) of *S. pneumoniae* (ATCC 49619) in 96-well plates was quantified in the presence of **21d** after 20 h. The error bar represents the standard error of the average calculated using data from at least three independent experiments.

In order to determinate the anti-biofilm activity at different concentrations, the promising compound **21d** was selected to measure its effect on *S. pneumoniae* biofilm formation using crystal violet assay (Jiang et al., 2020). By analyzing the absorbance of crystal violet on each biofilm, the inhibition effect of **21d** and the control (1% DMSO) on biofilm formation was compared. As clearly seen in Figure 7, **21d** significantly reduced the growth of biofilms in a dose-dependent manner. Biofilm formation decreased as the concentration of the drug increased, until the concentration reached MBIC and biofilm formation was almost nonexistent.

### Evaluation of Potential Resistance Development

**FIGURE 8:**
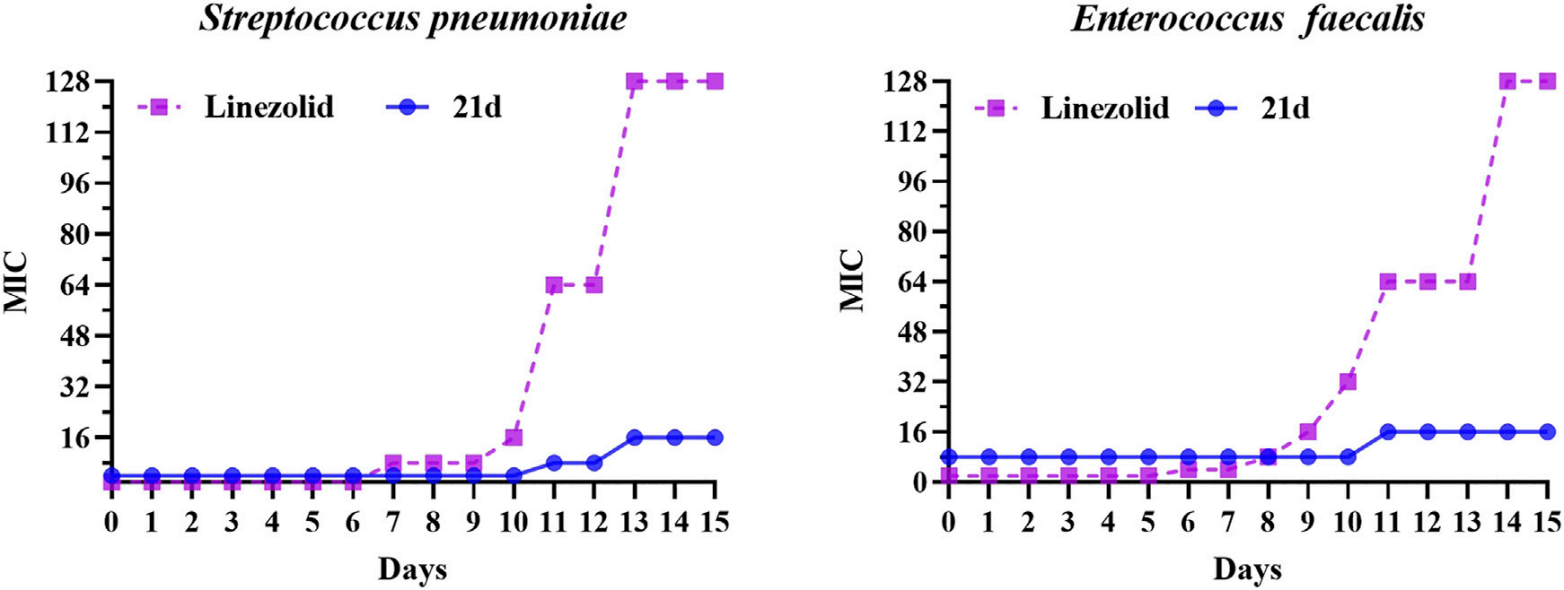
The MIC variability of **21d** and linezolid against *S. pneumoniae* (ATCC 49619) and *Enterococcus faecalis* (ATCC 29212) during 15 days of the resistance development assay, with linezolid as a comparison.

Based on the excellent anti-biofilm activity of **21d**, it was necessary to assess the potential development of resistance. The drug resistance tendency of *S. pneumoniae* (ATCC 49619) and *Enterococcus faecalis* (ATCC 29212) were determined by multi-generation resistance selection assay (Chin et al., 2018; Vikesland et al., 2019; Li et al., 2018; Kieron et al., 2013; Wu et al., 2020). The strain was sub-cultured at subinhibitory concentration (1/2 × MIC) of the novel compound and linezolid for 15 days. As shown in Figure 8, the MIC value of the linezolid group began to increase on the 7^th^ or 8^th^ day, forming drug resistance on the 11th day (MIC ≥64 μg/ml), and increased 64-fold to 128 μg/ml on the 13th day. In contrast, the MIC value of the **21d** group didn’t increase until the 11th day, and reached no higher than 4-fold compared with the initial MIC. These results demonstrated that **21d** could effectively reduce the generation of induced bacterial resistance.

## Conclusion

In summary, a series of novel substituted 3-(pyridine-3-yl)-2-oxazolidinone derivatives were synthesized for evaluation of their antibacterial activity against five Gram-positive bacteria *in vitro*. Among them, compounds **21b**, **21d**, **21e** and **21f** exhibited interesting antibacterial activity. They could destroy cell shape and the inhibitory effect was concentration dependent. The binding modes of these compounds were also predicted by molecular docking. Further drug-resistance studies demonstrated that these compounds had a general formation inhibitory effect on biofilms and **21d** effectively reduced the generation of bacterial resistance. Although the MICs of these compounds were slightly weaker than that of linezolid, considering linezolid has been used in clinical settings for a long time and the problem of bacterial resistance is ubiquitous, the above compounds are expected to be alternatives. Further efforts in structural optimization and activity screening are in progress.

## Experimental

### Chemistry

Unless otherwise noted, all the chemicals and solvents were analytical reagents and used directly without further purification. Solvents were dried according to standard procedures. The melting points were measured by X-4 electrothermal digital melting point apparatus and uncorrected. ^1^H NMR spectra (400 MHz) were recorded on a Bruker Advance spectrometer with tetramethyl silane (TMS) as internal standard and CDCl_3_ or DMSO-*d*_*6*_ as the solvent. ^13^C NMR spectra (75 MHz) were determined with complete proton decoupling.

### Synthesis of 4-(5-Nitropyridin-2-yl) Morpholine (2)

To a well-stirred solution of 2-chloro-5-nitropyridine **1** (474 mg, 3 mmol) and morpholine (0.32 ml, 3.6 mmol) in CH_2_Cl_2_ (10 ml), triethylamine (0.5 ml, 3.6 mmol) was added, then the mixture was stirred at room temperature overnight. The mixture was extracted with CH_2_Cl_2_ (10 ml × 3). The organic phase was washed with brine and concentrated in vacuo to yield **2** as a yellow solid: yield 93%; mp: 144.2–144.9°C; ^1^H NMR (400 MHz, CDCl_3_) *δ* 9.04 (d, *J* = 2.8 Hz, 1H), 8.23 (dd, *J* = 9.6, 2.8 Hz, 1H), 6.57 (d, *J* = 9.6 Hz, 1H), 3.88–3.79 (m, 4H), 3.79–3.70 (m, 4H). ^13^C NMR (75 MHz, DMSO-*d*_*6*_) *δ* 160.2, 145.9, 134.5, 132.8, 105.6, 65.8, 44.7. MS (Mwt.: 209.21) m/z = 210.076 ([M+H] ^+^, bp).

### Synthesis of 6-Morpholinopyridin-3-Amine (3)

To a stirred solution of compound **2** (209 mg, 1 mmol) in methanol (5 ml) were added Pd/C (31.4 mg) and ammonium formate (189 mg, 3 mmol). The mixture was stirred at reflux for 3 h, and then cooled to room temperature and filtered through a pad of celite. The filtrate was concentrated in vacuo and the residue was purified by silica gel column chromatography (*p*E/EA = 3:1) to yield compound **3** as a red-brown solid: yield 86%; mp: 146.3–149.6°C. ^1^H NMR (400 MHz, CDCl_3_) *δ* 7.80 (d, *J* = 2.8 Hz, 1H), 7.01 (dd, *J* = 8.8, 2.8 Hz, 1H), 6.57 (d, *J* = 8.8 Hz, 1H), 3.95–3.69 (m, 4H), 3.43–3.23 (m, 4H), 3.22–3.05 (s, 2H). ^13^C NMR (75 MHz, DMSO-*d*_*6*_) *δ* 152.8, 138.0, 124.9, 108.7, 66.5, 47.3. MS (Mwt.: 179.22) m/z = 180.104 ([M+H] ^+^, bp).

### Synthesis of Benzyl (6-Morpholinopyridin-3-yl) Carbamate (4)

To a stirred solution of compound **3** (179 mg, 1 mmol) in a mixed solvent of acetone/water (4.5 ml, ratio 2/1) at 0°C were added sodium carbonate (127 mg, 1.2 mmol) and carbobenzoxy chloride (0.17 ml, 1.2 mmol). The solution was stirred at ambient temperature overnight. The residue was concentrated in vacuo and then extracted with CH_2_Cl_2_ (10 ml × 3), and the combined organic layer was concentrated under vacuum to give compound **4** as a brown solid: yield 93%; mp: 163.1–166.0°C; ^1^H NMR (400 MHz, CDCl_3_) *δ* 8.08 (d, *J* = 2.8 Hz, 1H), 7.77 (s, 1H), 7.45–7.29 (m, 5H), 6.62 (d, *J* = 9.2 Hz, 1H), 5.19 (s, 2H), 3.98–3.63 (m, 4H), 3.56–3.30 (m, 4H). ^13^C NMR (75 MHz, DMSO-*d*_*6*_) *δ* 155.7, 153.7, 138.4, 136.6, 129.2, 128.4, 128.0, 127.0, 126.4, 107.0, 65.9, 65.8, 45.7. MS (Mwt.: 313.36) m/z = 314.120 ([M+H] ^+^, bp).

### Synthesis of (*R*)-5-(Hydroxymethyl)-3-(6-Morpholinopyridine -3-yl)Oxazolidin-2-one (5)

To a stirred solution of compound **4** (1.64 g, 5.24 mmol) in anhydrous THF (20 ml) was slowly added n-BuLi (4.2 ml, 10.48 mmol) at –78°C under nitrogen atmosphere. The mixture was stirred under this condition for 1 h, and then a solution of (*R*)-glycidyl butyrate (0.88 ml, 6.23 mmol) in anhydrous THF (5 ml) was added and stirred for 1 h. The resultant solution was warmed to room temperature and stirred for 16 h, then quenched by dropwise saturated ammonium chloride solution (5 ml). The residue was concentrated in vacuo and then extracted with EtOAc (15 ml × 3). The combined organic layer was concentrated under vacuum purified by silica gel column chromatography (EA/PE = 1:1) to give compound **5** as a yellow solid: yield 71%; mp: 160.2–162.9°C; ^1^H NMR (400 MHz, DMSO-*d*_*6*_) *δ* 8.24 (d, *J* = 2.8 Hz, 1H), 7.86 (dd, *J* = 9.2, 2.8 Hz, 1H), 6.89 (d, *J* = 9.2 Hz, 1H), 5.20 (t, *J* = 5.6 Hz, 1H), 4.71–4.66 (m, 1H), 4.06–4.01 (m, 1H), 3.80–3.77 (m, 1H), 3.71–3.69 (m, 4H), 3.65–3.64 (m, 1H), 3.59–3.53 (m, 1H), 3.40–3.38 (m, 4H). ^13^C NMR (75 MHz, DMSO-*d*_*6*_) *δ* 156.0, 154.0, 138.1, 128.9, 126.7, 107.0, 73.5, 65.9, 61.7, 46.1, 45.5. MS (Mwt.: 279.30) m/z = 280.109 ([M+H] ^+^, bp).

**(*R*)-(3-(6-Morpholinopyridin-3-yl)-2-Oxooxazolidin-5-yl)Methyl Methanesulfonate (6)**

To a stirred solution of compound **5** (279 mg, 1 mmol) in anhydrous CH_2_Cl_2_ (5 ml) were added triethylamine (0.28 ml, 2 mmol) and methanesulfonyl chloride (0.12 ml, 1.5 mmol) at 0°C and the mixture was stirred at ambient temperature overnight. The residue was extracted with CH_2_Cl_2_ (5 ml × 3) and organic phase was washed with brine (10 ml × 2) and concentrated in vacuo to yield **6** as a yellow solid: yield 99%; mp: 254.0–254.4°C; ^1^H NMR (400 MHz, DMSO-*d*_*6*_) *δ* 8.23 (d, *J* = 2.8 Hz, 1H), 7.82 (dd, *J* = 9.2, 2.8 Hz, 1H), 6.90 (d, *J* = 9.2 Hz, 1H), 5.02–4.96 (m, 1H), 4.53–4.44 (m, 2H), 4.17–4.12 (m, 1H), 3.80–3.76 (m, 1H), 3.71–3.69 (m, 4H), 3.41–3.39 (m, 4H), 3.26 (s, 3H). ^13^C NMR (101 MHz, DMSO-*d*_*6*_) *δ* 156.8, 154.7, 139.3, 130.1, 126.6, 107.5, 70.8, 70.3, 66.4, 46.6, 45.9, 37.3. MS (Mwt.: 357.38) m/z = 358.099 ([M+H] ^+^, bp).

**(*R*)-(3-(6-Morpholinopyridin-3-yl)-2-Oxooxazolidin-5-yl)Methyl Methanesulfonate (7)**

To a stirred solution of compound **6** (1.8 g, 5.04 mmol) in dioxane (50 ml) were added phthalimide potassium (1.1 g, 6.05 mmol) and sodium carbonate (1.07 g, 10.08 mmol). The mixture was stirred at 80°C for 48 h, and then cooled to room temperature. The residue was concentrated in vacuo and then extracted with EtOAc (50 ml × 3). The combined organic layer was concentrated and then recrystallized with ethyl acetate and petroleum ether to give yield **7** as a light-yellow solid: yield 86%; mp: 206.0–208.1°C; ^1^H NMR (400 MHz, DMSO-*d*_*6*_) *δ* 8.19 (d, *J* = 2.8 Hz, 1H), 7.93–7.86 (m, 4H), 7.77 (dd, *J* = 9.2, 2.8 Hz, 1H), 6.88 (d, *J* = 9.2 Hz, 1H), 4.97–4.90 (m, 1H), 4.18–4.14 (m, 1H), 4.04–3.98 (m, 1H), 3.94–3.85 (m, 2H), 3.71–3.68 (m, 4H), 3.40–3.38 (m, 4H). ^13^C NMR (101 MHz, DMSO-*d*_*6*_) *δ* 168.3, 156.8, 154.7, 139.3, 135.1, 132.0, 130.0, 126.7, 123.7, 107.4, 70.8, 66.4, 48.5, 45.9, 40.1. MS (Mwt.: 408.41) m/z = 409.149 ([M+H] ^+^, bp).

**(*S*)-5-(Aminomethyl)-3-(6-Morpholinopyridin-3-yl) Oxazolidin-2-one (8)**

Compound **7** (0.98 g, 2.4 mmol) was added to methylamine alcohol (10 ml). The mixture was stirred at 60°C for 3 h and then concentrated in vacuo. The residue was extracted with CH_2_Cl_2_ (15 ml × 3) and concentrated under vacuum. The resulting residue was purified on a silica gel column (CH_2_Cl_2_/MeOH = 30:1) to give compound **8** as a white solid: yield 48%; mp: 185.4–187.7°C; ^1^H NMR (400 MHz, DMSO-*d*_*6*_) *δ* 8.24 (d, *J* = 2.8 Hz, 1H), 7.85 (dd, *J* = 9.2, 2.8 Hz, 1H), 6.89 (d, *J* = 9.2 Hz, 1H), 4.67–4.60 (m, 1H), 4.05–4.00 (m, 1H), 3.83–3.80 (m, 1H), 3.71–3.69 (m, 4H), 3.40–3.38 (m, 4H), 3.01 (s, 2H), 2.92–2.82 (m, 2H). ^13^C NMR (75 MHz, DMSO-*d*_*6*_) *δ* 156.1, 154.7, 138.4, 129.2, 126.6, 106.9, 73.7, 65.9, 47.2, 45.5, 43.9. MS (Mwt.: 278.31) m/z = 279.140 ([M+H] ^+^, bp).

### General Procedure for the Synthesis of Title Compounds 9a-h

To a solution of compound **8** (111 mg, 0.4 mmol) in CH_2_Cl_2_ (10 ml) were added acid (0.6 mmol), DMAP (24.4 mg, 0.2 mmol) and EDCI (115 mg, 0.6 mmol). The mixture was stirred at room temperature for 24 h and then concentrated in vacuo. The residue was extracted with CH_2_Cl_2_ (15 ml × 3), and the combined organic layer was washed with brine (20 ml × 2) and concentrated under vacuum. The resulting residue was purified on a silica gel column to give compound **9a-h**.

**(*S*)-*N*-((3-(6-Morpholinopyridin-3-yl)-2-Oxooxazolidin-5-yl) methyl) Nicotinamide (9a)**

Yellow solid; mp: 178.1–180.2°C. ^1^H NMR (400 MHz, DMSO-*d*_*6*_) *δ* 9.04 (t, *J* = 5.6 Hz, 1H), 9.00 (s, 1H), 8.71 (d, *J* = 4.8 Hz, 1H), 8.22 (d, *J* = 2.8 Hz, 1H), 8.20–8.17 (m, 1H), 7.81 (dd, *J* = 9.2, 2.8 Hz, 1H), 7.53–7.50 (m, 1H), 6.88 (d, *J* = 9.2 Hz, 1H), 4.90–4.84 (m, 1H), 4.17–4.12 (m, 1H), 3.87–3.83 (m, 1H), 3.71–3.64 (m, 6H), 3.48–3.34 (m, 4H). ^13^C NMR (75 MHz, DMSO-*d*_*6*_) *δ* 165.6, 156.2, 154.5, 152.0, 148.4, 138.6, 135.0, 129.5, 129.3, 126.4, 123.4, 106.9, 71.5, 65.9, 47.7, 45.5, 42.3. MS (Mwt.: 383.41) m/z = 384.166 ([M+H] ^+^, bp).

**(*S*)-*N*-((3-(6-Morpholinopyridin-3-yl)-2-Oxooxazolidin-5-yl) Methyl) Furan-2-Carboxamide (9b)**

White solid, mp: 173.0–173.4°C. ^1^H NMR (400 MHz, DMSO-*d*_*6*_) *δ* 8.69 (t, *J* = 6.0 Hz, 1H), 8.21 (d, *J* = 2.8 Hz, 1H), 7.85 (d, *J* = 1.6 Hz, 1H), 7.79 (dd, *J* = 9.2, 2.8 Hz, 1H), 7.15 (d, *J* = 3.6 Hz, 1H), 6.88 (d, *J* = 9.2 Hz, 1H), 6.64–6.62 (m, 1H), 4.81 (m, 1H), 4.14–4.10 (m, 1H), 3.83–3.80 (m, 1H), 3.75–3.64 (m, 4H), 3.62–3.53 (m, 2H), 3.45–3.32 (m, 4H). ^13^C NMR (75 MHz, DMSO-*d*_*6*_) *δ* 158.8, 156.7, 155.0, 148.0, 145.7, 139.2, 129.9, 126.9, 114.3, 112.4, 107.4, 72.0, 66.4, 48.2, 46.0, 42.1. MS (Mwt.: 372.38) m/z = 373.110 ([M+H] ^+^, bp).

**(*S*)-*N*-((3-(6-Morpholinopyridin-3-yl)-2-Oxooxazolidin-5-yl)Methyl)-4-(Trifluoromethyl) Benzamide (9c)**

White solid, mp: 226.1–227.7°C. ^1^H NMR (400 MHz, DMSO- *d*_*6*_) *δ* 9.07 (t, *J* = 5.6 Hz, 1H), 8.22 (d, *J* = 2.4 Hz, 1H), 8.05 (d, *J* = 8.0 Hz, 2H), 7.86 (d, *J* = 8.0 Hz, 2H), 7.81 (dd, *J* = 9.2, 2.4 Hz, 1H), 6.88 (d, *J* = 9.2 Hz, 1H), 4.91–4.84 (m, 1H), 4.17–4.12 (m, 1H), 3.86–3.82 (m, 1H), 3.73–3.67 (m, 4H), 3.65–3.62 (m, 2H), 3.42–3.36 (m, 4H). ^13^C NMR (75 MHz, DMSO-*d*_*6*_) *δ* 165.8, 156.2, 154.5, 138.6, 137.8, 131.5, 129.3 (q, *J*_*C-F*_ = 216.8 Hz), 128.2, 126.4, 125.5, 106.9, 71.5, 65.9, 47.7, 45.5, 42.5. MS (Mwt.: 450.42) m/z = 451.119 ([M+H] ^+^, bp).

**(*S*)-*N*-((3-(6-Morpholinopyridin-3-yl)-2-Oxooxazolidin-5-yl)Methyl)-3-Phenylpropanamide (9d)**

White solid, mp: 213.5–214.4°C. ^1^H NMR (400 MHz, DMSO-*d*_*6*_) *δ* 8.24 (t, *J* = 5.6 Hz, 1H), 8.21 (d, *J* = 2.8 Hz, 1H), 7.79 (dd, *J* = 9.2, 2.8 Hz, 1H), 7.29–7.08 (m, 5H), 6.89 (d, *J* = 9.2 Hz, 1H), 4.72–4.66 (m, 1H), 4.04–4.00 (m, 1H), 3.78–3.61 (m, 4H), 3.65–3.61 (m, 1H), 3.48–3.43 (m, 2H), 3.41–3.38(m, 4H), 2.78 (t, *J* = 8.0 Hz, 2H), 2.42 (t, *J* = 8.8 Hz, 2H). ^13^C NMR (75 MHz, DMSO-*d*_*6*_) *δ* 172.2, 156.2, 154.5, 141.1, 138.5, 129.2, 128.2, 128.1, 126.4, 125.8, 107.0, 71.8, 65.9, 47.3, 45.5, 41.3, 36.8, 31.0. MS (Mwt.: 410.47) m/z = 411.185 ([M+H] ^+^, bp).

**(*S*)-*N*-((3-(6-Morpholinopyridin-3-yl)-2-Oxooxazolidin-5-yl)Methyl) Cyclohexanecarboxamide (9e)**

White solid, mp: 183.3–184.9°C. ^1^H NMR (400 MHz, DMSO-*d*_*6*_) *δ* 8.20 (d, *J* = 2.8 Hz, 1H), 8.07 (t, *J* = 5.6 Hz, 1H), 7.79 (dd, *J* = 9.2, 2.8 Hz, 1H), 6.89 (d, *J* = 9.2 Hz, 1H), 4.74–4.68 (m, 1H), 4.08–4.03 (m, 1H), 3.74–3.66 (m, 5H), 3.48–3.41 (m, 1H), 3.40–3.38 (m, 4H), 3.36–3.33 (m, 1H), 2.17–2.10 (m, 1H), 1.66–1.58 (m, 5H), 1.38–0.94 (m, 5H). ^13^C NMR (75 MHz, DMSO-*d*_*6*_) *δ* 176.6, 156.6, 155.0, 138.9, 129.7, 126.9, 107.4, 72.3, 66.4, 47.8, 46.0, 44.3, 41.7, 29.6, 25.9, 25.7. MS (Mwt.: 388.47) m/z = 389.179 ([M+H] ^+^, bp).

***N*-(((*S*)-3-(6-Morpholinopyridin-3-yl)-2-Oxooxazolidin-5-yl)Methyl)Tetrahydrofuran-2-Carboxamide (9f)**

Gray solid, mp: 130.0–132.2°C. ^1^H NMR (400 MHz, DMSO-*d*_*6*_) *δ* 8.21 (d, *J* = 2.8 Hz, 1H), 8.08 (t, *J* = 5.6 Hz, 1H), 7.80 (dd, *J* = 9.2, 2.8, 1.3 Hz, 1H), 6.89 (d, *J* = 9.2 Hz, 1H), 4.78–4.71 (m, 1H), 4.25–4.21 (m, 1H), 4.09–4.04 (m, 1H), 3.91–3.72 (m, 3H), 3.72–3.65 (m, 4H), 3.53–3.42 (m, 2H), 3.42–3.36 (m, 4H), 2.14–2.06 (m, 1H), 1.87–1.68 (m, 3H). ^13^C NMR (75 MHz, DMSO-*d*_*6*_) *δ* 173.4, 156.2, 154.5, 138.5, 129.2, 126.4, 106.9, 77.6, 71.5, 68.5, 65.9, 47.4, 45.5, 41.2, 30.0, 24.8. MS (Mwt.: 476.41) m/z = 377.151 ([M+H] ^+^, bp)

**(*S*)-*N*-((3-(6-Morpholinopyridin-3-yl)-2-Oxooxazolidin-5-yl) Methyl) Acetamide (9g)**

Yellow solid, mp: 148.8–152.6°C. ^1^H NMR (400 MHz, DMSO-*d*_*6*_) *δ* 8.24–8.21 (m, 2H), 7.80 (dd, *J* = 9.2, 2.8 Hz, 1H), 6.89 (d, *J* = 9.2 Hz, 1H), 4.74–4.63 (m, 1H), 4.09–4.05 (m, 1H), 3.71–3.67 (m, 5H), 3.42–3.38 (m, 6H), 1.84 (s, 3H). ^13^C NMR (75 MHz, DMSO-*d*_*6*_) *δ* 169.9, 156.2, 154.5, 138.7, 129.4, 126.4, 106.9, 71.8, 65.9, 47.5, 45.5, 41.5, 22.4. MS (Mwt.: 320.35) m/z = 321.155 ([M+H] ^+^, bp).

**(*S*)-3-Methyl-N-((3-(6-Morpholinopyridin-3-yl)-2-Oxooxazolidin-5-yl) Methyl) Butanemide (9h)**

White solid, mp: 184.4–187.9°C. ^1^H NMR (400 MHz, DMSO-*d*_*6*_) *δ* 8.21 (d, *J* = 2.8 Hz, 1H), 8.17 (t, *J* = 5.6 Hz, 1H), 7.80 (dd, *J* = 9.2, 2.8 Hz, 1H), 6.89 (d, *J* = 9.2 Hz, 1H), 4.75–4.69 (m, 1H), 4.10–4.05 (m, 1H), 3.75–3.61 (m, 5H), 3.51–3.40 (m, 2H), 3.43–3.35 (m, 4H), 2.05–1.85 (m, 3H), 0.83 (dd, *J* = 8.4, 6.4 Hz, 6H). ^13^C NMR (75 MHz, DMSO-*d*_*6*_) *δ* 172.3, 156.2, 154.5, 138.5, 129.3, 126.4, 106.9, 71.8, 65.9, 47.4, 45.5, 44.5, 41.2, 25.5, 22.2. MS (Mwt.: 362.43) m/z = 363.133 ([M+H] ^+^, bp).

## General Procedure for the Synthesis of Title Compounds 9i, 9j

To a solution of compound **8** (111 mg, 0.4 mmol) in CH_2_Cl_2_ (10 ml) were added sulfochlorides (0.42 mmol), DMAP (9.8 mg, 0.08 mmol) and triethylamine (0.11 ml, 0.8 mmol) at 0°C and the mixture was stirred at ambient for 3 h. The residue was extracted with CH_2_Cl_2_ (10 ml × 3), concentrated, and recrystallized to yield compounds **9i, 9j**.

**(*R*)-*N*-((3-(6-Morpholinopyridin-3-yl)-2-Oxooxazolidin-5-yl) Methyl) Methanesulfonamide (9i)**

White solid, mp: 248.7–249.8°C. ^1^H NMR (400 MHz, DMSO-*d*_*6*_) *δ* 8.23 (d, *J* = 2.8 Hz, 1H), 7.82 (dd, *J* = 9.2, 2.8 Hz, 1H), 7.48 (t, *J* = 6.4 Hz, 1H), 6.90 (d, *J* = 9.2 Hz, 1H), 4.79–4.72 (m, 1H), 4.12–4.08 (m, 1H), 3.79–3.75 (m, 1H), 3.74–3.62 (m, 4H), 3.46–3.33 (m, 4H), 3.35–3.25 (m, 2H), 2.95 (s, 3H). ^13^C NMR (75 MHz, DMSO-*d*_*6*_) *δ* 156.2, 154.5, 138.7, 129.5, 126.4, 107.0, 71.7, 65.9, 47.2, 45.5, 45.1, 39.9. MS (Mwt.: 356.40) m/z = 358.335 ([M+H] ^+^, bp).

**(*R*)-*N*-((3-(6-Morpholinopyridin-3-yl)-2-Oxooxazolidin-5-yl) Methyl) Benzenesulfonamide (9j)**

Yellow solid, mp: 121.3–123.8°C. ^1^H NMR (400 MHz, DMSO-*d*_*6*_) *δ* 8.27–8.11 (m, 2H), 7.93–7.74 (m, 3H), 7.70–7.50 (m, 3H), 6.90 (d, *J* = 9.2 Hz, 1H), 4.75–4.67 (m, 1H), 4.10–4.03 (m, 1H), 3.78–3.73 (m, 1H), 3.79–3.62 (m, 4H), 3.41–3.38 (m, 4H), 3.13–3.10 (m, 2H). ^13^C NMR (75 MHz, DMSO-*d*_*6*_) *δ* 156.7, 154.8, 140.9, 139.1, 133.0, 129.9, 129.8, 126.9, 126.8, 107.5, 72.0, 66.4, 47.6, 46.0, 45.8. MS (Mwt.: 418.47) m/z = 419.133 ([M+H] ^+^, bp).

## General Procedure for the Synthesis of Title Compounds 9k, 9L

To a solution of compound **8** (111 mg, 0.4 mmol) in CH_2_Cl_2_ (10 ml) were added isocyanate (0.8 mmol) and triethylamine (0.11 ml, 0.8 mmol) at 0°C and the mixture was stirred at ambient for 3 h. The residue was extracted with CH_2_Cl_2_ (10 ml × 3), concentrated, and recrystallized to yield compounds **9k**, **9l**.

**(*S*)-1-Cyclohexyl-3-((3-(6-Morpholinopyridin-3-yl)-2-Oxooxazolidin-5-yl) Methyl)urea (9k)**

White solid, mp: 181.4–185.3°C. ^1^H NMR (400 MHz, DMSO-*d*_*6*_) *δ* 8.22 (d, *J* = 2.8 Hz, 1H), 7.80 (dd, *J* = 9.2, 2.8 Hz, 1H), 6.88 (d, *J* = 9.2 Hz, 1H), 6.07 (t, *J* = 6.0 Hz, 1H), 5.90 (d, *J* = 8.0 Hz, 1H), 4.71–4.65 (m, 1H), 4.07–4.03 (m, 1H), 3.76–3.61 (m, 5H), 3.44–3.35 (m, 4H), 3.37–3.31 (m, 3H), 1.81–1.37 (m, 5H), 1.30–0.90 (m, 5H). ^13^C NMR (75 MHz, DMSO-*d*_*6*_) *δ* 157.4, 156.2, 154.6, 138.7, 129.3, 126.5, 106.9, 72.6, 65.9, 47.7, 47.3, 45.5, 42.1, 33.1, 25.3, 24.3. MS (Mwt.: 403.48) m/z = 404.184 ([M+H] ^+^, bp).

**(*S*)-1-(4-Chlorophenyl)-3-((3-(6-Morpholinopyridin-3-yl)-2-Oxooxazolidin-5-yl)Methyl)urea (9l)**

White solid, mp: 229.1–229.5°C. ^1^H NMR (400 MHz, DMSO-*d*_*6*_) *δ* 8.81 (s, 1H), 8.24 (d, *J* = 2.0 Hz, 1H), 7.82 (dd, *J* = 9.2, 2.0 Hz, 1H), 7.65 (s, 1H), 7.31–7.09 (m, 2H), 6.96–6.87 (m, 2H), 6.59 (t, *J* = 5.6 Hz, 1H), 4.81–4.75 (m, 1H), 4.13–4.08 (m, 1H), 3.80–3.72 (m, 1H), 3.72–3.67 (m, 4H), 3.49–3.46 (m, 2H), 3.42–3.35 (m, 4H). ^13^C NMR (75 MHz, DMSO-*d*_*6*_) *δ* 155.2, 152.2, 141.0, 138.6, 133.2, 130.3, 121.7, 120.9, 117.7, 116.8, 106.9, 72.2, 65.9, 47.3, 45.5, 42.0. MS (Mwt.: 431.88) m/z = 432.105 ([M+H] ^+^, bp).

## General Procedure for the Synthesis of Title Compounds 17a-l

The synthesis of compounds **17a-l** was adapted from previously described methodology as **9a-l**.

**(*S*)-*N*-((2-oxo-3-(6-(4-Propionylpiperazin-1-yl)Pyridin-3-yl)Oxazolidin-5-yl)Methyl)a-Cetamide (17a)**

Brown solid, mp: 105.4–108.5°C. ^1^H NMR (400 MHz, DMSO-*d*_*6*_) *δ* 8.25 (t, *J* = 6.0 Hz, 1H), 8.22 (d, *J* = 2.8 Hz, 1H), 7.81 (dd, *J* = 9.2, 2.8 Hz, 1H), 6.92 (d, *J* = 9.2 Hz, 1H), 4.73–4.69 (m, 1H), 4.09–4.06 (m, 1H), 3.71–3.68 (m, 1H), 3.60–3.50 (m, 4H), 3.50–3.49 (m, 2H), 3.44–3.40 (m, 4H), 2.36 (q, *J* = 7.2 Hz, 2H), 1.84 (s, 3H), 1.01 (t, *J* = 7.2 Hz, 3H). ^13^C NMR (75 MHz, DMSO-*d*_*6*_) *δ* 170.6, 155.6, 150.6, 139.0, 129.6, 127.0, 125.2, 108.0, 72.3, 47.9, 45.8, 44.9, 42.0, 22.9. MS (Mwt.: 375.43) m/z = 376.188 ([M+H] ^+^, bp).

**(*S*)-*N*-((3-(6-(4-(Cyclohexanecarbonyl)Piperazin-1-yl)Pyridin-3-yl)-2-oxo-Oxazolidin-5-yl)Methyl)Acetamide (17b)**

Brown solid, mp: 164.7–165.3°C. ^1^H NMR (400 MHz, DMSO-*d*_*6*_) *δ* 8.25 (t, *J* = 6.0 Hz, 1H), 8.22 (d, *J* = 2.8 Hz, 1H), 7.81 (dd, *J* = 9.2, 2.8 Hz, 1H), 6.92 (d, *J* = 9.2 Hz, 1H), 4.73–4.69 (m, 1H), 4.09–4.06 (m, 1H), 3.71–3.68 (m, 1H), 3.65–3.51 (m, 5H), 3.49–3.48 (m, 2H), 3.42–3.38 (m, 4H), 1.84 (s, 3H), 1.74–1.53 (m, 5H), 1.35–1.29 (m, 5H). ^13^C NMR (75 MHz, DMSO-*d*_*6*_) *δ* 170.6, 156.2, 155.1, 141.8, 139.2, 128.9, 128.7, 107.7, 72.3, 48.0, 45.6, 45.4, 44.9, 42.0, 41.1, 34.4, 31.2, 22.9. MS (Mwt.: 429.52) m/z = 430.208 ([M+H] ^+^, bp).

**(*S*)-*N*-((2-oxo-3-(6-(4-(4-(Trifluoromethyl)Benzoyl)Piperazin-1-yl)Pyridin-3-yl)Oxazo-lidin-5-yl)Methyl)Acetamide (17c)**

White solid, mp: 167.3–169.1°C. ^1^H NMR (400 MHz, DMSO-*d*_*6*_) *δ* 8.24 (t, *J* = 5.6 Hz, 1H), 8.23 (d, *J* = 2.8 Hz, 1H), 7.84 (d, *J* = 8.0 Hz, 2H), 7.82 (dd, *J* = 9.2, 2.8 Hz, 1H), 7.68 (d, *J* = 8.0 Hz, 2H), 6.92 (d, *J* = 9.2 Hz, 1H), 4.73–4.69 (m, 1H), 4.09–4.06 (m, 1H), 3.78–3.72 (m, 2H), 3.71–3.68 (m, 1H), 3.56–3.52 (m, 4H), 3.42–3.40 (m, 4H), 1.84 (s, 3H). ^13^C NMR (75 MHz, DMSO-*d*_*6*_) *δ* 170.6, 158.6, 155.7, 155.0, 139.4, 138.9, 133.5, 129.9 (q, *J*_C-F_ = 251.2 Hz), 129.0, 127.2, 108.0, 72.3, 46.2, 45.9, 44.9, 42.0, 22.9. MS (Mwt.: 491.47) m/z = 492.133 ([M+H] ^+^, bp).

**(*S*)-*N*-((3-(6-(4-(6-Chloronicotinoyl)Piperazin-1-yl)Pyridin-3-yl)-2-Oxooxazolidin-5-yl)Methyl)acetamide (17d)**

Yellow oil, ^1^H NMR (400 MHz, DMSO-*d*_*6*_) *δ* 8.52 (d, *J* = 2.4 Hz, 1H), 8.25 (t, *J* = 6.0 Hz, 1H), 8.23 (d, *J* = 2.8 Hz, 1H), 7.96 (dd, *J* = 8.0, 2.4 Hz, 1H), 7.82 (dd, *J* = 9.2, 2.8 Hz, 1H), 7.64 (d, *J* = 8.0 Hz, 1H), 6.93 (d, *J* = 9.2 Hz, 1H), 4.73–4.69 (m, 1H), 4.09–4.06 (m, 1H), 3.71–3.68 (m, 1H), 3.61–3.55 (m, 2H), 3.48–3.45 (m, 4H), 3.42–3.40 (m, 4H), 1.84 (s, 3H). ^13^C NMR (75 MHz, DMSO-*d*_*6*_) *δ* 170.6, 163.3, 155.7, 155.0, 138.9, 130.3, 129.9, 127.0, 126.6, 115.1, 108.0, 72.3, 56.2, 47.9, 44.9, 42.0, 22.9. MS (Mwt.: 458.90) m/z = 459.243 ([M+H] ^+^, bp).

**(*S*)-*N*-((3-(6-(4-(Furan-2-Carbonyl)Piperazin-1-yl)Pyridin-3-yl)-2-Oxooxazolidin-5-yl)Methyl)Acetamide (17e)**

White solid, mp: 191.2–191.8°C. ^1^H NMR (400 MHz, DMSO-*d*_*6*_) *δ* 8.25 (t, *J* = 6.0 Hz, 1H), 8.23 (d, *J* = 2.8 Hz, 1H), 7.87 (d, *J* = 1.6 Hz, 1H), 7.82 (dd, *J* = 9.2, 2.8 Hz, 1H), 7.04 (d, *J* = 3.6 Hz, 1H), 6.93 (d, *J* = 9.2 Hz, 1H), 6.65 (dd, *J* = 3.6, 1.6 Hz, 1H), 4.73–4.69 (m, 1H), 4.09–4.06 (m, 1H), 3.78–3.74 (m, 2H), 3.71–3.69 (m, 1H), 3.69–3.46 (m, 4H), 3.42–3.40 (m, 4H), 1.85 (s, 3H). 13C NMR (75 MHz, DMSO-*d*_*6*_) *δ* 170.7, 166.4, 156.1, 155.1, 151.5, 148.7, 139.1, 131.5, 130.0, 126.9, 124.8, 107.8, 72.3, 48.0, 46.2, 42.0, 22.9. MS (Mwt.: 413.43) m/z = 414.139 ([M+H] ^+^, bp).

**(*S*)-*N*-((2-oxo-3-(6-(4-(3-Phenylpropanoyl)Piperazin-1-yl)Pyridin-3-yl)Oxazolidin-5-yl)Methyl)Acetamide (17f)**

Gray solid, mp: 133.2–133.3°C. ^1^H NMR (400 MHz, DMSO-*d*_*6*_) *δ* 8.25 (t, *J* = 6.0 Hz, 1H), 8.21 (d, *J* = 2.8 Hz, 1H), 7.80 (dd, *J* = 9.2, 2.8 Hz, 1H), 7.31–7.23 (m, 5H), 6.90 (d, *J* = 9.2 Hz, 1H), 4.73–4.69 (m, 1H), 4.09–4.06 (m, 1H), 3.70–3.68 (m, 1H), 3.65–3.49 (m, 6H), 3.42–3.40 (m, 4H), 2.84 (t, *J* = 7.6 Hz, 2H), 2.67 (t, *J* = 7.6 Hz, 2H), 1.84 (s, 3H). ^13^C NMR (75 MHz, DMSO-*d*_*6*_) *δ* 170.5, 164.7, 154.8, 151.7, 149.9, 148.2, 145.4, 133.0, 129.5, 115.4, 114.5, 113.0, 72.6, 48.5, 47.6, 45.3, 41.9, 22.9. MS (Mwt.: 451.53) m/z = 452.190 ([M+H] ^+^, bp).

**(*S,E*)-*N*-((3-(6-(4-(3-(Furan-2-yl)Acryloyl)Piperazin-1-yl)Pyridin-3-yl)-2-Oxooxazolidin-5-yl)Methyl)Acetamide (17g)**

Yellow solid, mp: 182.1–184.2°C. ^1^H NMR (400 MHz, DMSO-*d*_*6*_) *δ* 8.26 (t, *J* = 6.0 Hz, 1H), 8.23 (d, *J* = 2.8 Hz, 1H), 7.84–7.71 (m, 2H), 7.37 (d, *J* = 15.2 Hz, 1H), 6.97 (d, *J* = 15.2 Hz, 1H), 6.93 (d, *J* = 9.2 Hz, 1H), 6.88 (d, *J* = 3.2 Hz, 1H), 6.64–6.60 (m, 1H), 4.73–4.69 (m, 1H), 4.09–4.06 (m, 1H), 3.76–3.72 (m, 2H), 3.73–3.63 (m, 4H), 3.53–3.50 (m, 5H), 1.85 (s, 3H). ^13^C NMR (75 MHz, DMSO-*d*_*6*_) *δ* 174.2, 170.6, 156.3, 155.1, 139.2, 130.0, 126.8, 107.7, 72.3, 48.0, 42.0, 29.6, 26.1, 25.6, 22.9. MS (Mwt.: 439.47) m/z = 440.164 ([M+H] ^+^, bp).

**(*S*)-*N*-((2-oxo-3-(6-(4-((4-(Trifluoromethyl)Phenyl)Sulfonyl)Piperazin-1-yl)Pyridin-3-yl)Oxazolidin-5-yl)Methyl)Acetamide (17h)**

Brown solid, mp: 226.4–226.9°C. ^1^H NMR (400 MHz, DMSO-*d*_*6*_) *δ* 8.23 (t, *J* = 6.0 Hz, 1H), 8.18 (d, *J* = 2.8 Hz, 1H), 8.04 (d, *J* = 8.4 Hz, 2H), 7.99 (d, *J* = 8.4 Hz, 2H), 7.78 (dd, *J* = 9.2, 2.8 Hz, 1H), 6.89 (d, *J* = 9.2 Hz, 1H), 4.71–4.67 (m, 1H), 4.06–4.03 (m, 1H), 3.68–3.65 (m, 1H), 3.58–3.56 (m, 4H), 3.40–3.38 (m, 2H), 3.06–3.04 (m, 4H), 1.83 (s, 3H). ^13^C NMR (75 MHz, DMSO-*d*_*6*_) *δ* 170.7, 159.0, 156.2, 155.1, 146.3, 139.2, 130.1, 126.8, 116.3 (q, *J*_C-F_ = 261.0 Hz), 111.8, 107.7, 72.3, 48.0, 46.2, 45.6, 42.0, 22.9. MS (Mwt.: 527.52) m/z = 528.142 ([M+H] ^+^, bp).

**(*S*)-*N*-((3-(6-(4-((4-Methoxyphenyl)Sulfonyl)Piperazin-1-yl)Pyridin-3-yl)-2Ooxooxazolidin-5-yl)Methyl)Acetamide (17i)**

Brown solid, mp: 175.8–177.1°C. ^1^H NMR (400 MHz, DMSO-*d*_*6*_) *δ* 8.23 (t, *J* = 6.0 Hz, 1H), 8.17 (d, *J* = 2.8 Hz, 1H), 7.77 (dd, *J* = 9.2, 2.8 Hz, 1H), 7.69 (d, *J* = 8.8 Hz, 2H), 7.16 (d, *J* = 8.8 Hz, 2H), 6.87 (d, *J* = 9.2 Hz, 1H), 4.71–4.67 (m, 1H), 4.06–4.03(m, 1H), 3.84 (s, 3H), 3.68–3.66 (m, 1H), 3.57–3.55 (m, 4H), 3.40–3.39 (m, 2H), 2.94–2.92 (m, 4H), 1.83 (s, 3H). ^13^C NMR (75 MHz, DMSO-*d*_*6*_) *δ* 170.6, 157.4, 156.5, 155.1, 139.1, 130.0, 126.6, 107.7, 72.3, 49.7, 48.0, 45.4, 43.5, 33.6, 25.6. MS (Mwt.: 489.55) m/z = 490.174 ([M+H] ^+^, bp).

**(*S*)-*N*-((3-(6-(4-((4-Nitrophenyl)Sulfonyl)Piperazin-1-yl)Pyridin-3-yl)-2-Oxooxazolidin-5-yl)Methyl)Acetamide (17j)**

Brown solid, mp: 209.0–209.3°C. ^1^H NMR (400 MHz, DMSO-*d*_*6*_) *δ* 8.44 (d, *J* = 8.8 Hz, 2H), 8.24 (t, *J* = 6.0 Hz, 1H), 8.17 (d, *J* = 2.8 Hz, 1H), 8.04 (d, *J* = 8.8 Hz, 2H), 7.77 (dd, *J* = 9.2, 2.8 Hz, 1H), 6.89 (d, *J* = 9.2 Hz, 1H), 4.74–4.67 (m, 1H), 4.05–4.02 (m, 1H), 3.69–3.65 (m, 1H), 3.59–3.57 (m, 4H), 3.42–3.38 (m, 2H), 3.08–3.06 (m, 4H), 1.83 (s, 3H). ^13^C NMR (75 MHz, DMSO-*d*_*6*_) *δ* 170.6, 156.4, 155.3, 140.0, 139.2, 130.0, 128.6, 126.7, 125.8, 121.5, 107.8, 72.3, 48.0, 45.4, 43.8, 42.0, 22.9. MS (Mwt.: 504.52) m/z = 505.151 ([M+H] ^+^, bp).

**(*S*)-4-(5-(5-(Acetamidomethyl)-2-Oxooxazolidin-3-yl)Pyridin-2-yl)-*N*-Cyclohexylpiperazine-1-Carboxamide (17k)**

White solid, mp: 216.0–216.7°C. ^1^H NMR (400 MHz, DMSO-*d*_*6*_) *δ* 8.22 (d, *J* = 2.8 Hz, 1H), 7.80 (dd, *J* = 9.2, 2.8 Hz, 1H), 6.92 (d, *J* = 9.2 Hz, 1H), 6.25 (t, *J* = 6.0 Hz, 1H), 5.67 (d, *J* = 8.0 Hz, 1H), 4.73–4.69 (m, 1H), 4.09–4.05 (m, 1H), 3.72–3.68 (m, 1H), 3.40–3.35 (m, 10H), 1.84 (s, 3H), 1.78–1.47 (m, 5H), 1.32–1.00 (m, 5H). ^13^C NMR (75 MHz, DMSO-*d*_*6*_) *δ* 170.6, 156.2, 151.7, 139.2, 129.4, 126.8, 114.5, 107.7, 72.3, 48.0, 45.9, 45.4, 42.0, 40.4, 22.9. MS (Mwt.: 444.54) m/z = 445.230 ([M+H] ^+^, bp)

**(*S*)-4-(5-(5-(Acetamidomethyl)-2-Oxooxazolidin-3-yl)Pyridin-2-yl)-*N*-(4-Chlorophenyl)Piperazine-1-Carboxamide (17l)**

White solid, mp: 248.2–249.1°C. ^1^H NMR (400 MHz, DMSO-*d*_*6*_) *δ* 8.73 (s, 1H), 8.25 (t, *J* = 6.0 Hz, 1H), 8.23 (d, *J* = 2.8 Hz, 1H), 7.82 (dd, *J* = 9.2, 2.8 Hz, 1H), 7.52 (d, *J* = 8.8 Hz, 2H), 7.29 (d, *J* = 8.8 Hz, 2H), 6.95 (d, *J* = 9.2 Hz, 1H), 4.73–4.69 (m, 1H), 4.09–4.07 (m, 1H), 3.71–3.69 (m, 1H), 3.67–3.53 (m, 4H), 3.52–3.48 (m, 4H), 3.42–3.41 (m, 2H), 1.85 (s, 3H). ^13^C NMR (75 MHz, DMSO-*d*_*6*_) *δ* 170.6, 168.3, 156.2, 155.1, 140.4, 139.1, 130.0, 128.3, 126.9, 126.0, 107.8, 72.4, 48.0, 42.0, 22.9. MS (Mwt.: 472.93) m/z = 473.169 ([M+H] ^+^, bp).

## Synthesis of 3-Fluoro-5-Nitropyridin-2-ol (19)

3-fluoropyridin-2-ol **18** (10 g, 88.5 mmol) was added to concentrated sulfuric acid (66 ml) over 30 min at 0°C and then concentrated nitric acid (12 ml) was added to the mixture over 10 min. The resultant solution was warmed to room temperature and stirred overnight. After that, the reaction solution was poured into ice water (300 ml). The precipitate was filtered off to give compound **19** as a yellow solid: yield 58.5%. mp: 221.1–221.8°C. ^1^H NMR (400 MHz, CDCl_3_) *δ* 9.13 (d, *J* = 2.4 Hz, 1H), 8.31 (dd, *J* = 7.2, 2.4 Hz, 1H).

## Synthesis of 2-Chloro-3-Fluoro-5-Nitropyridine (20)

3-fluoro-5-nitropyridin-2-ol **19** (7.9 g, 50 mmol) was added to POCl_3_ (200 ml) and warmed to 60°C. PCl_5_ (15.6 g, 75 mmol) was added the solution over 50 min. The mixture was stirred at 60°C for 2 h and concentrated under vacuum. The residue was quenched by ice water (40 ml) and extracted with EtOAc (50 ml × 3). The organic phase was washed with saturated sodium carbonate (40 ml × 2) and concentrated in vacuo to yield **20** as a yellow solid. Yield 52.1%. mp: 38.5–39.7°C. ^1^H NMR (400 MHz, CDCl_3_) *δ* 9.13 (d, *J* = 2.4 Hz, 1H), 8.31 (dd, *J* = 7.2, 2.4 Hz, 1H).

## General Procedure for the Synthesis of Title Compounds 21a-f

The synthesis of compounds **21a-f** was adapted from previously described methodology as **17a-l**.

**(*S*)-*N*-((3-(5-Fluoro-6-(4-Propionylpiperazin-1-yl)Pyridin-3-yl)-2-Oxooxazolidin-5-yl)Methyl)Acetamide(21a)**

Brown solid, mp: 173.0–173.6°C. ^1^H NMR (400 MHz, DMSO-*d*_*6*_) *δ* 8.25 (t, *J* = 6.0 Hz, 1H), 8.13 (d, *J* = 2.4 Hz, 1H), 7.92 (dd, *J* = 14.4, 2.4 Hz, 1H), 4.77–4.73 (m, 1H), 3.76–3.68 (m, 1H), 3.59–3.70 (m, 4H), 3.43–3.41 (m, 2H), 3.29–3.28 (m, 1H), 3.11–3.09 (m, 4H), 2.36 (q, *J* = 7.6 Hz, 2H), 1.84 (s, 3H), 1.01 (t, *J* = 7.6 Hz, 3H). ^13^C NMR (75 MHz, DMSO-*d*_*6*_) *δ* 170.6, 152.9 (q, *J*_C-F_ = 257.3 Hz), 139.1, 129.1, 128.6, 125.9, 121.5, 120.3, 72.6, 48.0, 47.6, 46.2, 44.0, 41.9, 22.9, 9.1. MS (Mwt.: 393.42) m/z = 394.178 ([M+H] ^+^, bp).

**(*S*)-*N*-((3-(6-(4-(Cyclohexanecarbonyl)Piperazin-1-yl)-5-Fluoropyridin-3-yl)-2-Oxooxazolidin-5-yl)Methyl)Acetamide(21b)**

Brown solid, mp: 198.1–199.4°C. ^1^H NMR (400 MHz, DMSO-*d*_*6*_) *δ* 8.23 (t, *J* = 6.0 Hz, 1H), 8.13 (d, *J* = 2.0 Hz, 1H), 7.92 (dd, *J* = 14.4, 2.0 Hz, 1H), 4.77–4.73 (m, 1H), 4.13–4.10 (m, 1H), 3.74–3.72 (m, 1H), 3.63–3.57 (m, 4H), 3.43–3.41 (m, 2H), 3.29–3.27 (m, 4H), 2.66–2.56 (m, 1H), 1.84 (s, 3H), 1.75–1.59 (m, 5H), 1.43–1.20 (m, 5H). ^13^C NMR (75 MHz, DMSO-*d*_*6*_) *δ* 174.0, 170.5, 154.8, 150.0 (q, *J*_C-F_ = 257.1 Hz), 145.8, 133.0, 129.9, 115.4, 72.6, 48.6, 47.6, 45.0, 41.9, 41.2, 29.6, 26.1, 25.6, 22.9. MS (Mwt.: 447.51) m/z = 448.213 ([M+H] ^+^, bp).

**(*S*)-*N*-((3-(5-Fluoro-6-(4-(3-Phenylpropanoyl)Piperazin-1-yl)Pyridin-3-yl)-2-Oxooxazolidin-5-yl)Methyl)Acetamide(21c)**

Brown solid, mp: 143.0–143.6°C. ^1^H NMR (400 MHz, DMSO-*d*_*6*_) *δ* 8.28 (t, *J* = 6.0 Hz, 1H), 8.12 (d, *J* = 2.4 Hz, 1H), 7.92 (dd, *J* = 14.4, 2.4 Hz, 1H), 7.30–7.20 (m, 5H), 4.77–4.73 (m, 1H), 4.13–4.11 (m, 1H), 3.75–3.72 (m, 1H), 3.60–3.58 (m, 2H), 3.43–3.38 (m, 4H), 3.28–3.26 (m, 4H), 2.67 (t, *J* = 7.6 Hz, 2H), 2.53 (t, *J* = 7.6 Hz, 2H), 1.84 (s, 3H). ^13^C NMR (75 MHz, DMSO-*d*_*6*_) *δ* 172.0, 170.5, 170.0, 154.8, 150.0 (q, *J*_C-F_ = 256.2 Hz), 148.3, 145.9, 139.0, 133.0, 128.7, 127.9, 115.5, 72.6, 47.6, 46.2, 44.9, 41.9, 26.0, 22.9. MS (Mwt.: 469.52) m/z = 470.202 ([M+H] ^+^, bp).

**(*S,E*)-*N*-((3-(5-Fluoro-6-(4-(3-(Furan-2-yl)Acryloyl)Piperazin-1-yl)Pyridin-3-yl)-2-Oxooxazolidin-5-yl)Methyl)Acetamide(21d)**

White solid, mp: 200.7–202.3°C. ^1^H NMR (400 MHz, DMSO-*d*_*6*_) *δ* 8.16 (t, *J* = 6.0 Hz, 1H), 8.06 (d, *J* = 2.4 Hz, 1H), 7.86 (dd, *J* = 14.4, 2.4 Hz, 1H), 7.73 (d, *J* = 1.6 Hz, 1H), 7.30 (d, *J* = 15.2 Hz, 1H), 6.89 (d, *J* = 15.2 Hz, 1H), 6.81 (d, *J* = 3.2 Hz, 1H), 6.54 (dd, *J* = 3.2, 1.6 Hz, 1H), 4.75–4.58 (m, 1H), 4.06–4.03 (m, 1H), 3.79–3.58 (m, 5H), 3.36–3.33 (m, 2H), 3.31–3.27 (m, 4H), 1.77 (s, 3H). ^13^C NMR (75 MHz, DMSO-*d*_*6*_) *δ* 170.5, 164.6, 154.8, 150.6 (q, *J*_C-F_ = 259.1 Hz), 149.9, 145.7, 138.7, 135.0, 133.0, 131.4, 124.3, 120.7, 72.6, 47.6, 46.1, 41.9, 22.9. MS (Mwt.: 457.46) m/z = 458.183 ([M+H] ^+^, bp).

**(*S,E*)-*N*-((3-(5-Fluoro-6-(4-(3-(Pyridin-3-yl)Acryloyl)Piperazin-1-yl)Pyridin-3-yl)-2-Oxooxazolidin-5-yl)Methyl)Acetamide(21e)**

Brown solid, mp: 201.5–202.9°C. ^1^H NMR (400 MHz, DMSO-*d*_*6*_) *δ* 8.90 (d, *J* = 2.0 Hz, 1H), 8.56 (dd, *J* = 4.8, 2.0 Hz, 1H), 8.25 (t, *J* = 6.0 Hz, 1H), 8.21 (d, *J* = 8.4 Hz, 1H), 8.15 (d, *J* = 2.4 Hz, 1H), 7.94 (dd, *J* = 14.4, 2.4 Hz, 1H), 7.56 (d, *J* = 15.2 Hz, 1H), 7.47 (d, *J* = 15.2 Hz, 1H), 7.46 (dd, *J* = 8.4, 4.8 Hz, 1H), 4.78–4.74 (m, 1H), 4.13–4.10 (m, 1H), 3.90–3.88 (m, 2H), 3.75–3.72 (m, 4H), 3.43–3.41 (m, 4H), 3.12–3.08 (m, 1H), 1.85 (s, 3H). ^13^C NMR (75 MHz, DMSO-*d*_*6*_) *δ* 174.2, 170.6, 158.8, 154.8, 149.9 (q, *J*_C-F_ = 257.0 Hz), 148.2, 145.8, 141.8, 133.0, 130.1, 126.4, 118.7, 116.7, 115.5, 72.6, 45.1, 41.9, 35.7, 34.4, 31.2, 30.8, 22.9. MS (Mwt.: 468.49) m/z = 469.19 ([M+H] ^+^, bp).

**(*S*)-4-(5-(5-(Acetamidomethyl)-2-Oxooxazolidin-3-yl)-3-Fluoropyridin-2-yl)-N-(4-Chlorophenyl)Piperazine-1-Carboxamide(21f)**

Brown solid, mp: 218.7–220.2°C. ^1^H NMR (400 MHz, DMSO-*d*_*6*_) *δ* 8.73 (s, 1H), 8.25 (t, *J* = 6.0 Hz, 1H), 8.14 (d, *J* = 2.4 Hz, 1H), 7.94 (dd, *J* = 14.4, 2.4 Hz, 1H), 7.48 (d, *J* = 8.4 Hz, 2H), 7.33 (d, *J* = 8.4 Hz, 2H), 4.78–4.74 (m, 1H), 4.13–4.10 (m, 1H), 3.75–3.72 (m, 1H), 3.67–3.56 (m, 4H), 3.43–3.41 (m, 2H), 3.40–3.34 (m, 4H), 1.85 (s, 3H). ^13^C NMR (75 MHz, DMSO-*d*_*6*_) *δ* 172.1, 170.6, 158.8, 156.3, 155.1 (q, *J*_C-F_ = 279.8 Hz), 139.2, 130.1, 128.7, 127.9, 126.7, 107.7, 72.3, 48.0, 42.0, 41.1, 26.0, 22.9. MS (Mwt.: 490.92) m/z = 491.180 ([M+H] ^+^, bp).

## MIC Determination

The Minimum Inhibitory Concentrations (MICs) of compounds **9a-l**, **17a-l** and **21a-f** were determined by the broth microdilution method to study their antibacterial activities *in vitro*. The determination method of MICs, including protocol design, preparation of the solution, experimental operation and test criterion, was obtained by combining the methods provided by the Clinical Laboratory Standards Institute (CLSI) and other reported experimental methods (CLSI, 2016; Liu et al., 2019; Yang et al., 2019; Gao et al., 2020).

The compounds **9a-l**, **17a-l** and **21a-f** were prepared into 1,000 g/L stock solution with 100% DMSO. The stock solution of each compound was diluted with deionized water to a concentration of 256 g/L. The MH broths and the solution of each compound at a concentration of 256 g/L were mixed to concentration gradients in each well on the microtitration plates by multiple 2-fold serial dilutions. On the microtitration plates with gradient concentrations, 1 × 10^6^ CFU/ml of bacterial culture (100 μL) solution was added to each well. The mixtures on the microtitration plates were then placed at 37°C and incubated for 16–20 h. Each hole was subsequently analyzed to observe if there was any visible bacterial colony growth at the bottom. The lowest concentration of the tested compound capable of inhibiting the visible growth of the bacteria was known as the MIC. The MIC of each compound against different bacteria was recorded accordingly. DMSO and uninoculated medium were mixed as the blank control, linezolid as oxazolidinone was the positive control, and the target compounds were tested in parallel under the same conditions as all controls. The *in vitro* antibacterial activities of compounds **9a-l**, **17a-l** and **21a-f** were determined and compared with the controls. Five Gram-positive bacteria *S. aureus* ATCC25923, *S. pneumonia* ATCC49619, *E. faecalis* ATCC29212, *B. subtilis* ATCC6633, *S. xylosus* ATCC35924 were used as standard controls for MIC determinations.

## Scanning Electron Microscopy Assay

The determination through SEM analysis, including the experimental design, preparation of bacterial powder, sample gold spraying and observation under the microscope, was obtained by combining the methods provided by other reported experimental methods (Marinelli et al., 2019; Sharma et al., 2017; Zhao et al., 2016; Kaya et al., 2008). The bacteria *S. pneumoniae* ATCC49619 and *S. aureus* ATCC25923 were inoculated on MH agar medium and cultured at 37°C for 18–24 h. The single colonies selected were cultured in MH broth or in 1/2 × MIC concentration, subinhibitory concentration, of compounds **21b**, **21d** and **21f** (20 ml) for 18–24 h at 37°C. All samples were centrifuged at 8,000 r/min × 5 min, then washed with phosphate-buffered saline (0.1 M, pH 7.2). After washing 3 times, bacterial samples were fixed with 2.5% glutaraldehyde (pH 7.2) and then kept at 4°C for 1.5 h. All samples were resuspended with phosphate-buffered saline (0.1 M, pH 7.2) and centrifuged at 8,000 r/min for 10 min at 4°C. After washing 3 times, samples were dehydrated by passing through gradient ethanol (50%, 70%, 90%, and 100%) for 10 min. The dehydrated samples were replaced with a mixture of EtOH and *tert*-butanol (EtOH/*t*-BuOH = 1:1) for 15 min, and then replaced with pure tert-butanol for 15 min twice. The replaced samples were preserved at –20°C for 30 min then dried with an automatic critical point drying instrument (Hitachi ES-2030) for 4 h. The freeze-dried powder was glued on the SEM sample platform with conductive tape, and a metal film of 100–150 Å was coated on the surface with an ion-sputtering coating instrument. All samples were examined under the scanning electron microscope.

## Time-Growth Kinetics Assay

As described previously (Hasan et al., 2019), a time-growth kinetics assay was performed for **21d** against four Gram-positive bacteria. *S. aureus*, *S. pneumonia*, *E. faecalis* and *S. xylosus* of log-phase were inoculated in MH broth, which contained a range of compound **21d** in different concentrations (1/4, 1/2, 1, 2 and 4 × MIC), and 1% DMSO was used as vehicle control and linezolid as a positive drug control in this assay. The mixtures were cultivated at 37°C with slowly shaking (100 rpm). Then, 3 ml samples were removed each hour to monitor O.D. 600 value to draw a time-growth kinetics curve with data of different concentrations and different time points.

## Molecular Docking Studies

The 3D structure of the 50S ribosomal subunit was obtained from the Protein Data Bank (PDB code: 3CPW) and prepared using PyMOL 1.5.0.3. The needed RNA chains were retained and the ligands and proteins were deleted. The 2D and 3D structures of the ligands were drawn with the aid of ChemOffice 2010 Version. The molecular docking process was carried out using the AutoDock 4.2.6^®^ software. The docking results were analyzed and visualized with PyMOL 1.5.0.3.

## Biofilm Formation Inhibition Assay

Biofilm inhibition was tested by crystal violet assay (Brackman et al., 2012): The bacterial strains cultured to log phase were diluted with MH medium to 10^6^ CFU/ml and added to 96-well plates (100 μL per well). A certain amount of the compounds to be measured was added to each well with the final concentration of 128–0.25 μg/ml, with 3 parallel wells for each group. 100 μL bacterial culture was added to the control group, and 100 μL fresh MH medium was added to the blank group. Then the plate was incubated at 37°C for 20 h. After that, the bacterial culture suspension was poured out and the 96-well plate was washed 3 times with PBS buffer before being dried. The biofilms without phytoplankton were fixed in anhydrous methanol for 15 min and then dried naturally. Then the biofilm was stained with 0.1% (W/V) crystal violet solution for 30 min. After pouring away the crystal violet (CV) solution, each well was washed with distilled water three times, and dissolved in 100 μL 33% glacial acetic acid after drying. The absorbance at 600 nm was measured with a Thermo Scientific Microplate Reader to determine the inhibitory effect on biofilms. Each assay was performed in triplicate.

## Multigeneration Resistance Selection Assay

The propensity of bacterial resistance development for **21d** was evaluated using a multi-generation resistance selection assay (Song et al., 2021). The initial MIC of **21d** against *S. pneumoniae* (ATCC49619) was determined by the antibacterial activity evaluation described above. The bacterial strain was inoculated with **21d** in sub-MIC (1/2 × MIC) for subculture at 37°C and the MIC values of the new generation of *S. pneumoniae* against **21d** and linezolid were determined every 24 h. Furthermore, the new generation of *S. pneumoniae* was inoculated into **21d** solution at sub-MIC concentration, and linezolid was used as control. The experiment process lasted 15 days and linezolid was used for comparisons to investigate the resistance development.

## Data Availability

The original contributions presented in the study are included in the article/Supplementary Material, further inquiries can be directed to the corresponding author.
