# Supplementary material for: Synthesis and Biological Evaluation of 3-(Pyridine-3-yl)-2-Oxazolidinone Derivatives as Antibacterial Agents
Source: Front Chem. 2022 Jul 18;10:949813. doi: 10.3389/fchem.2022.949813 (PMC9339906; doi:10.3389/fchem.2022.949813)
Supplement: Supplementary file 1 [file DataSheet1.PDF]

Supporting Information

Synthesis and biological evaluation of 3-(pyridine-3-yl)-2-oxazolidinone derivatives as antibacterial agents

Bo Jin [a], Tong Wang [a], Jiayi Chen [a], Xiaoqing Liu [a], Yixin Zhang [a], Xiuying Zhang [a], Zunlai Sheng [a, b], Hongliang Yang [a, b, *]

[a] *Department of Veterinary Medicine, Northeast Agricultural University, Harbin 150030, PR China*

[b] *Heilongjiang Key Laboratory for Animal Disease Control and Pharmaceutical Development, Harbin 150030, PR China*

* Correspondence: hongl_yang@126.com

Compounds spectral data

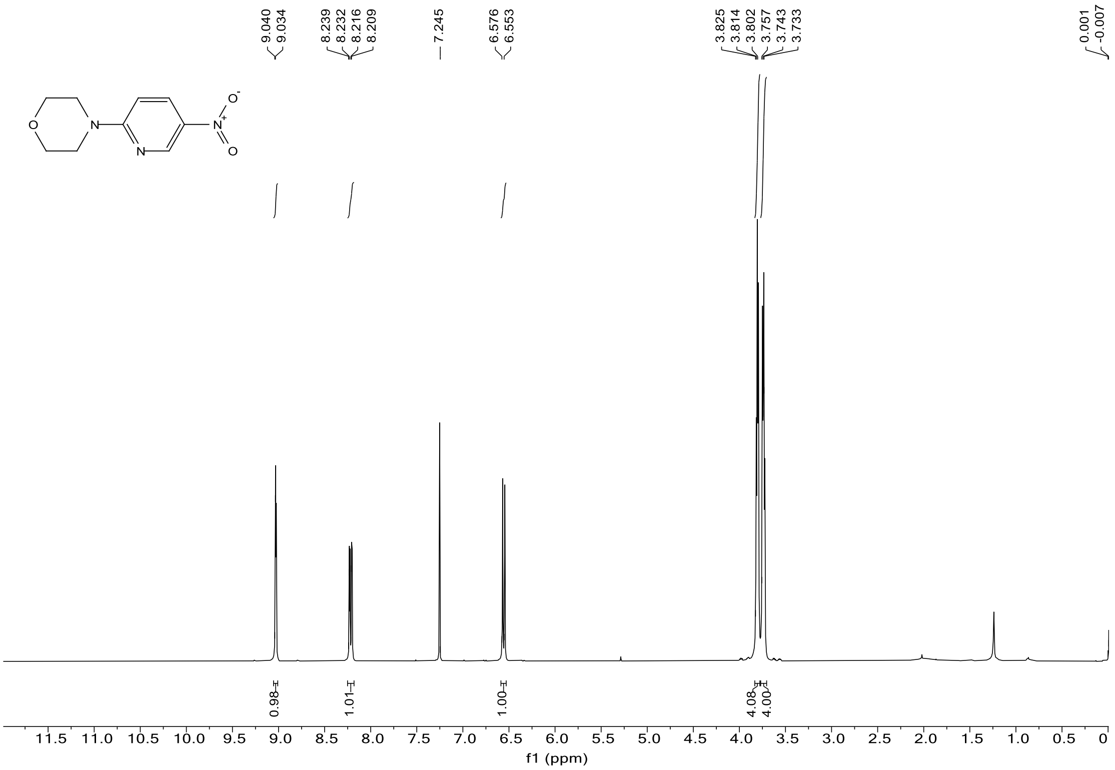

Fig. S1. ^{1}H NMR Spectrum ($CDCl_3$, 400 MHz) of **2**.

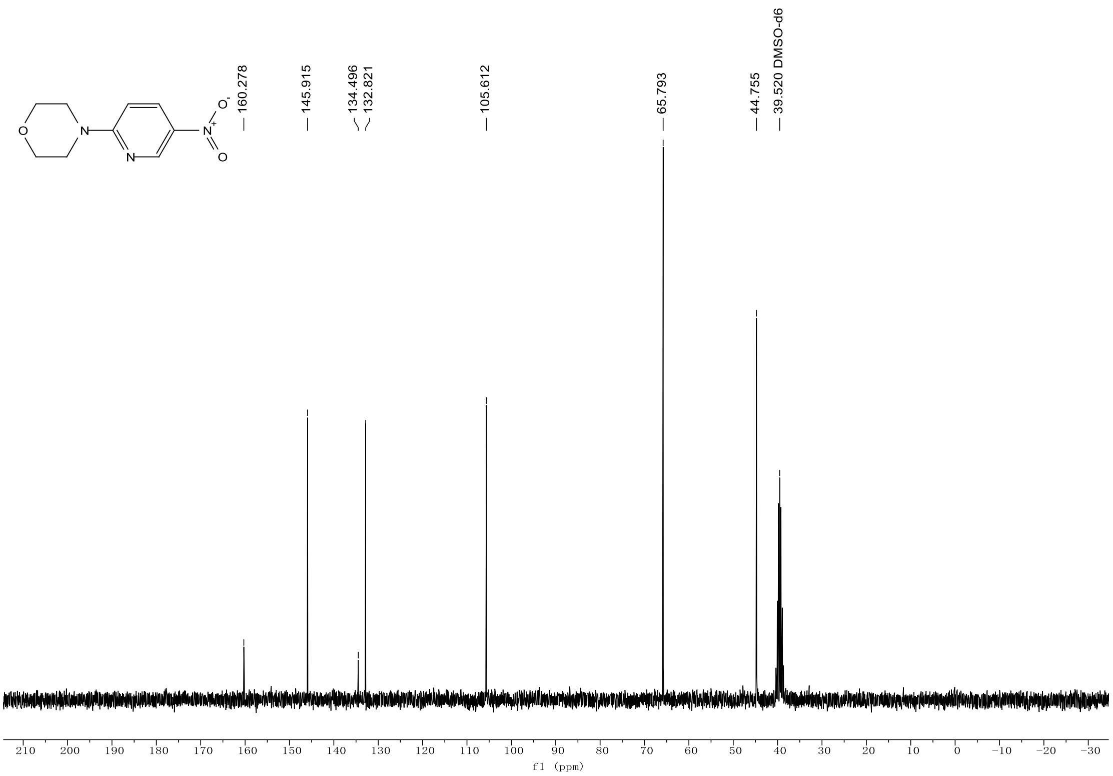

Fig. S2. ^{13}C NMR Spectrum (DMSO-d_6, 75 MHz) of **2**.

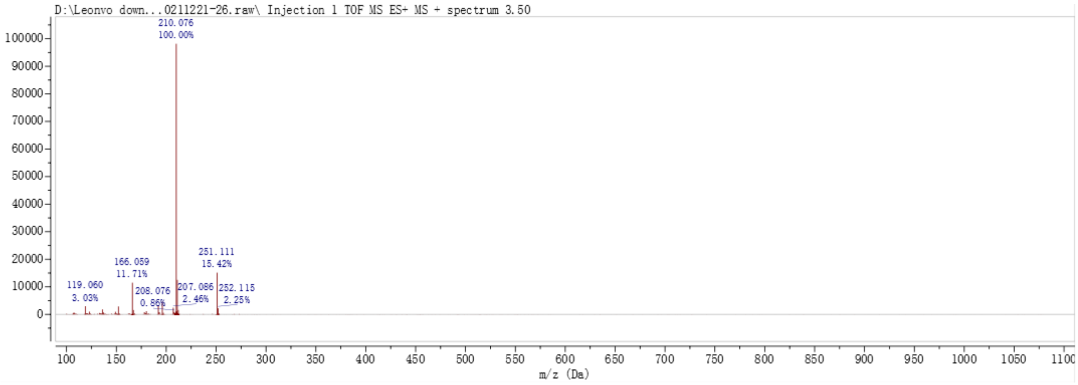

Fig. S3. MS calcd for $C_9H_{11}N_3O_3$ (Mwt.: 209.21): m/z 210.076 ([M+H] $^+$, bp) of **2**.

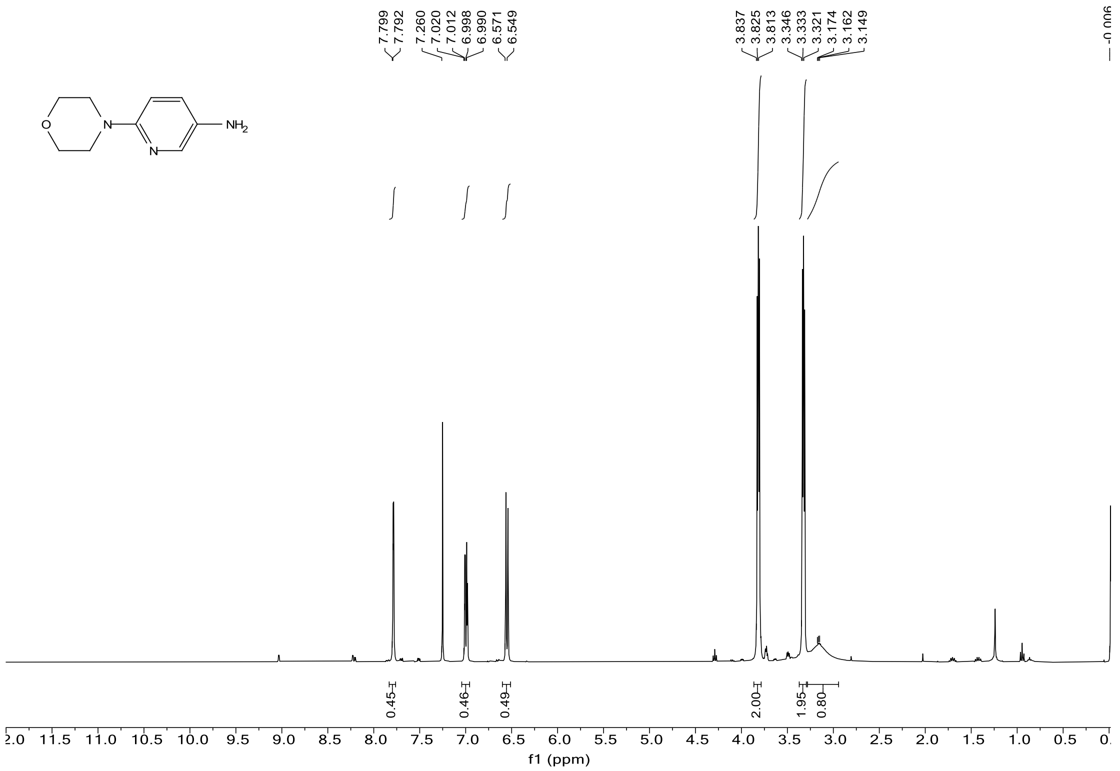

Fig. S4. ^{1}H NMR Spectrum ($CDCl_3$, 400 MHz) of **3**.

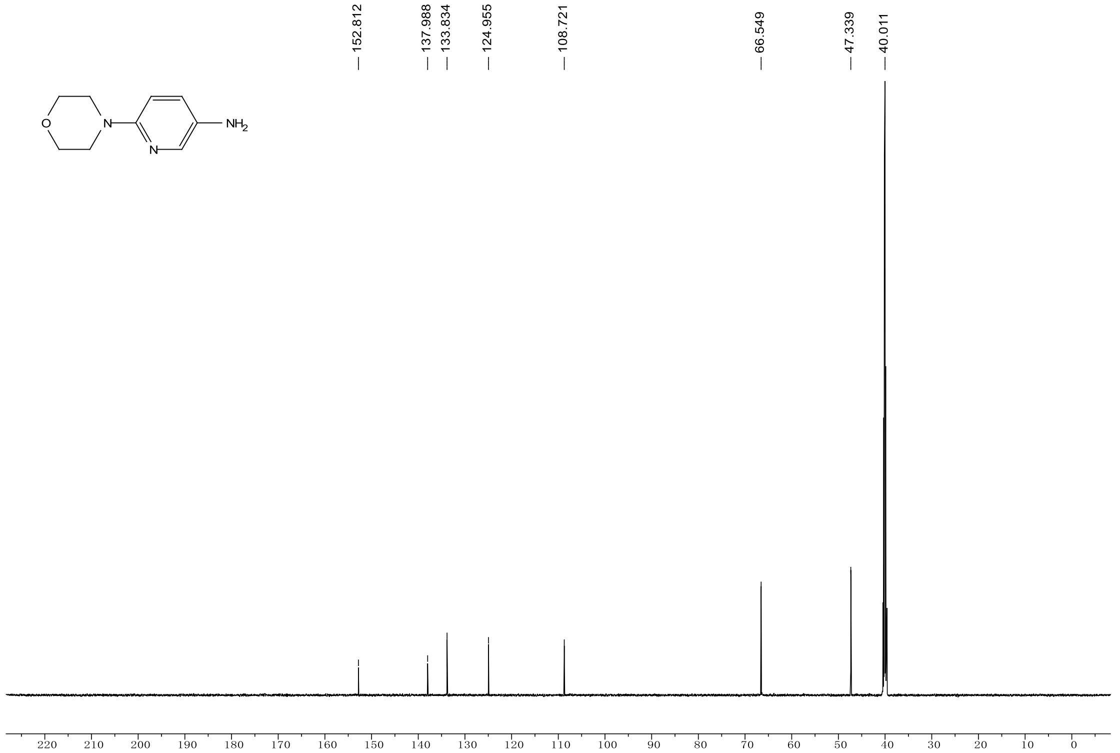

Fig. S5. ^{13}C NMR Spectrum (DMSO-d_6, 75 MHz) of **3**.

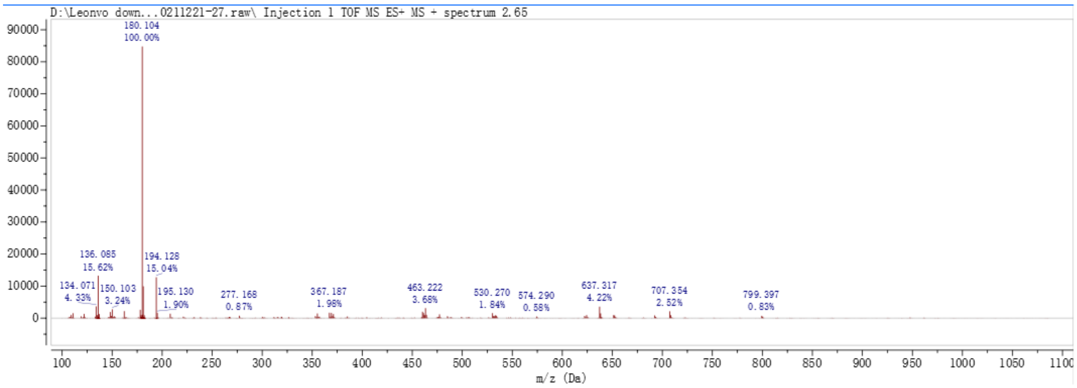

Fig. S6. MS calcd for $C_9H_{13}N_3O$ (Mwt.: 179.22): m/z 180.104 ($[M+H]^+$, bp) of **3**.

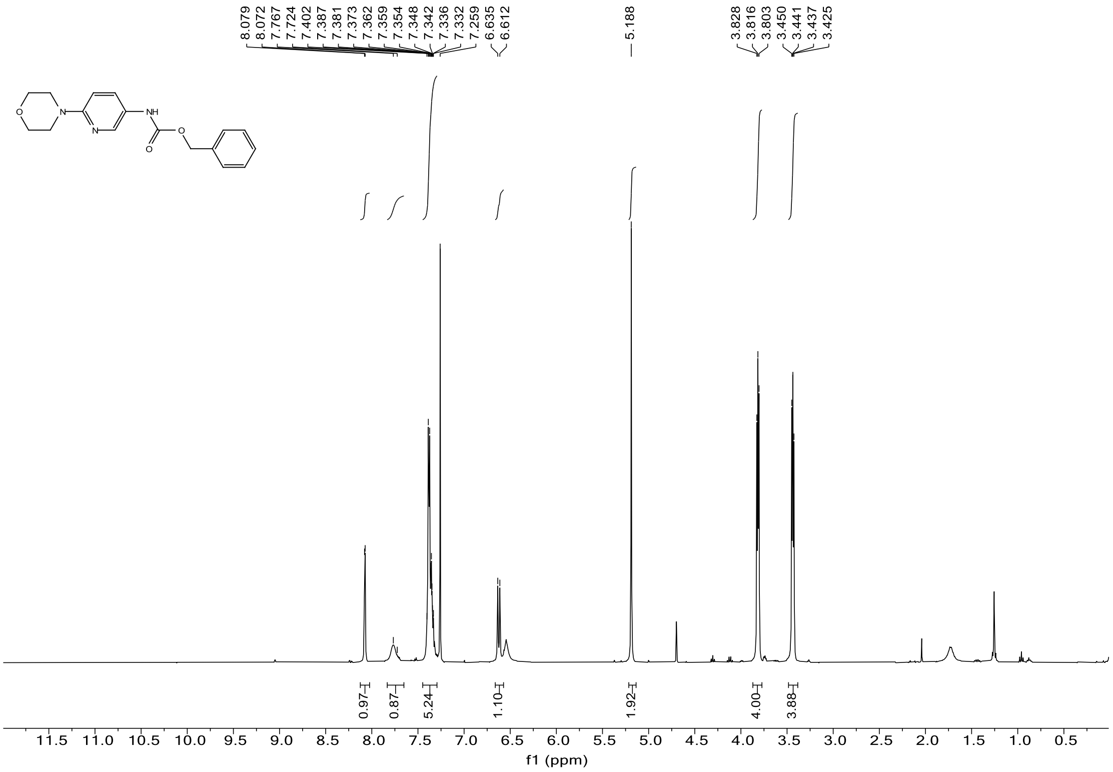

Fig. S7. ^1H NMR Spectrum ($CDCl_3$, 400 MHz) of **4**.

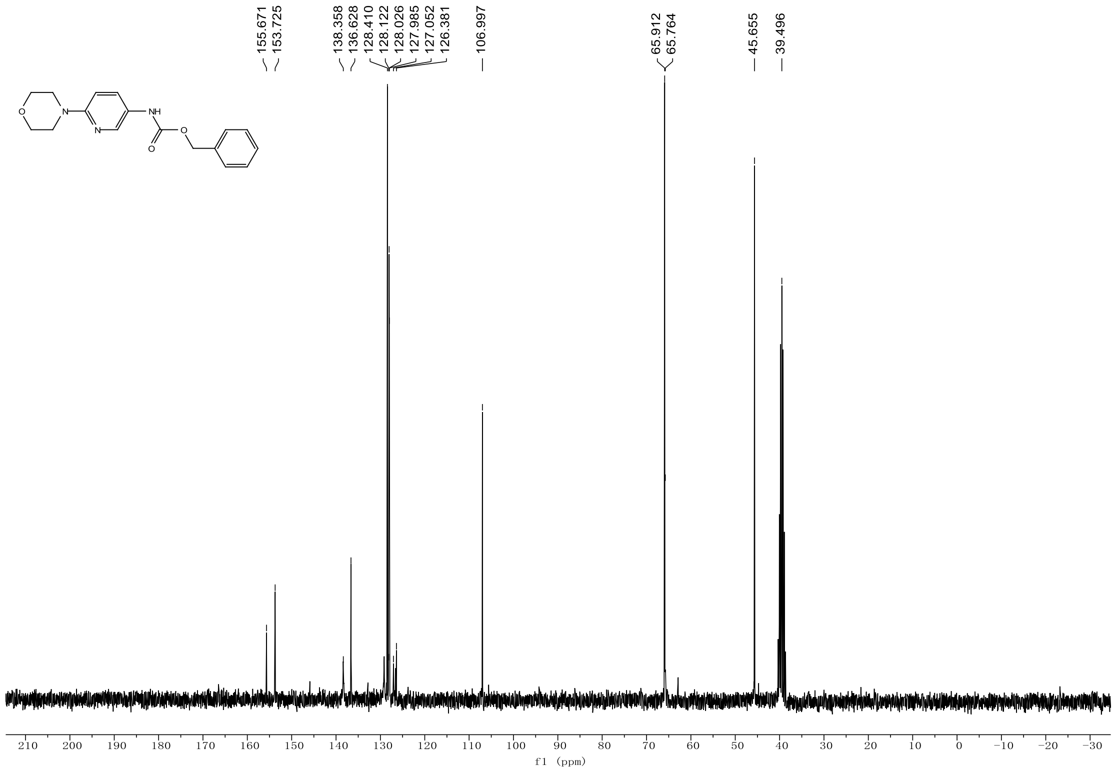

Fig. S8. ^{13}C NMR Spectrum (DMSO-d_6, 75 MHz) of **4**.

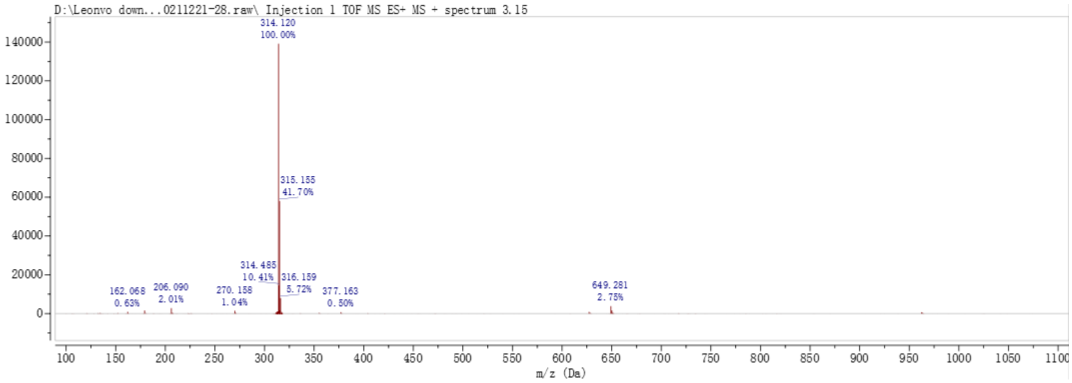

Fig. S9. MS calcd for $C_{17}H_{19}N_3O_3$ (Mwt.: 313.36): m/z 314.120 ([M+H] $^+$, bp) of **4**.

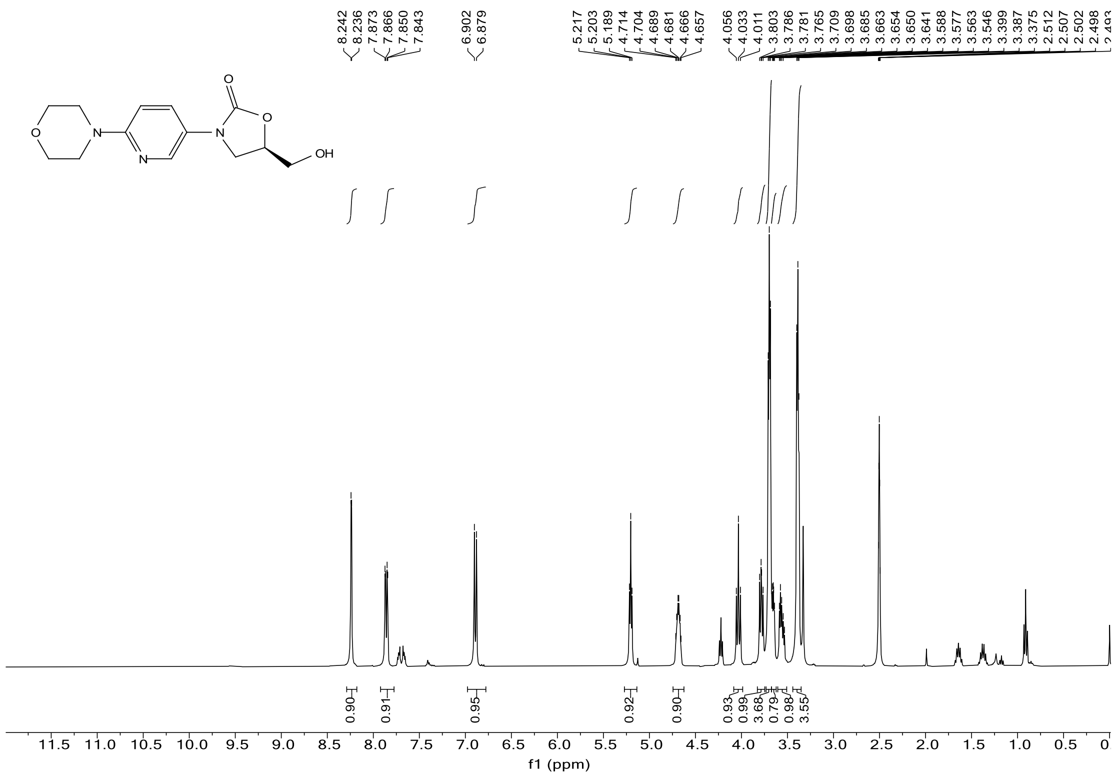

Fig. S10. ^{1}H NMR Spectrum (DMSO-d_6, 400 MHz) of **5**.

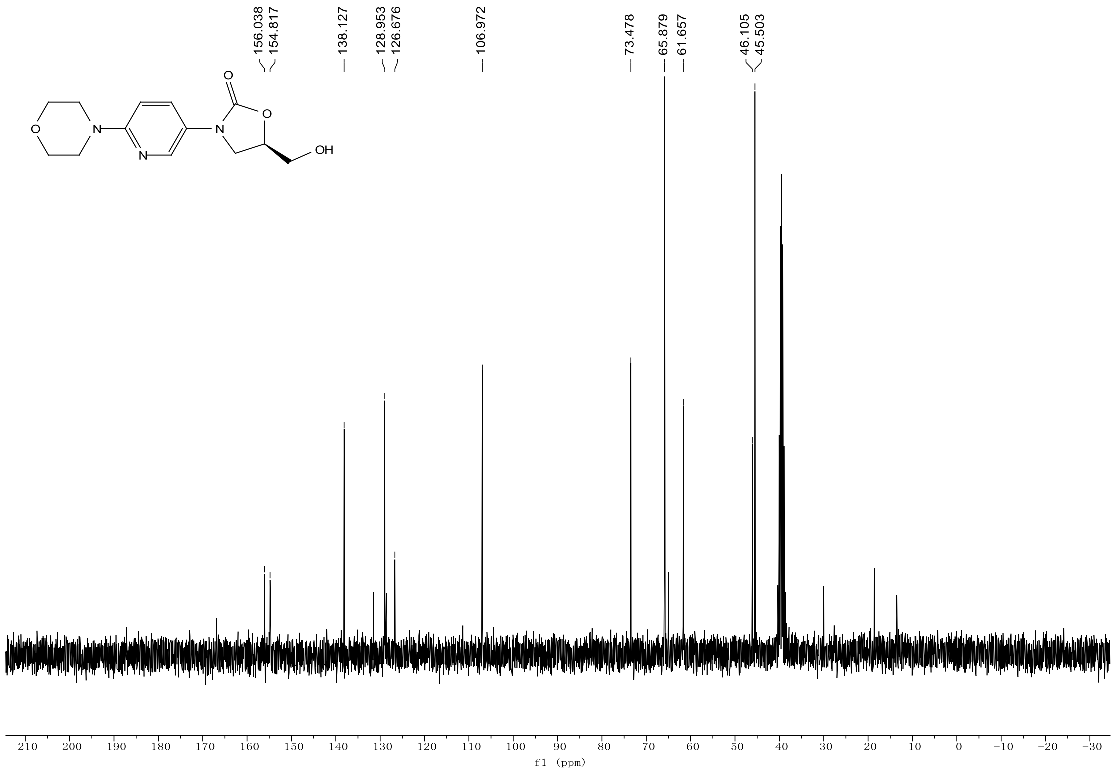

Fig. S11. ^{13}C NMR Spectrum (DMSO-d_6, 75 MHz) of **5**.

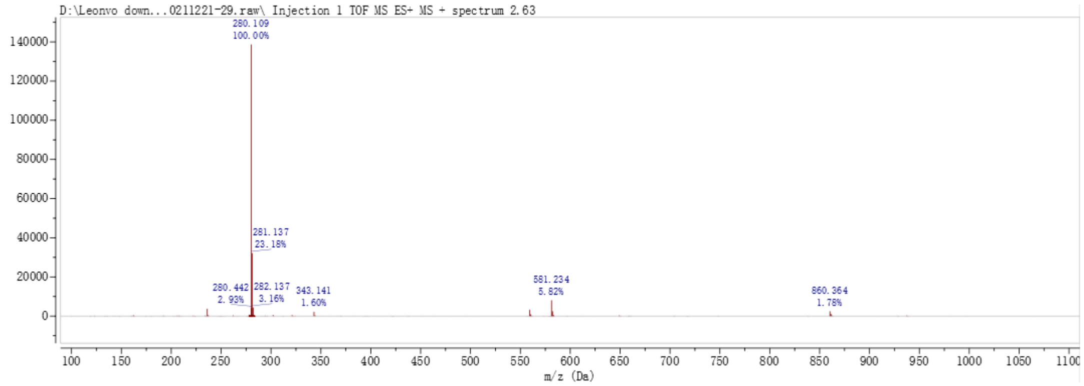

Fig. S12. MS calcd for $C_{13}H_{17}N_3O_4$ (Mwt.: 279.30): m/z 280.109 ([M+H] $^+$, bp) of **5**.

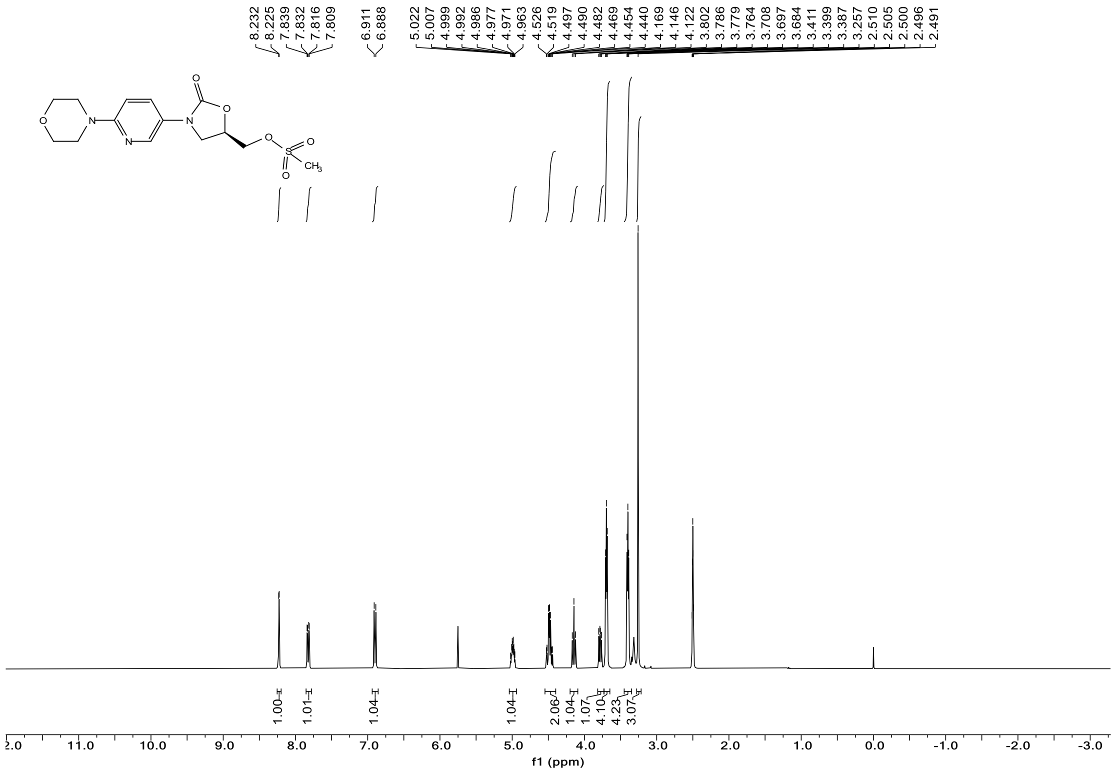

Fig. S13. ^{1}H NMR Spectrum (DMSO-d_6, 400 MHz) of **6**.

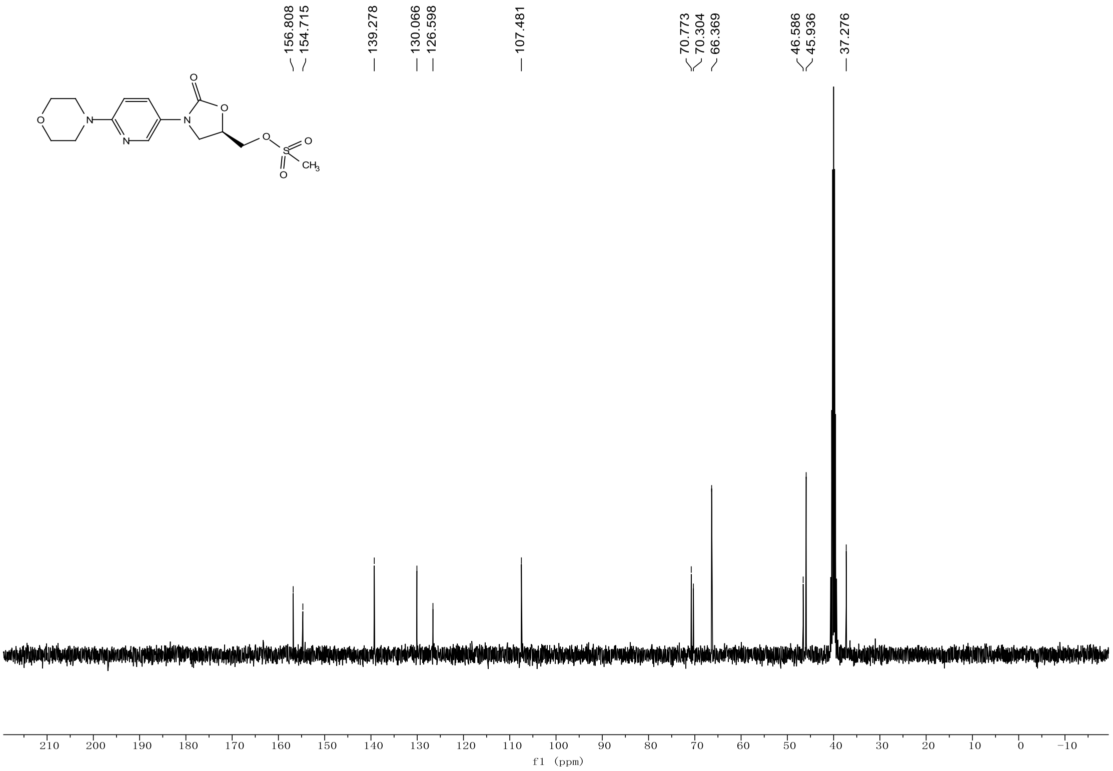

Fig. S14. ^{13}C NMR Spectrum (DMSO-d_6, 101 MHz) of **6**.

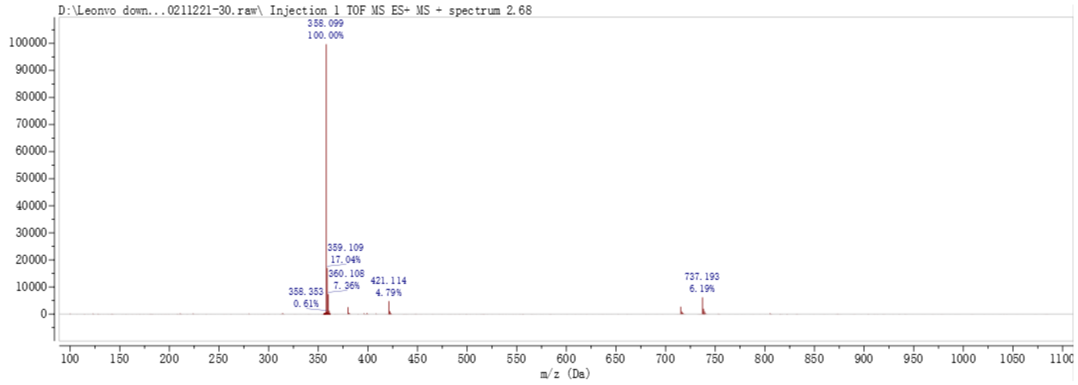

Fig. S15. MS calcd for $C_{14}H_{19}N_3O_6S$ (Mwt.: 357.38): m/z 358.099 ([M+H] $^+$, bp) of **6**.

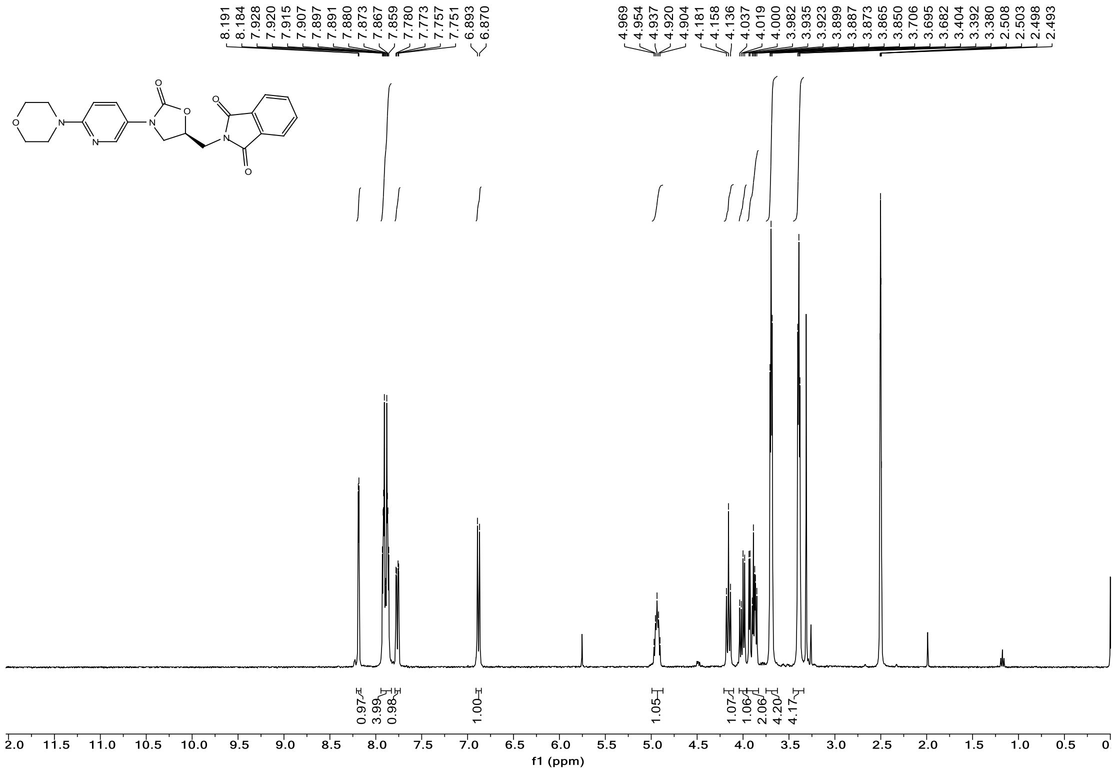

Fig. S16. ^{1}H NMR Spectrum (DMSO-d_6, 400 MHz) of **7**.

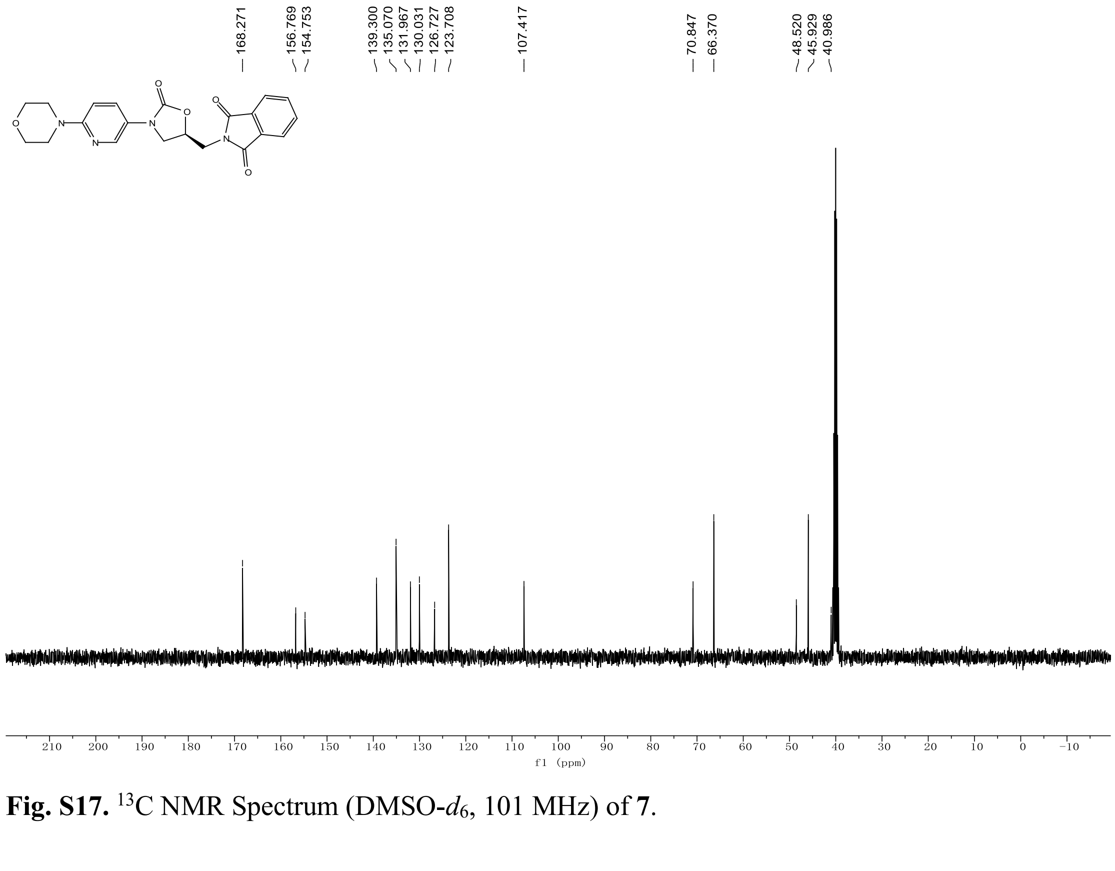

Fig. S17. ^{13}C NMR Spectrum (DMSO-d_6, 101 MHz) of **7**.

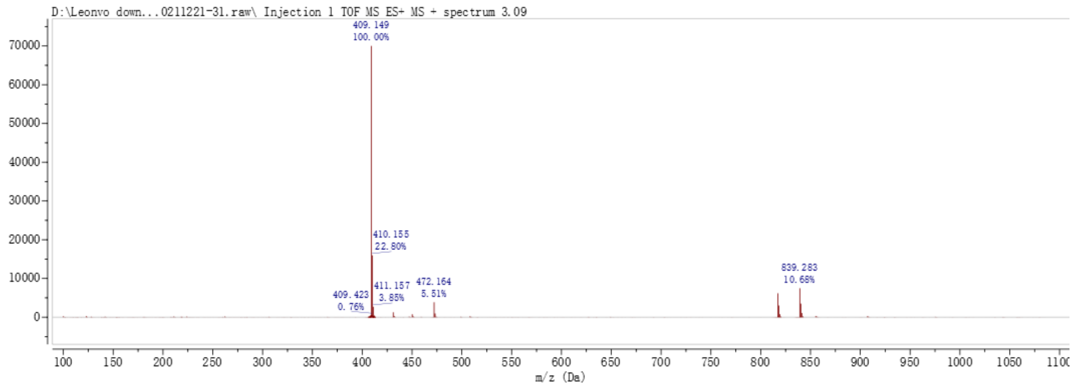

Fig. S18. MS calcd for $C_{21}H_{20}N_4O_5$ (Mwt.: 408.41): m/z 409.149 ([M+H] $^+$, bp) of **7**.

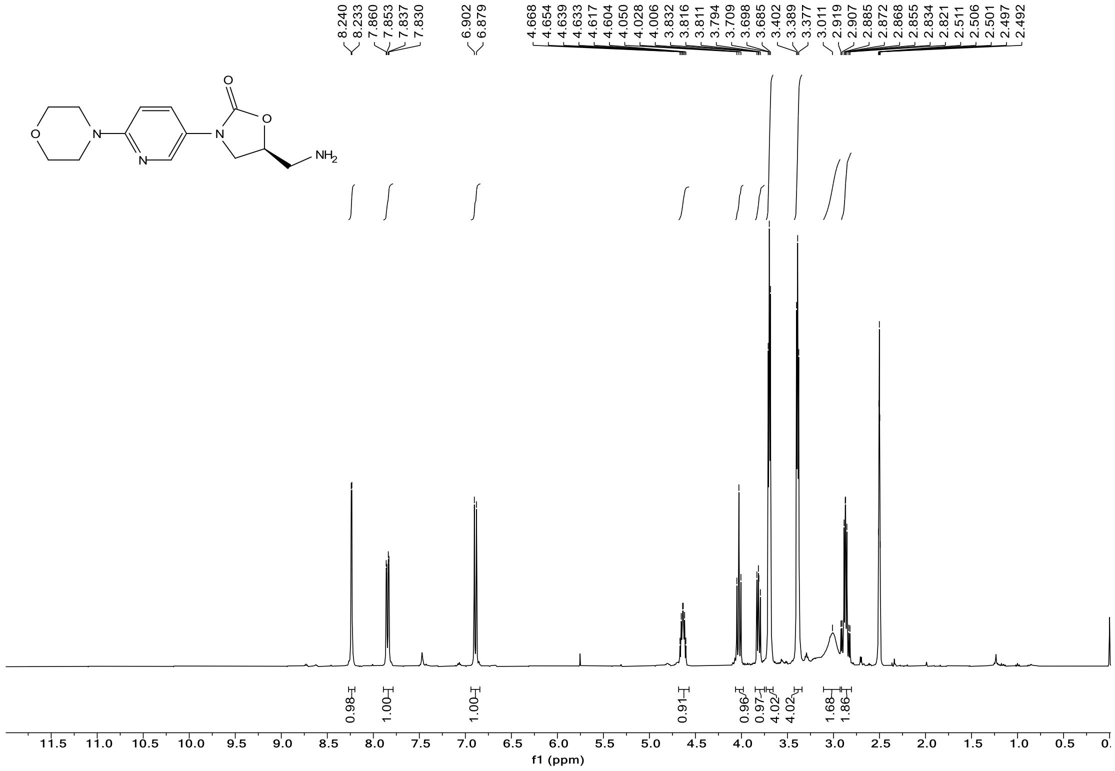

Fig. S19. ^{1}H NMR Spectrum (DMSO-d_6, 400 MHz) of **8**.

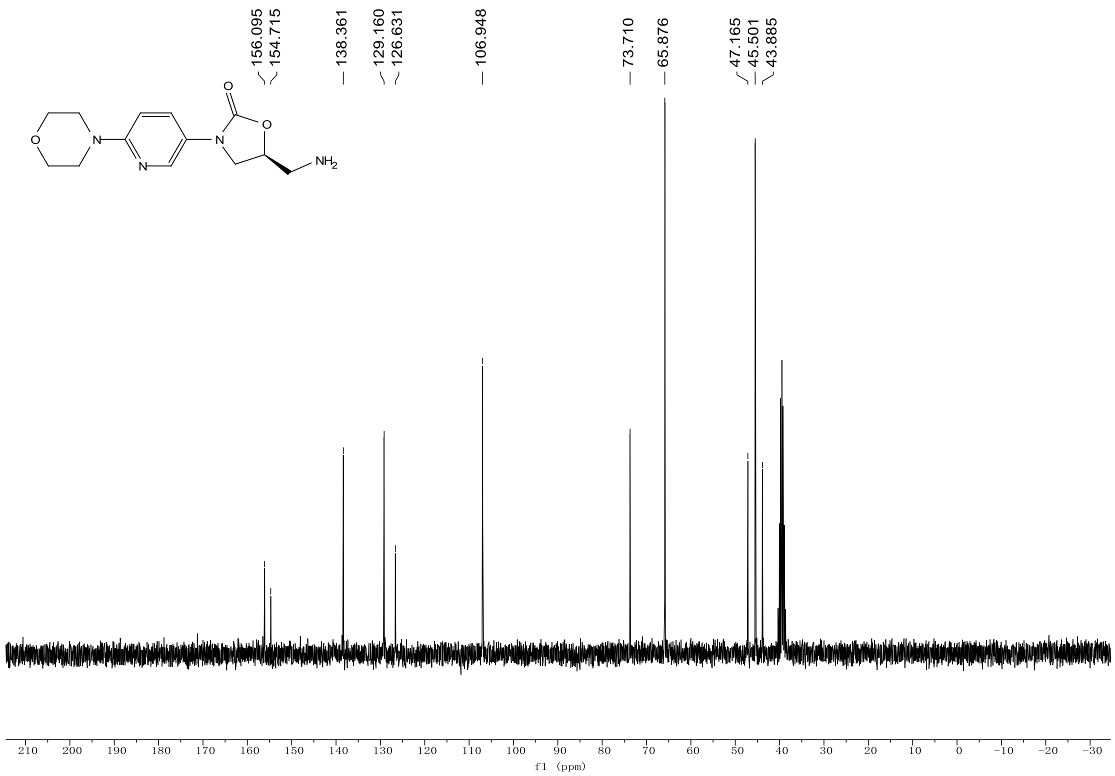

Fig. S20. ^{13}C NMR Spectrum (DMSO-d_6, 75 MHz) of **8**.

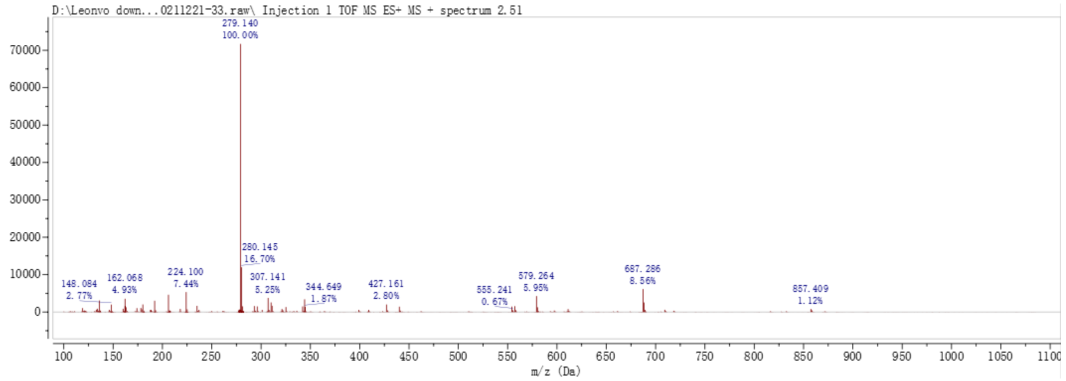

Fig. S21. MS calcd for $C_{13}H_{18}N_4O_3$ (Mwt.: 278.31): m/z 279.140 ([M+H] $^+$, bp) of **8**.

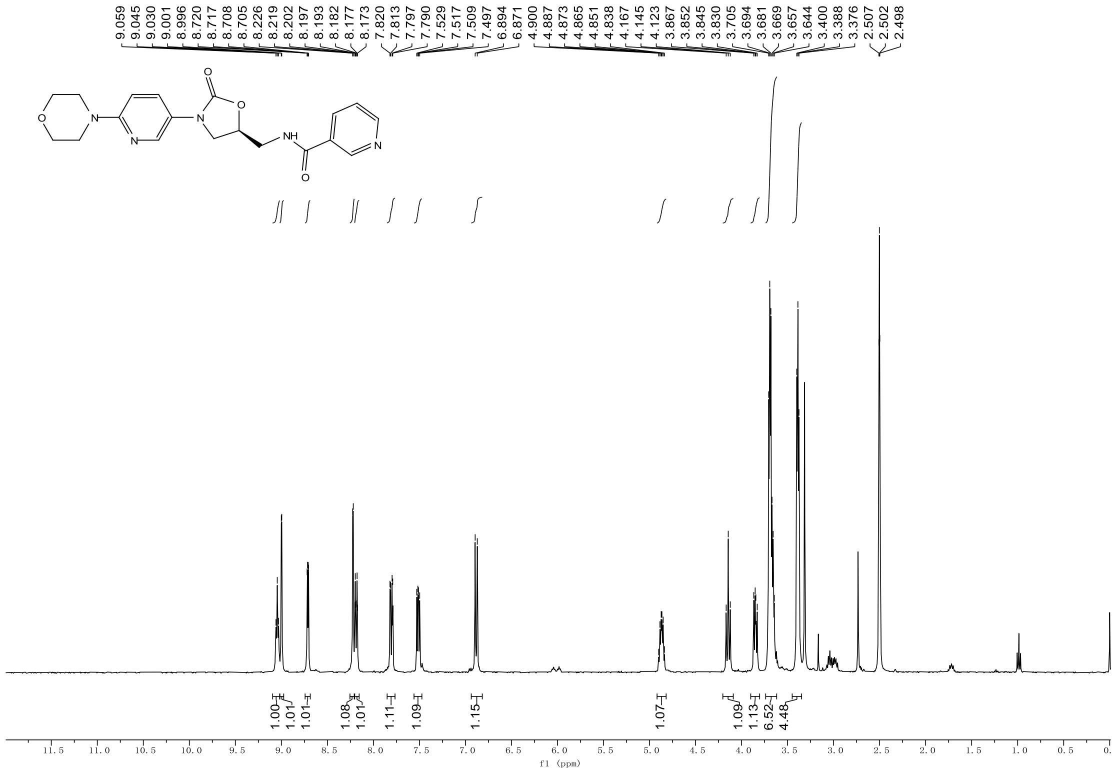

Fig. S22. ^{1}H NMR Spectrum (DMSO-d_6, 400 MHz) of **9a**.

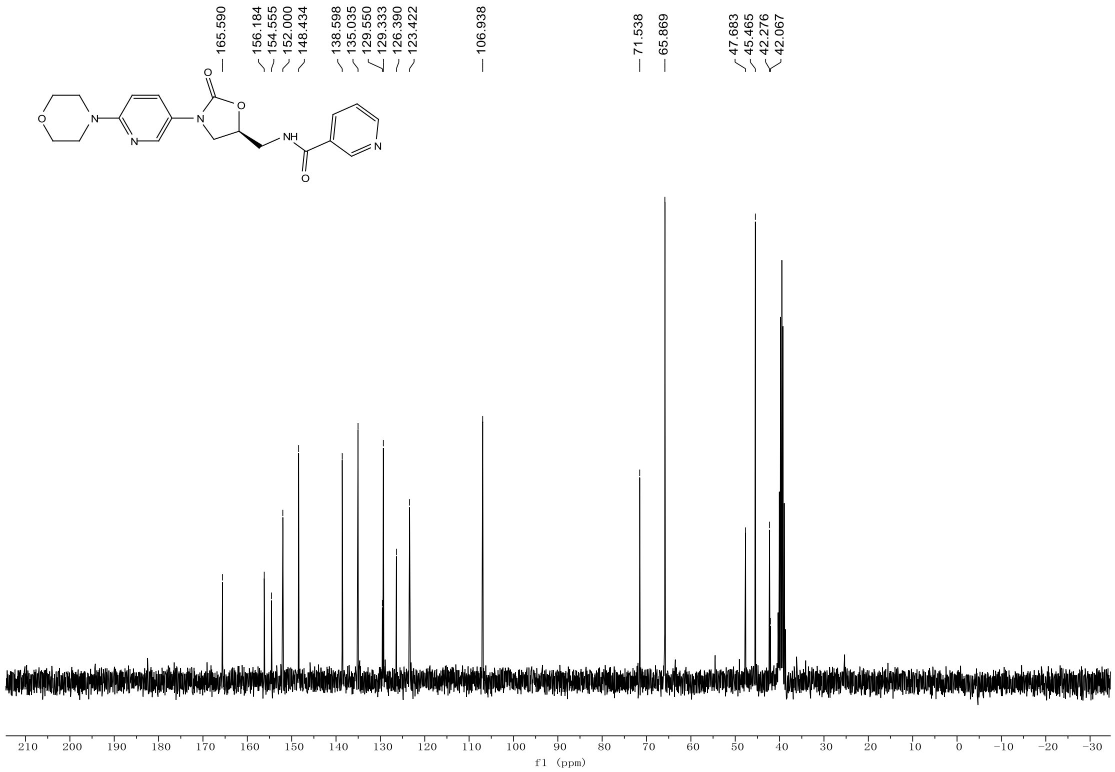

Fig. S23. ^{13}C NMR Spectrum (DMSO-d_6, 75 MHz) of **9a**.

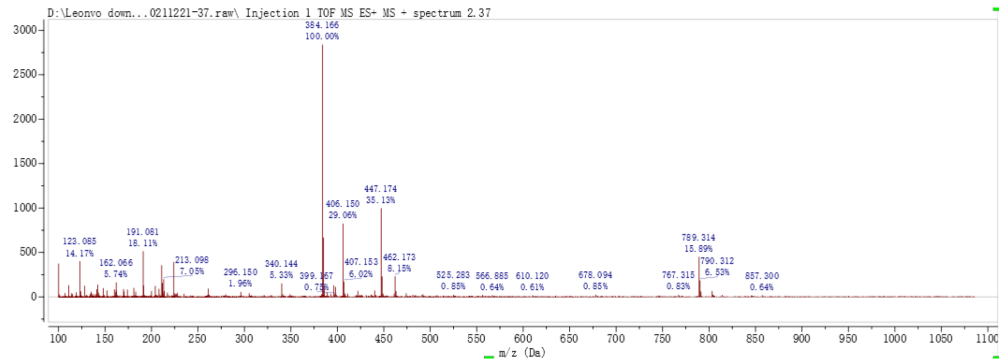

Fig. S24. MS calcd for $C_{19}H_{21}N_5O_4$ (Mwt.: 383.41): m/z 384.166 ([M+H] $^+$, bp) of **9a**.

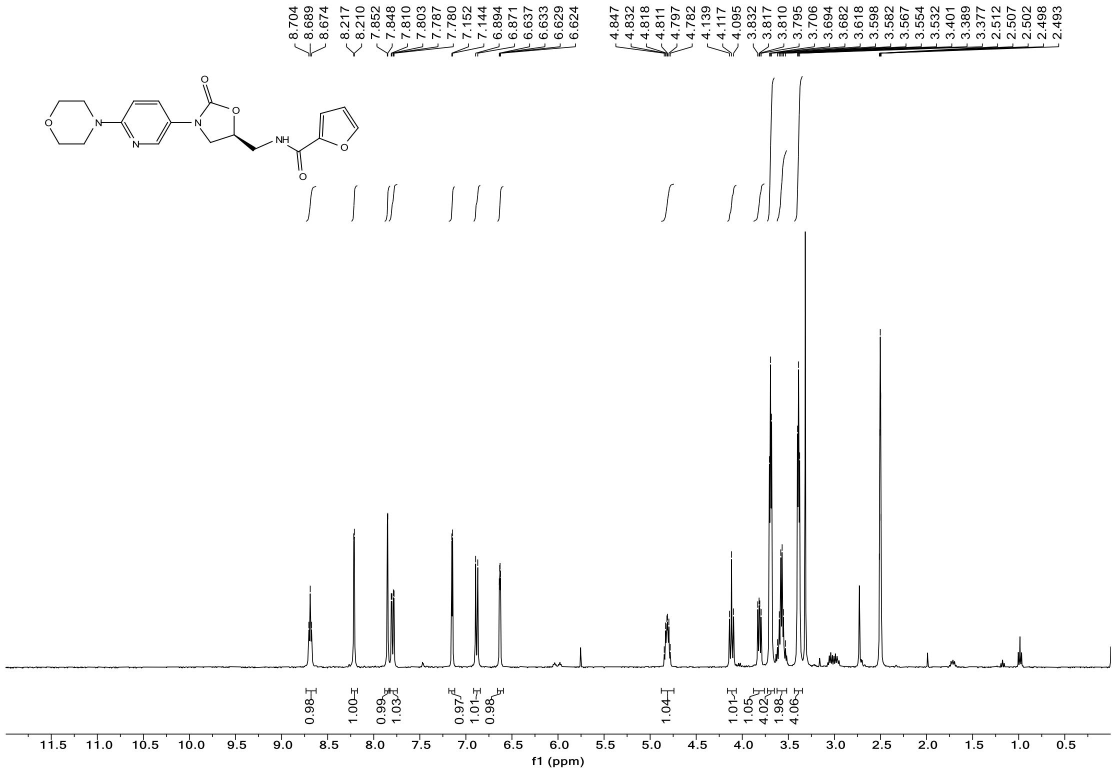

Fig. S25. ^{1}H NMR Spectrum (DMSO-d_6, 400 MHz) of **9b**.

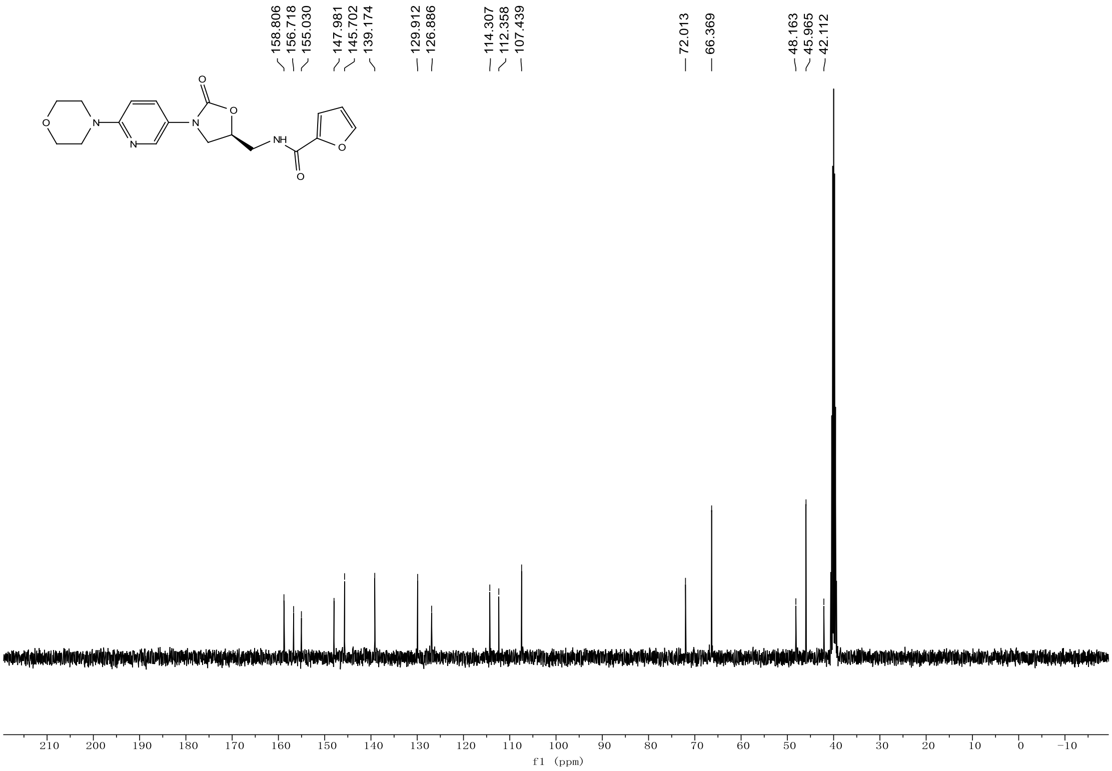

Fig. S26. ^{13}C NMR Spectrum (DMSO-d_6, 75 MHz) of **9b**.

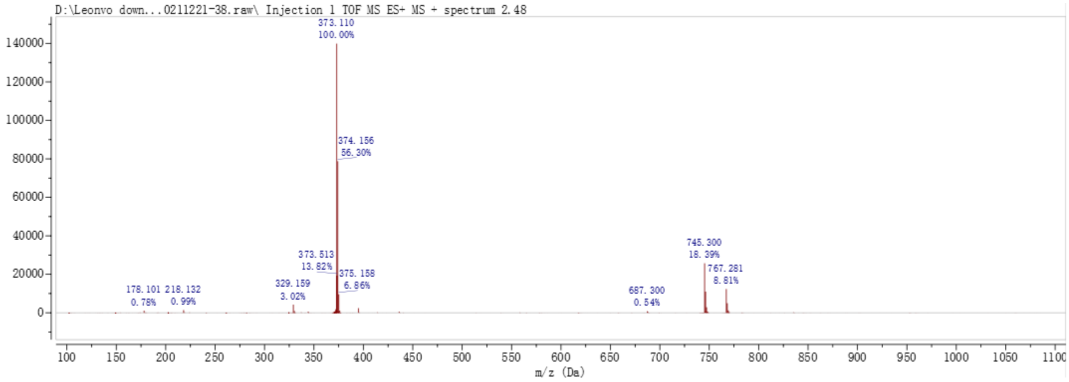

Fig. S27. MS calcd for $C_{18}H_{20}N_4O_5$ (Mwt.: 372.38): m/z 373.110 ([M+H] $^+$, bp) of **9b**.

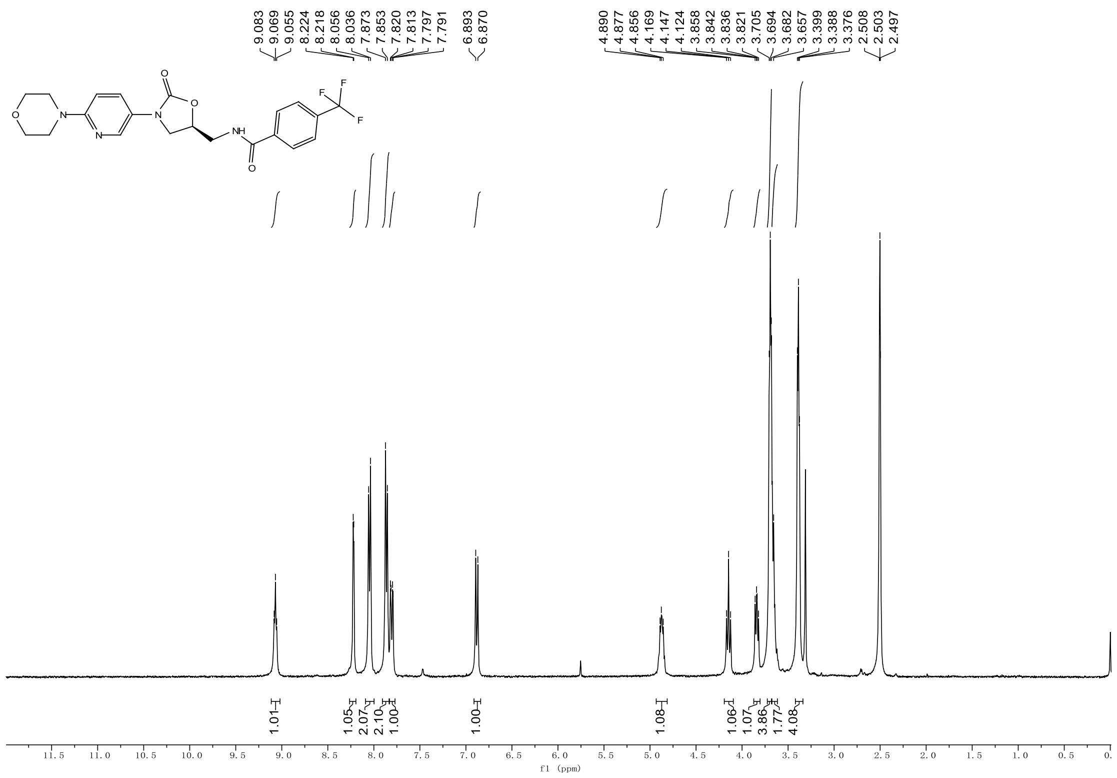

Fig. S28. ^{1}H NMR Spectrum (DMSO-d_6, 400 MHz) of **9c**.

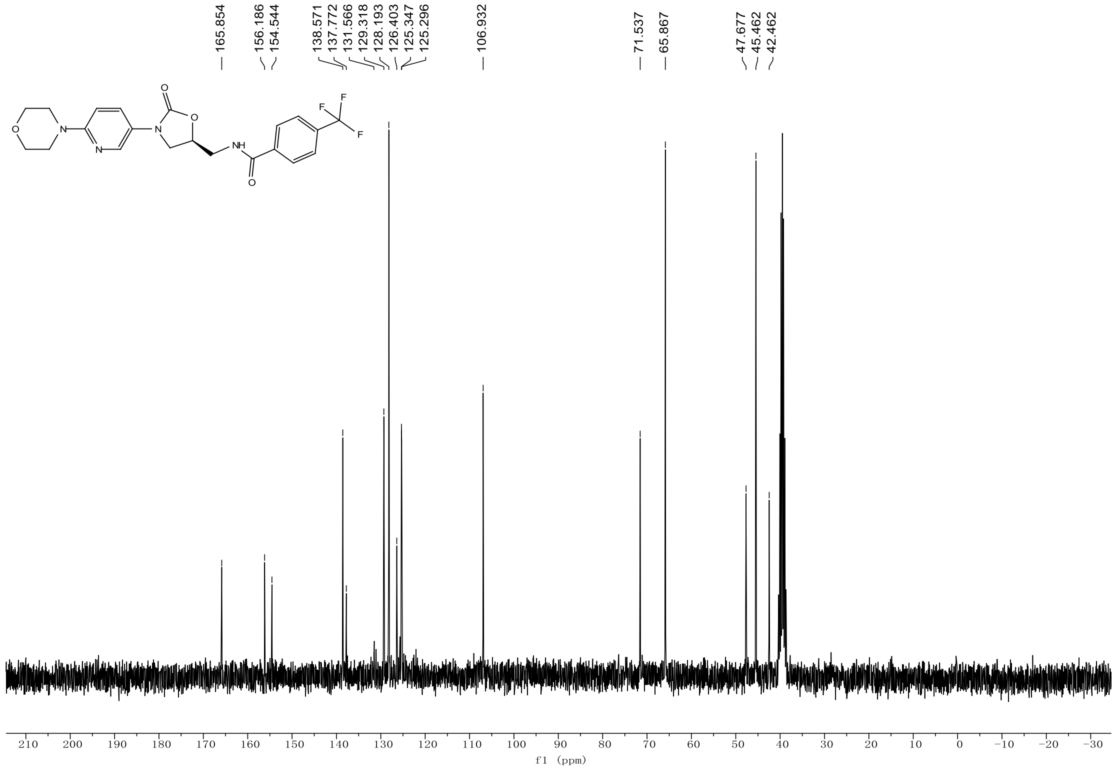

Fig. S29. ^{13}C NMR Spectrum (DMSO-d_6, 75 MHz) of **9c**.

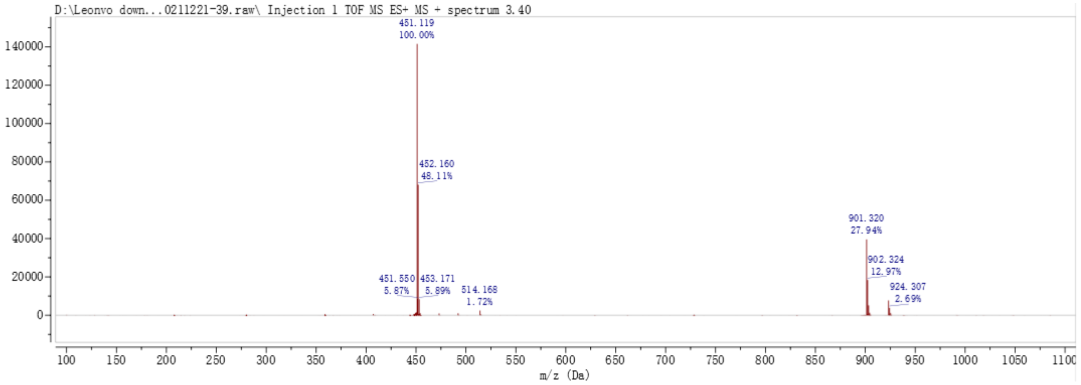

Fig. S30. MS calcd for $C_{21}H_{21}F_{3}N_{4}O_{4}$ (Mwt.: 450.42): m/z 451.119 ([M+H] $^{+}$, bp) of **9c**.

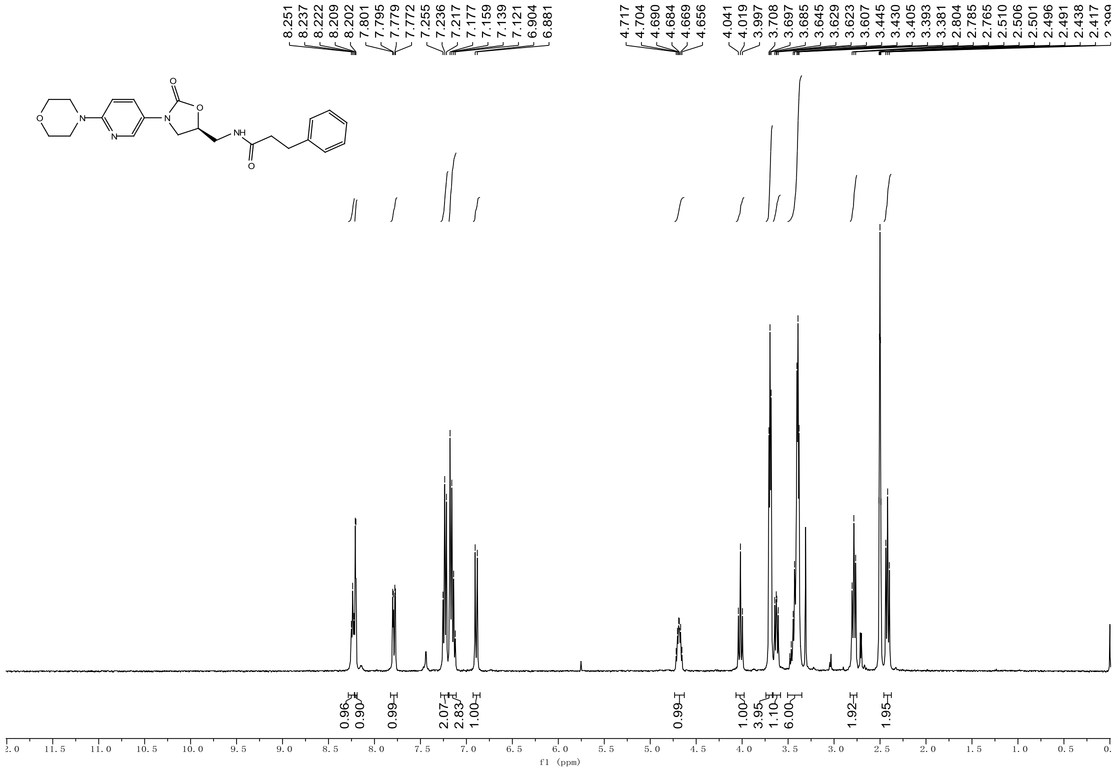

Fig. S31. ^{1}H NMR Spectrum (DMSO-d_6, 400 MHz) of **9d**.

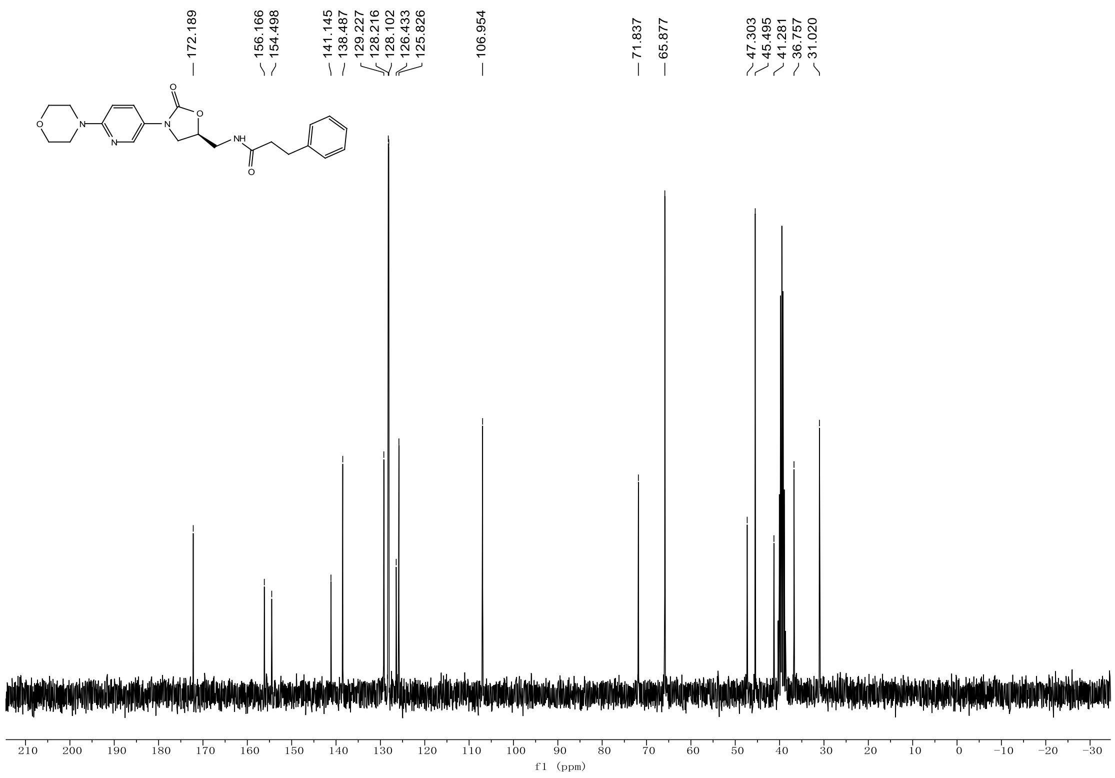

Fig. S32. ^{13}C NMR Spectrum (DMSO-d_6, 75 MHz) of **9d**.

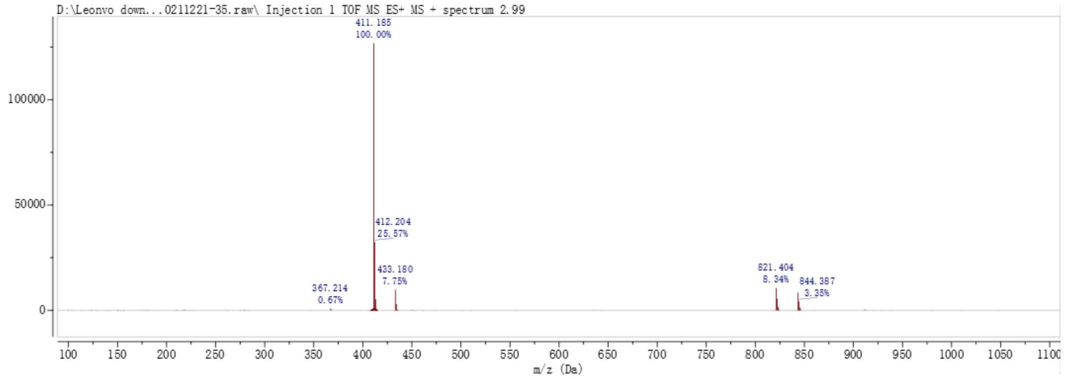

Fig. S33. MS calcd for $C_{22}H_{26}N_4O_4$ (Mwt.: 410.47): m/z 411.185 ([M+H] $^+$, bp) of **9d**.

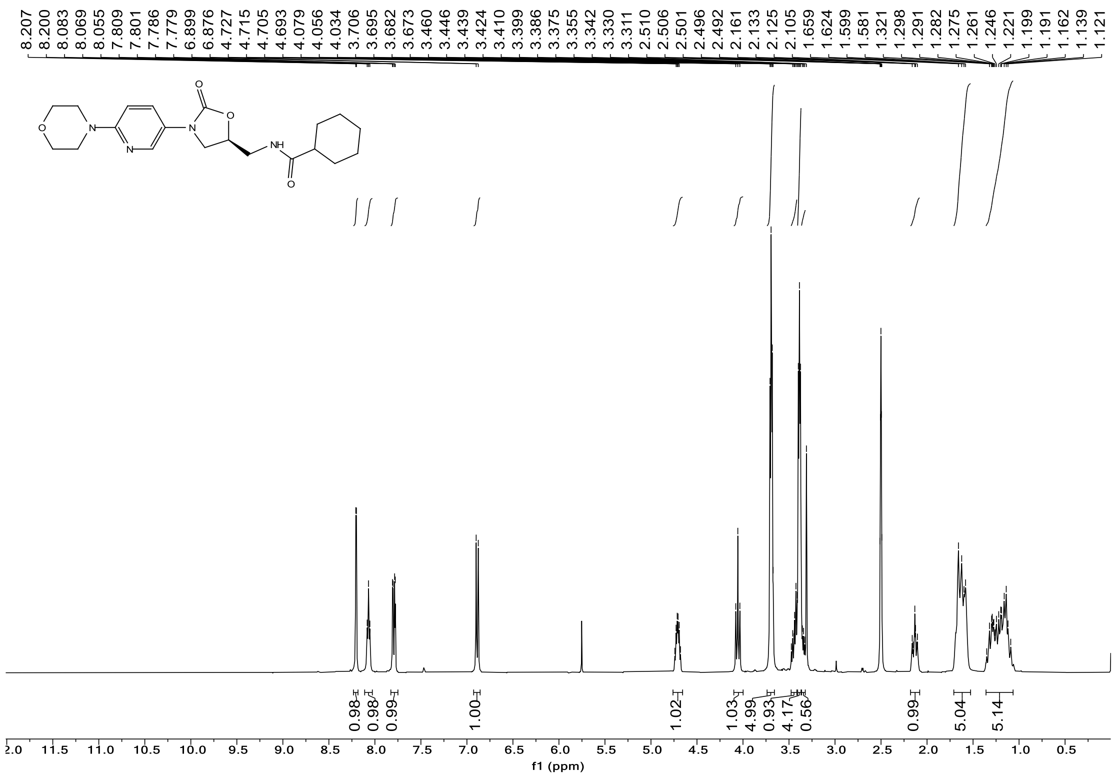

Fig. S34. ^{1}H NMR Spectrum (DMSO-d_6, 400 MHz) of **9e**.

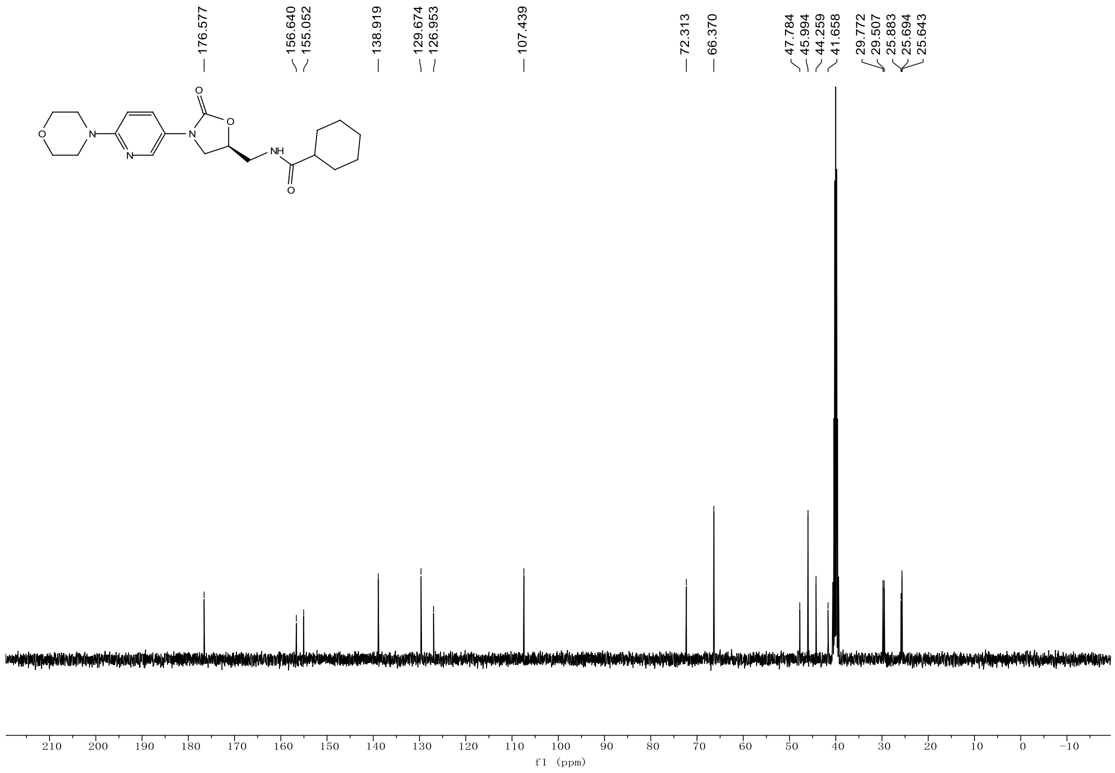

Fig. S35. ^{13}C NMR Spectrum (DMSO-d_6, 75 MHz) of **9e**.

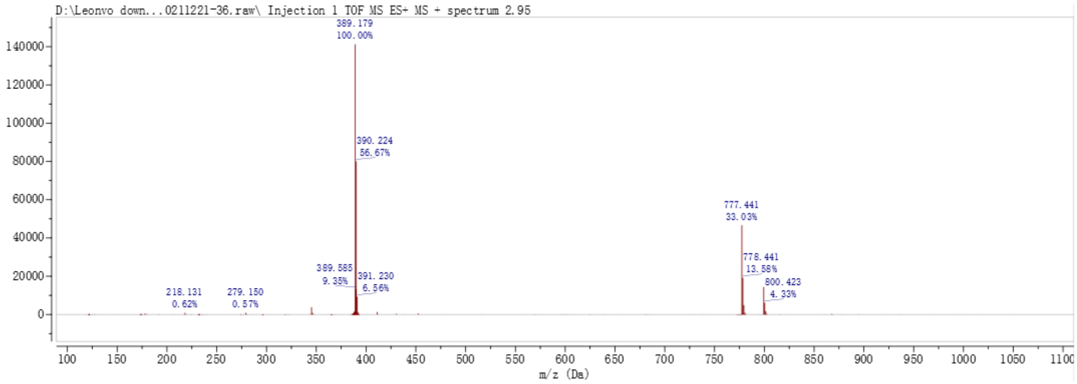

Fig. S36. MS calcd for $C_{20}H_{28}N_4O_4$ (Mwt.: 388.47): m/z 389.179 ([M+H] $^+$, bp) of **9e**.

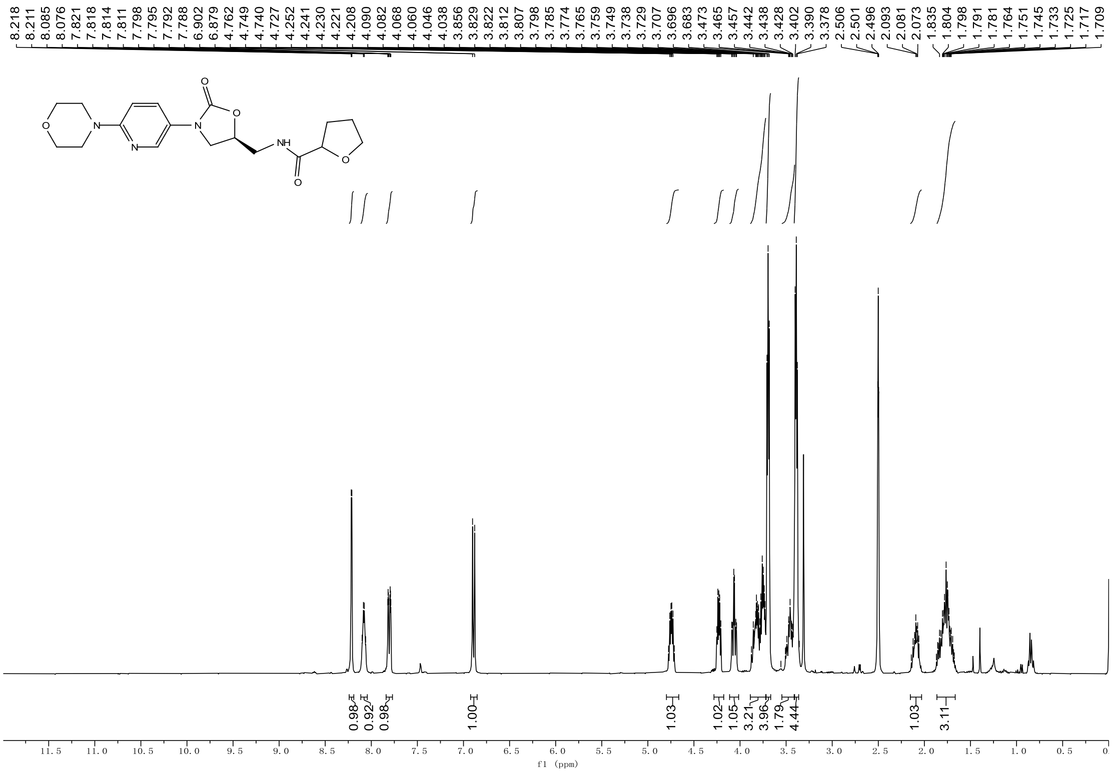

Fig. S37. ^{1}H NMR Spectrum (DMSO-d_6, 400 MHz) of **9f**.

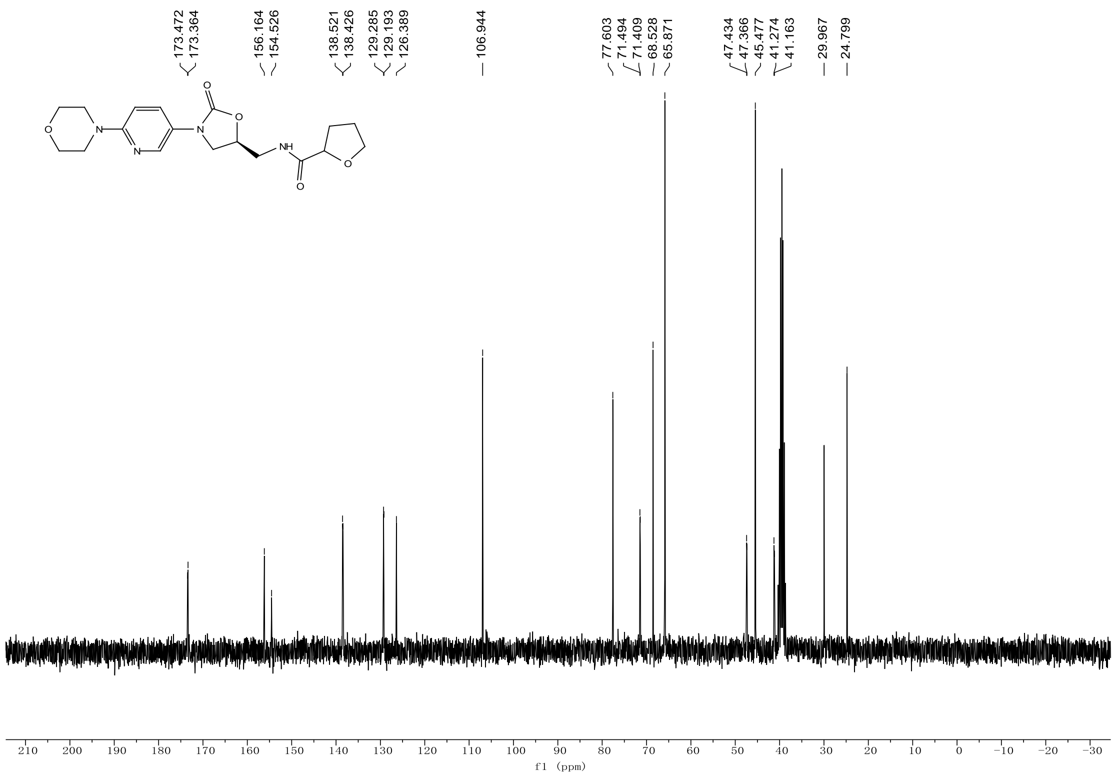

Fig. S38. ^{13}C NMR Spectrum (DMSO-d_6, 75 MHz) of **9f**.

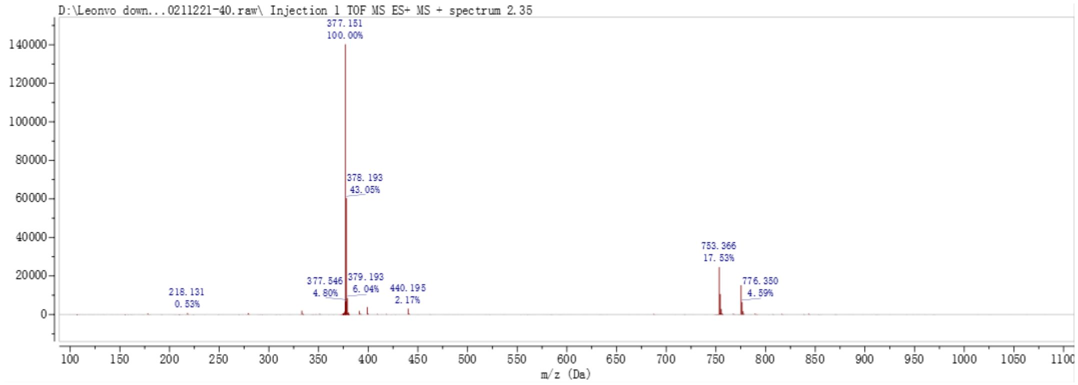

Fig. S39. MS calcd for $C_{18}H_{24}N_4O_5$ (Mwt.: 476.41): m/z 377.151 ([M+H] $^+$, bp) of **9f**.

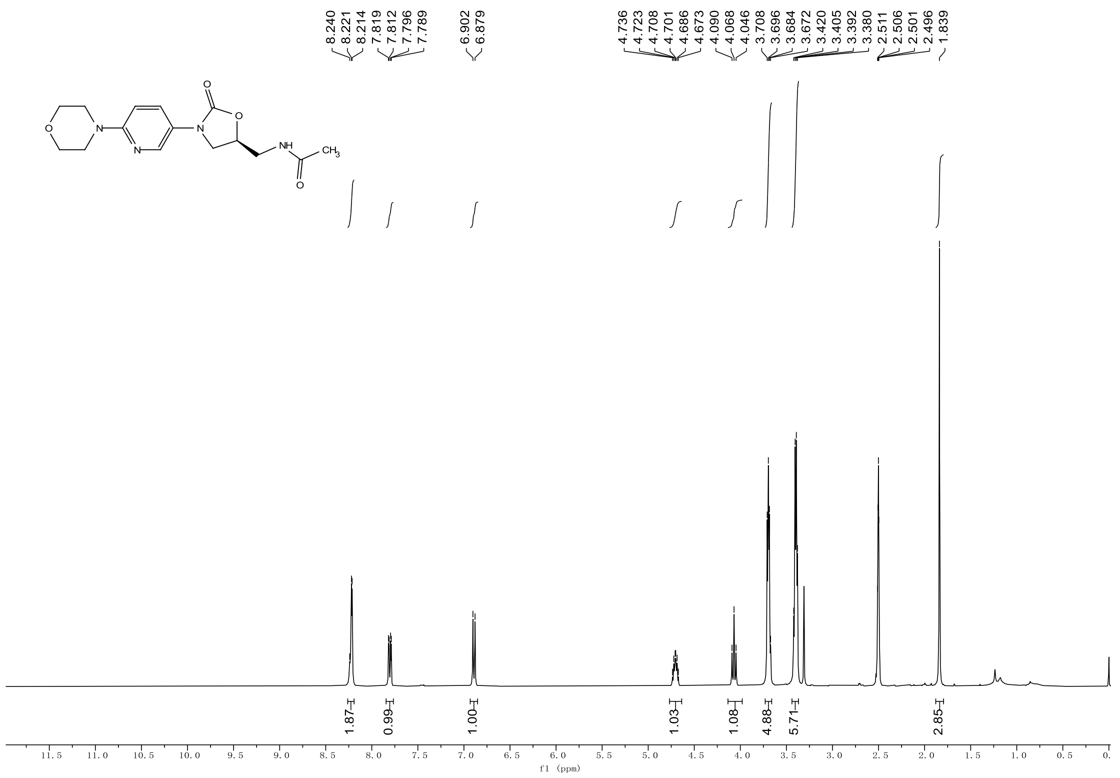

Fig. S40. ^{1}H NMR Spectrum (DMSO-d_6, 400 MHz) of **9g**.

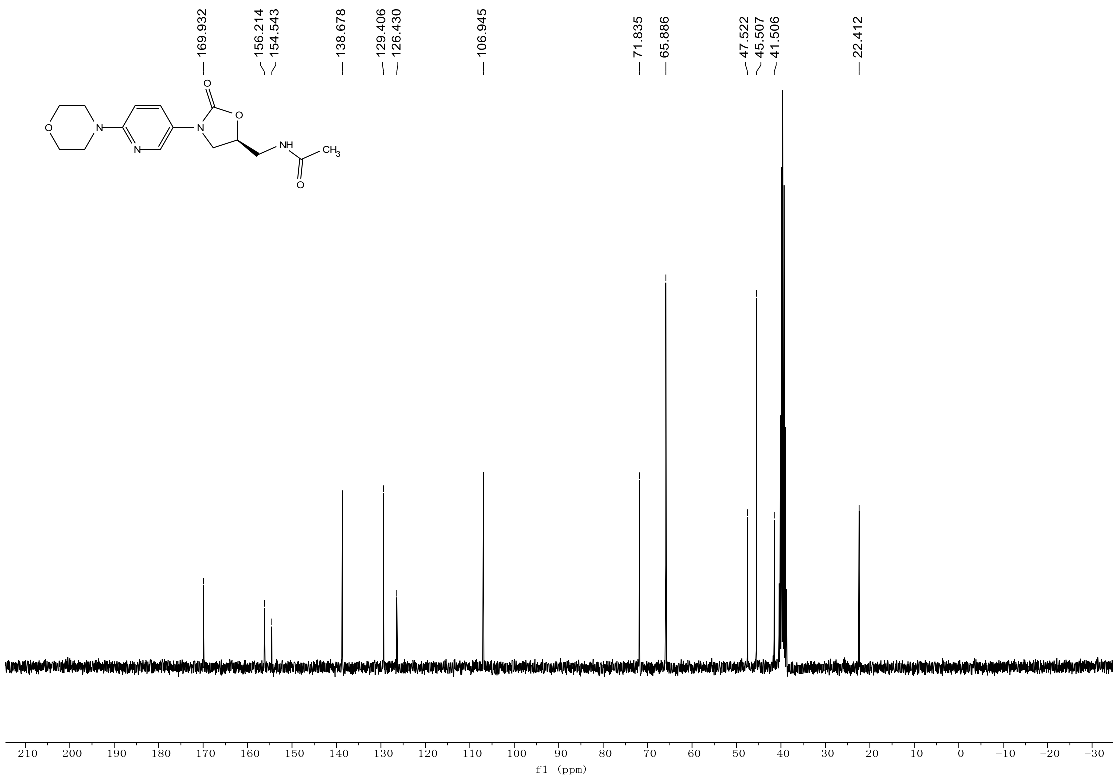

Fig. S41. ^{13}C NMR Spectrum (DMSO-d_6, 75 MHz) of **9g**.

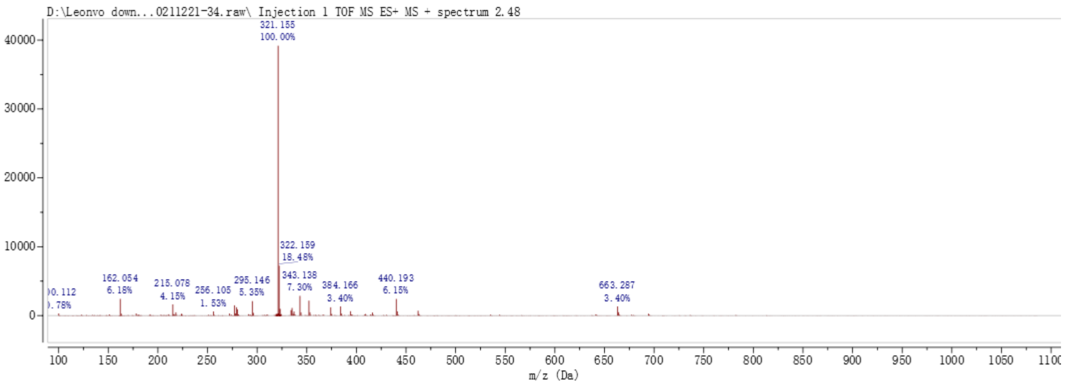

Fig. S42. MS calcd for $C_{15}H_{20}N_4O_4$ (Mwt.: 320.35): m/z 321.155 ([M+H] $^+$, bp) of **9g**.

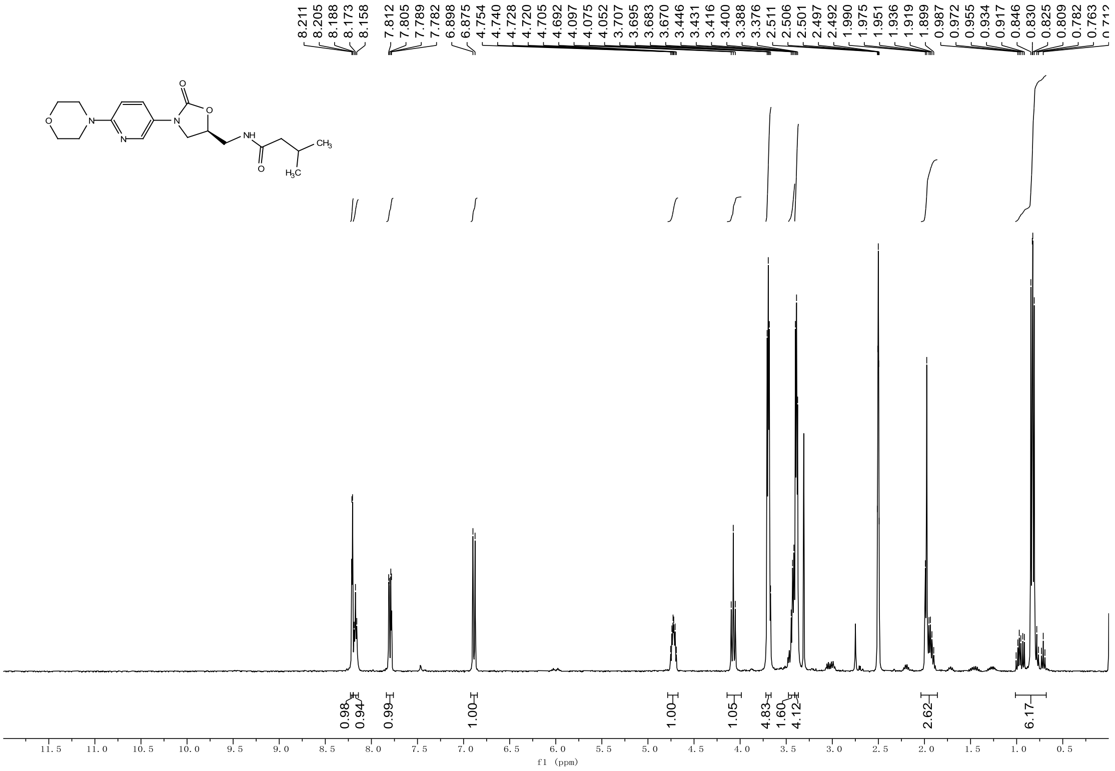

Fig. S43. ^{1}H NMR Spectrum (DMSO-d_6, 400 MHz) of **9h**.

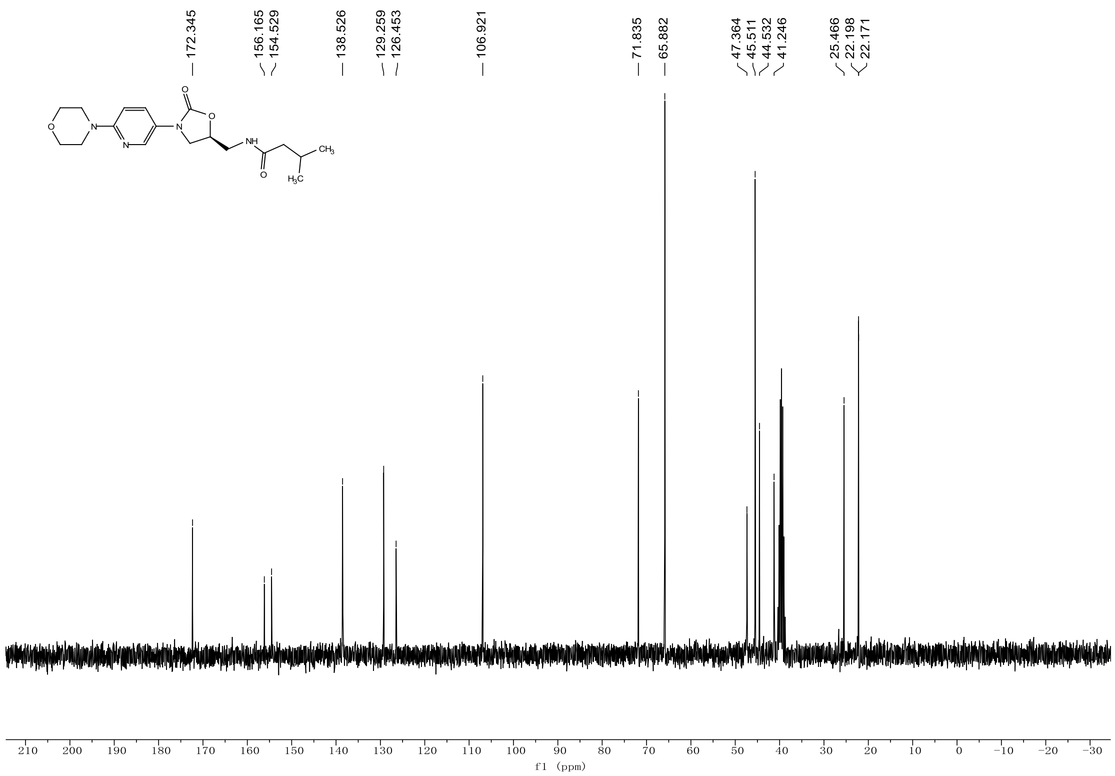

Fig. S44. ^{13}C NMR Spectrum (DMSO-d_6, 75 MHz) of **9h**.

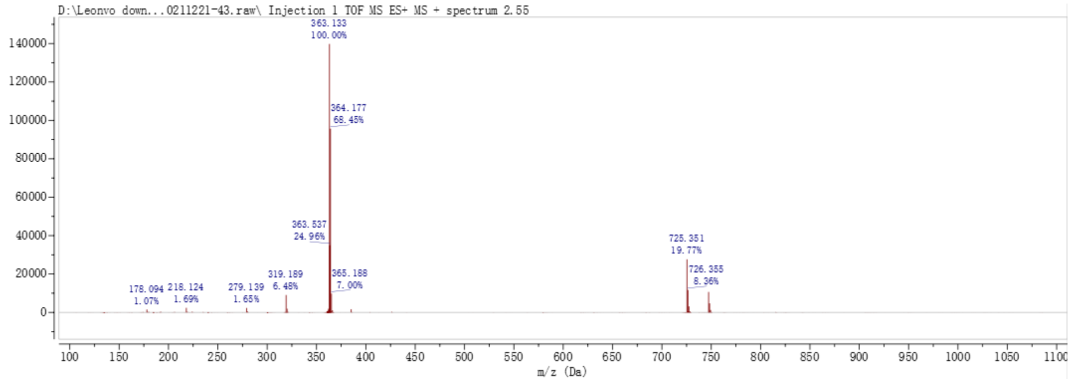

Fig. S45. MS calcd for $C_{18}H_{26}N_4O_4$ (Mwt.: 362.43): m/z 363.133 ([M+H] $^+$, bp) of **9h**.

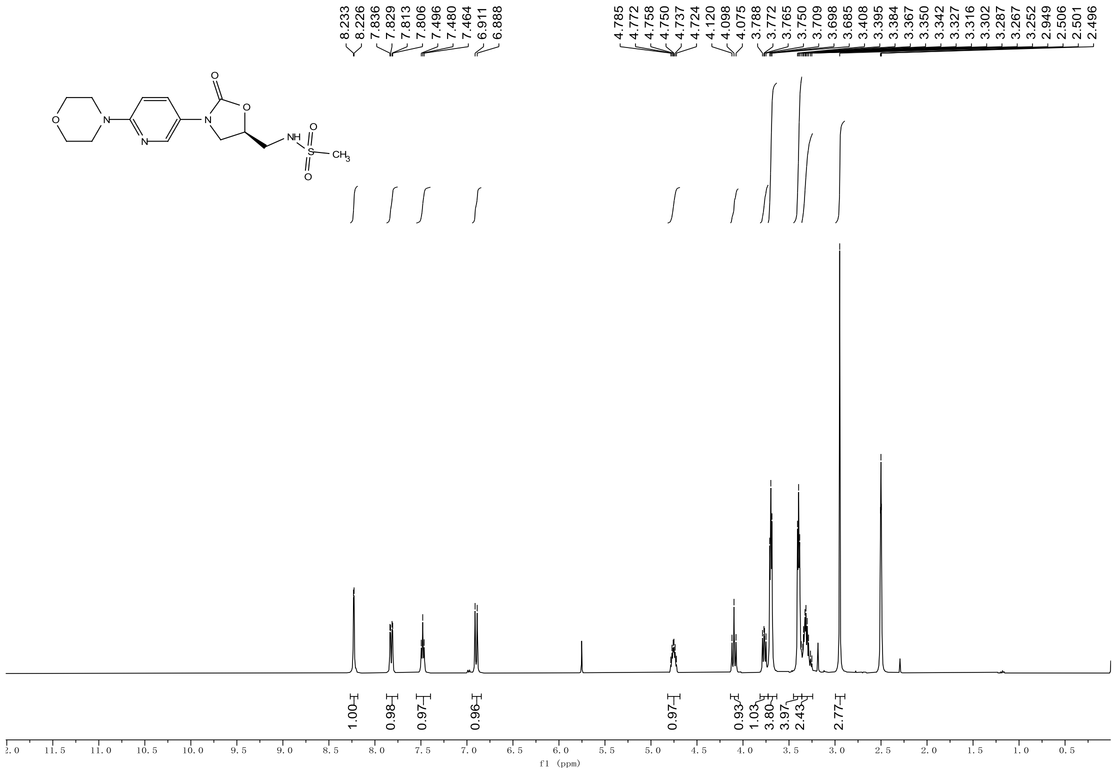

Fig. S46. ^{1}H NMR Spectrum (DMSO-d_6, 400 MHz) of **9i**.

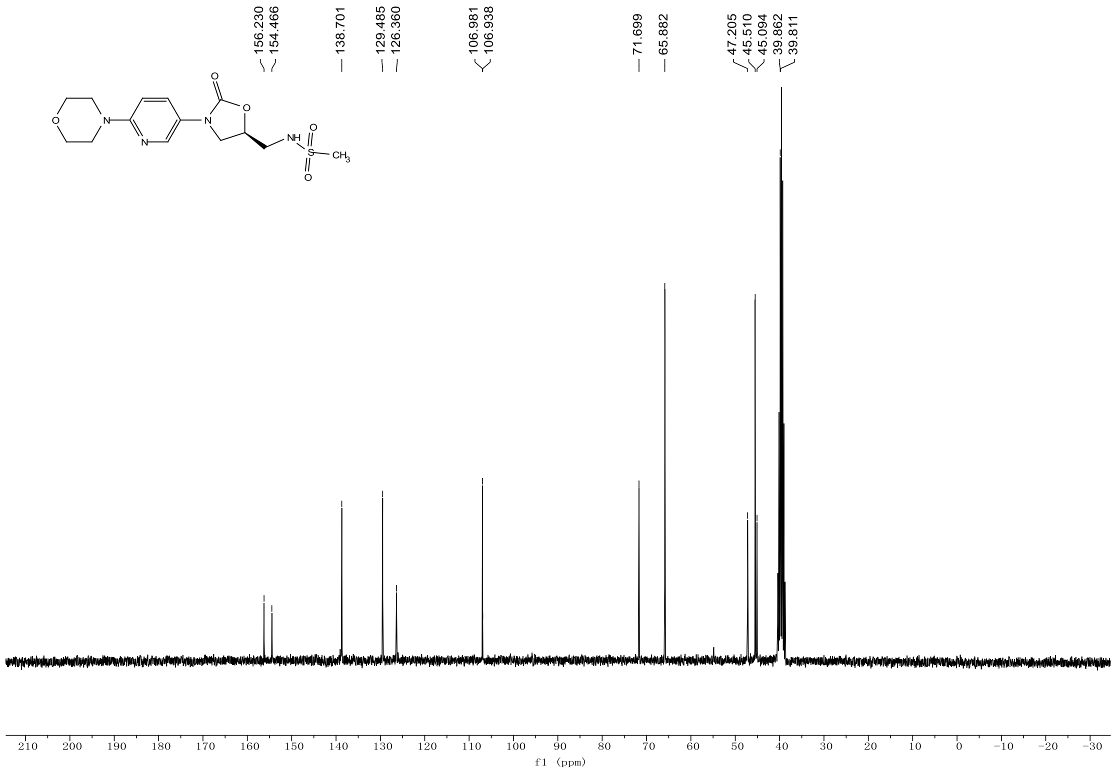

Fig. S47. ^{13}C NMR Spectrum (DMSO-d_6, 75 MHz) of **9i**.

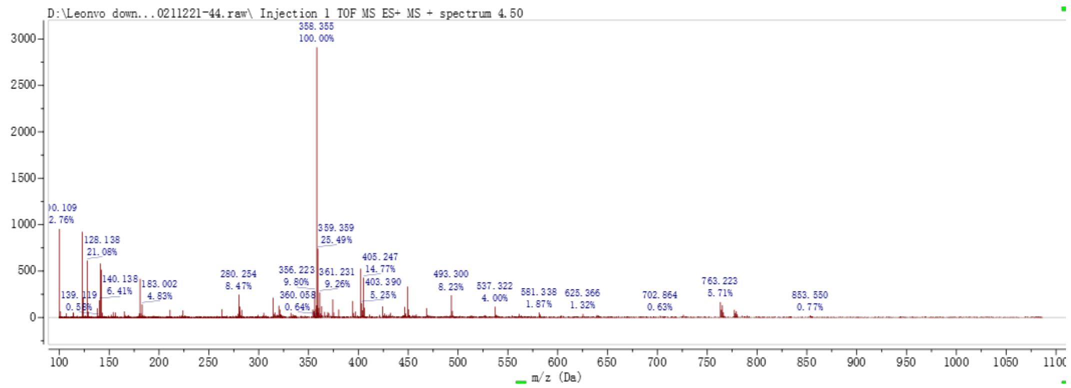

Fig. S48. MS calcd for $C_{14}H_{20}N_4O_5S$ (Mwt.: 356.40): m/z 358.335 ([M+H] $^{+}$, bp) of **9i**.

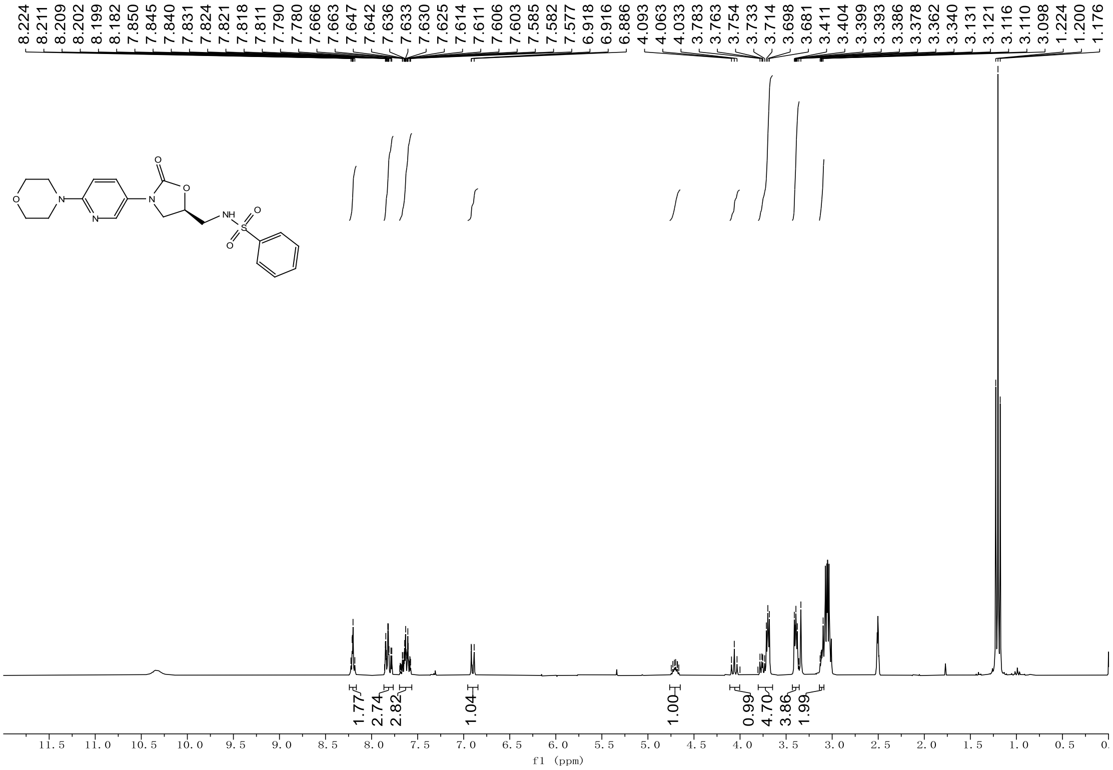

Fig. S49. ^{1}H NMR Spectrum (DMSO-d_6, 400 MHz) of **9j**.

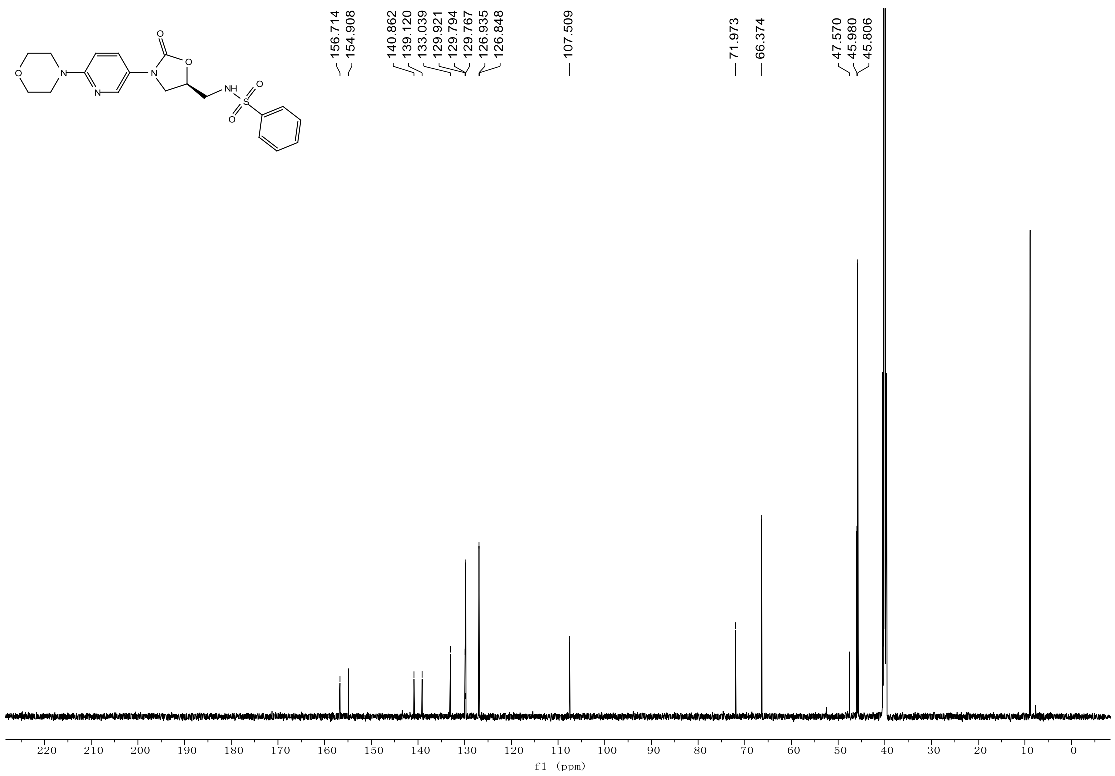

Fig. S50. ^{13}C NMR Spectrum (DMSO-d_6, 75 MHz) of **9j**.

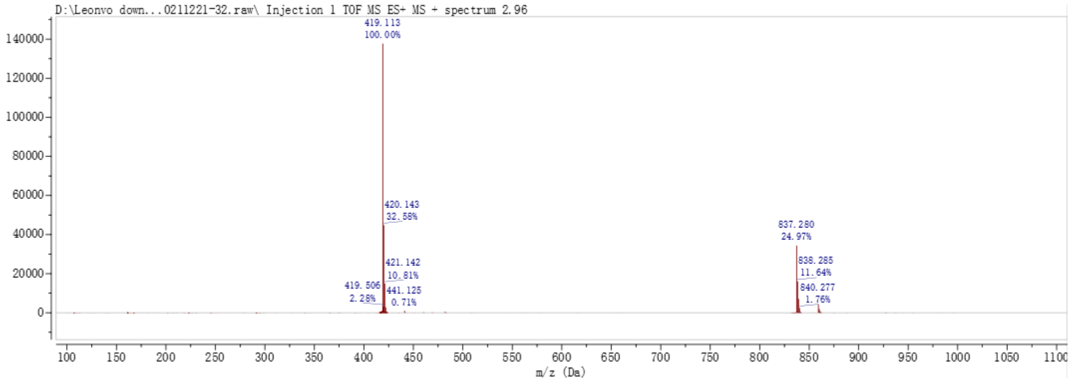

Fig. S51. MS calcd for $C_{19}H_{22}N_4O_5S$ (Mwt.: 418.47): m/z 419.133 ($[M+H]^+$, bp) of **9j**.

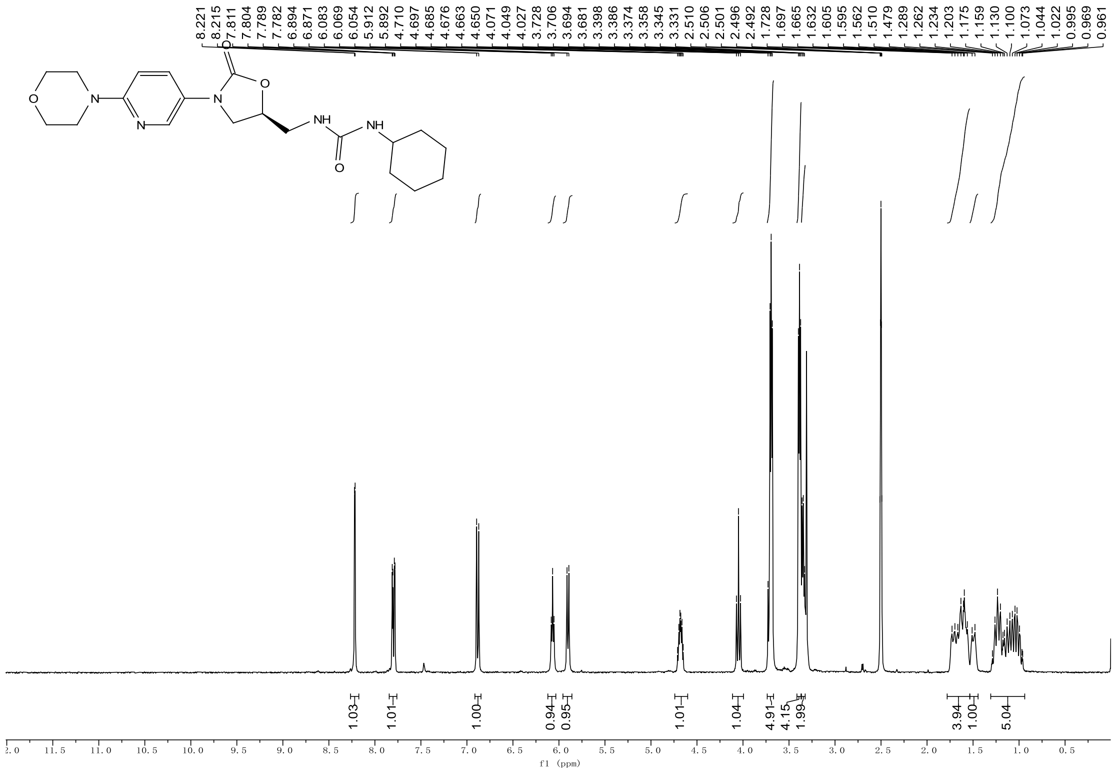

Fig. S52. ^{1}H NMR Spectrum (DMSO-d_6, 400 MHz) of **9k**.

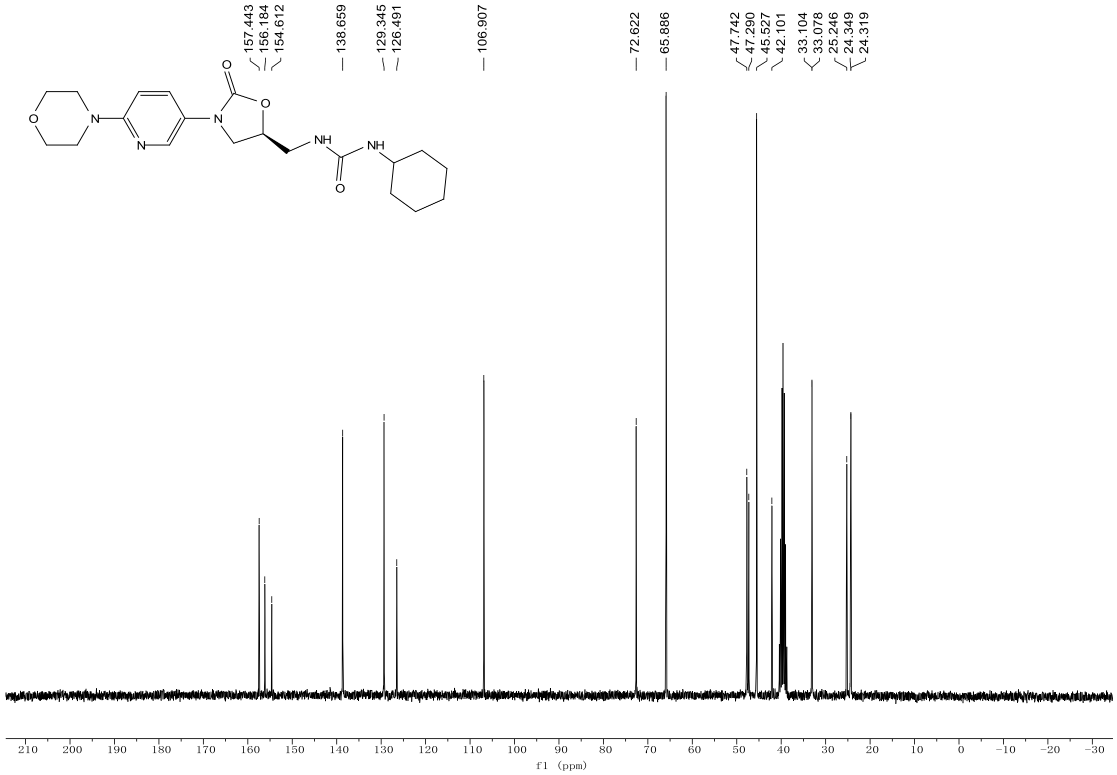

Fig. S53. ^{13}C NMR Spectrum (DMSO-d_6, 75 MHz) of **9k**.

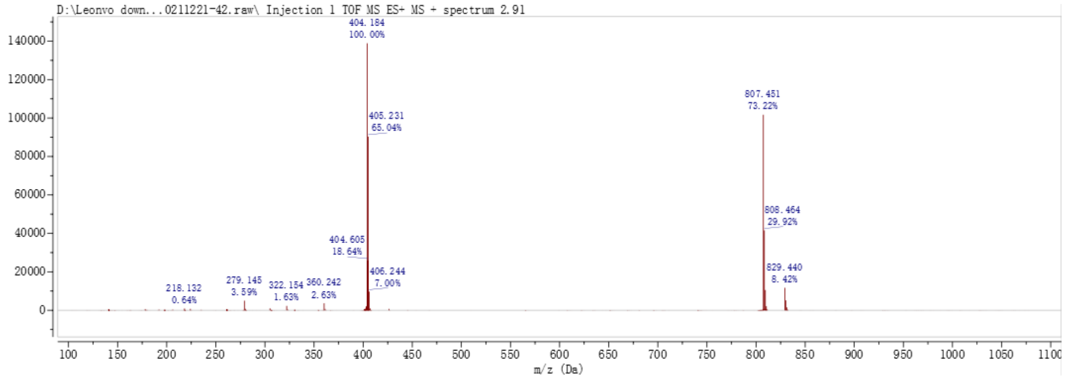

Fig. S54. MS calcd for $C_{20}H_{29}N_5O_4$ (Mwt.: 403.48): m/z 404.184 ([M+H] $^+$, bp) of **9k**.

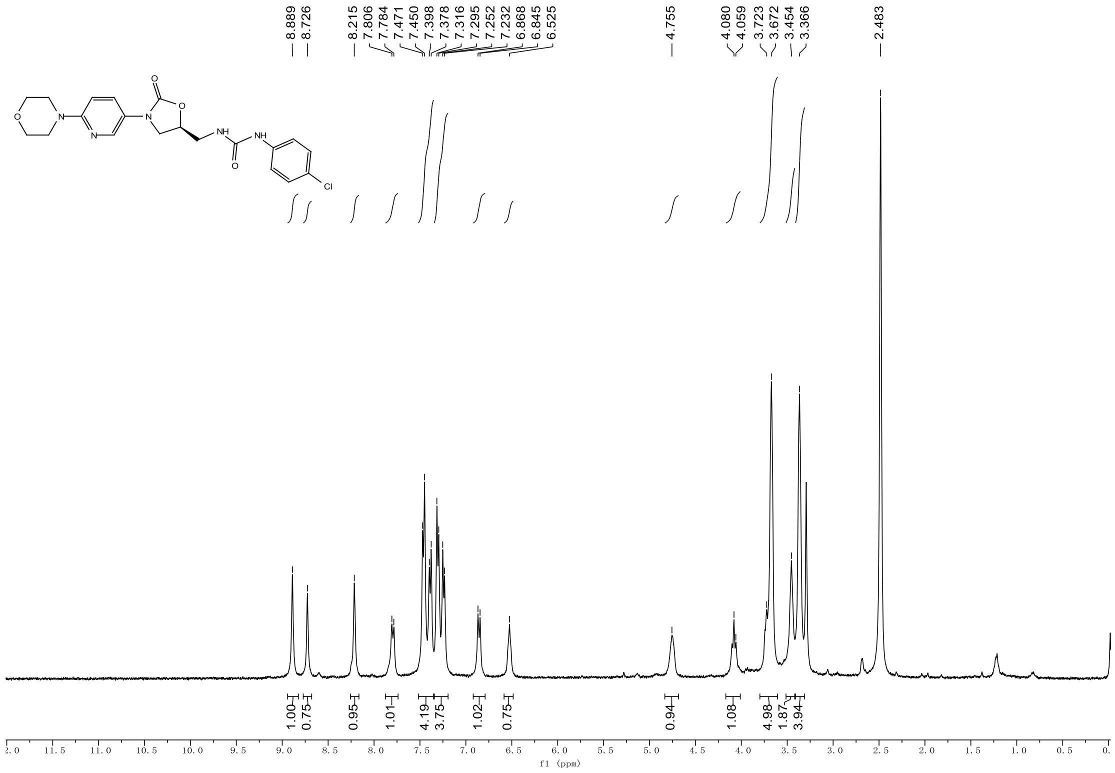

Fig. S55. ^{1}H NMR Spectrum (DMSO-d_6, 400 MHz) of **9l**.

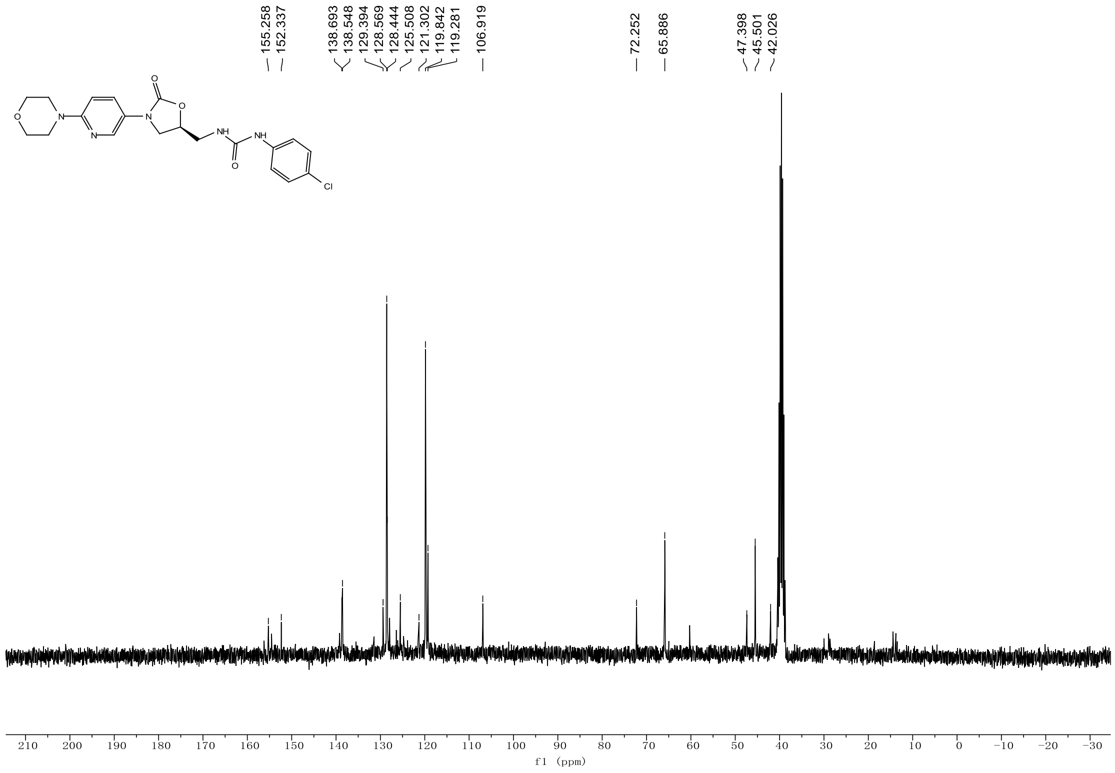

Fig. S56. ^{13}C NMR Spectrum (DMSO-d_6, 75 MHz) of **9l**.

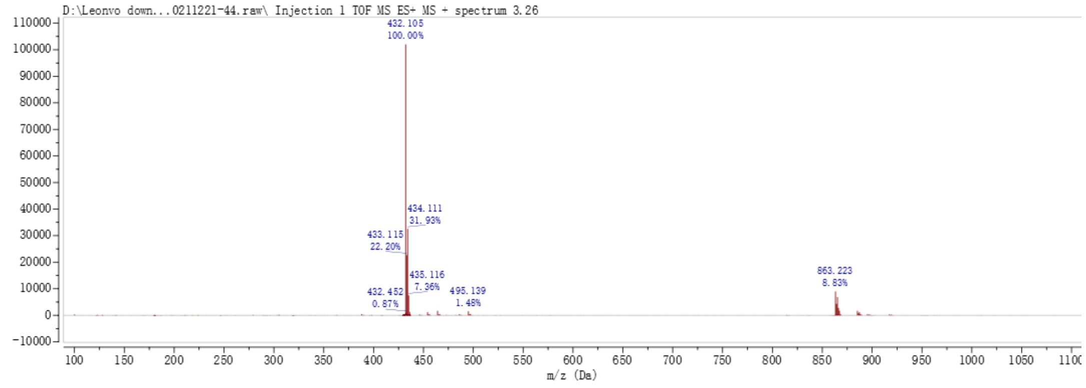

Fig. S57. MS calcd for $C_{20}H_{22}ClN_5O_4$ (Mwt.: 431.88): m/z 432.105 ([M+H] $^+$, bp) of **9l**.

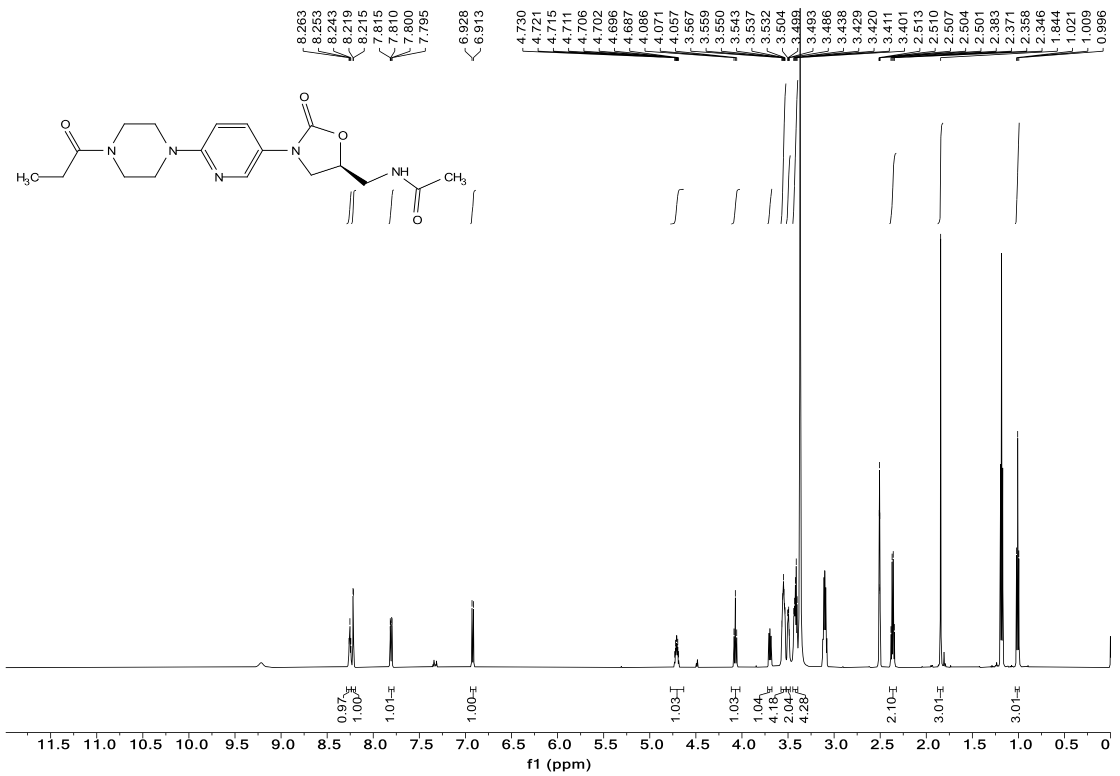

Fig. S58. ^{1}H NMR Spectrum (DMSO-d_6, 400 MHz) of **17a**.

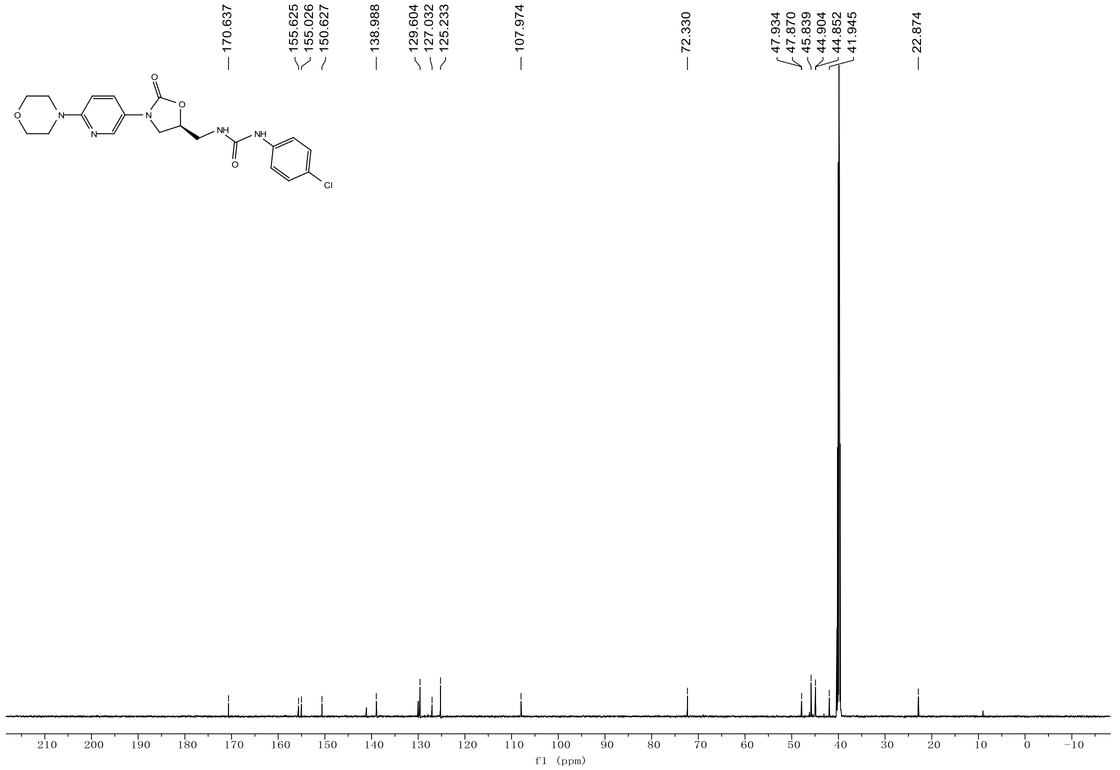

Fig. S59. ^{13}C NMR Spectrum (DMSO-d_6, 75 MHz) of **17a**.

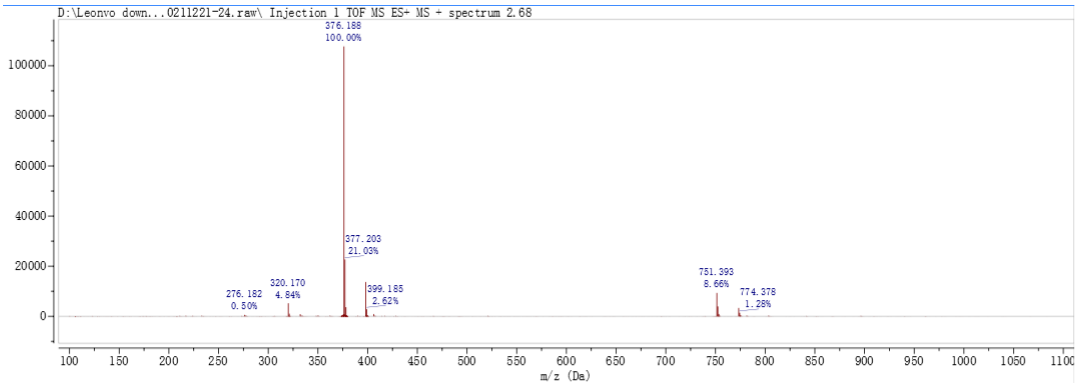

Fig. S60. MS calcd for $C_{18}H_{25}N_5O_4$ (Mwt.: 375.43): m/z 376.188 ([M+H] $^+$, bp) of **17a**.

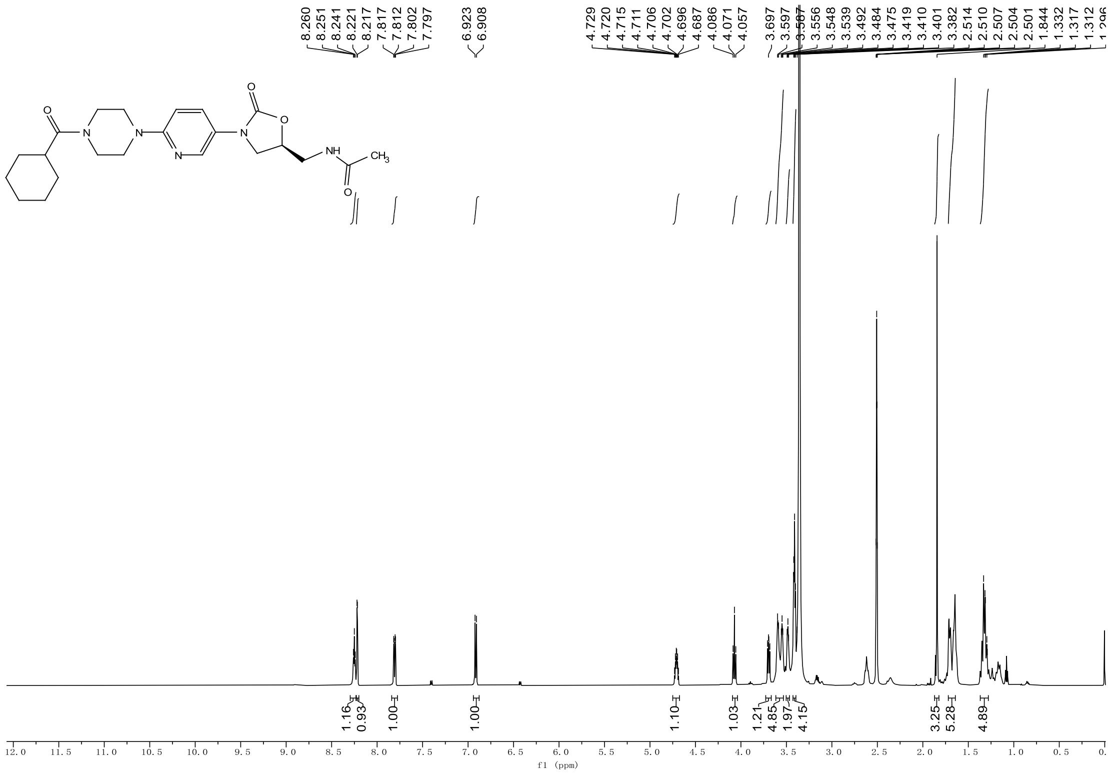

Fig. S61. ^1H NMR Spectrum (DMSO-d_6, 400 MHz) of **17b**.

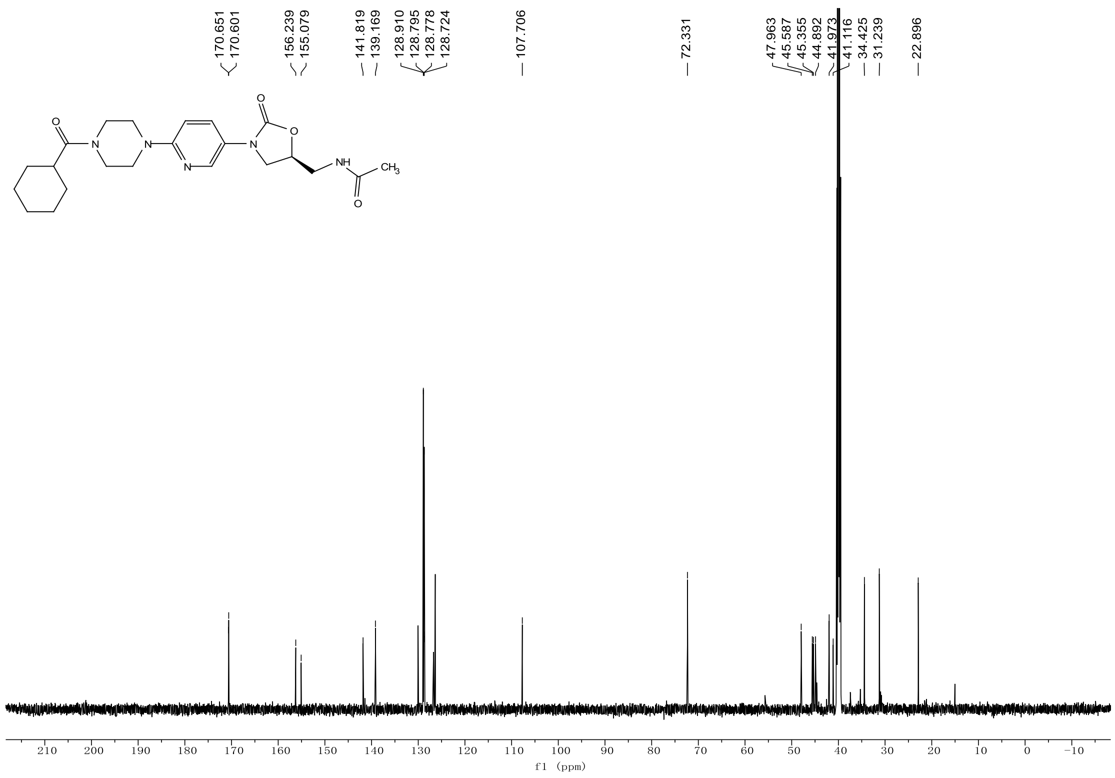

Fig. S62. ^{13}C NMR Spectrum (DMSO-d_6, 75 MHz) of **17b**.

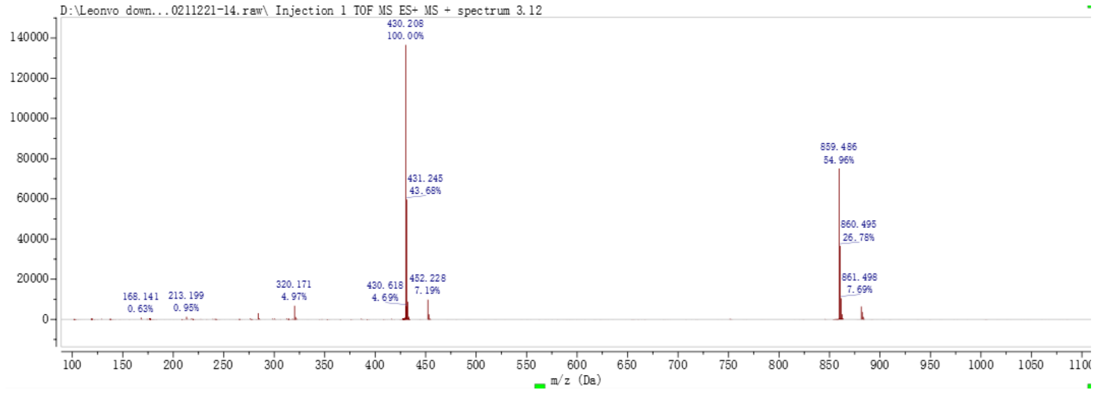

Fig. S63. MS calcd for $C_{22}H_{31}N_5O_4$ (Mwt.: 429.52): m/z 430.208 ([M+H] $^+$, bp) of **17b**.

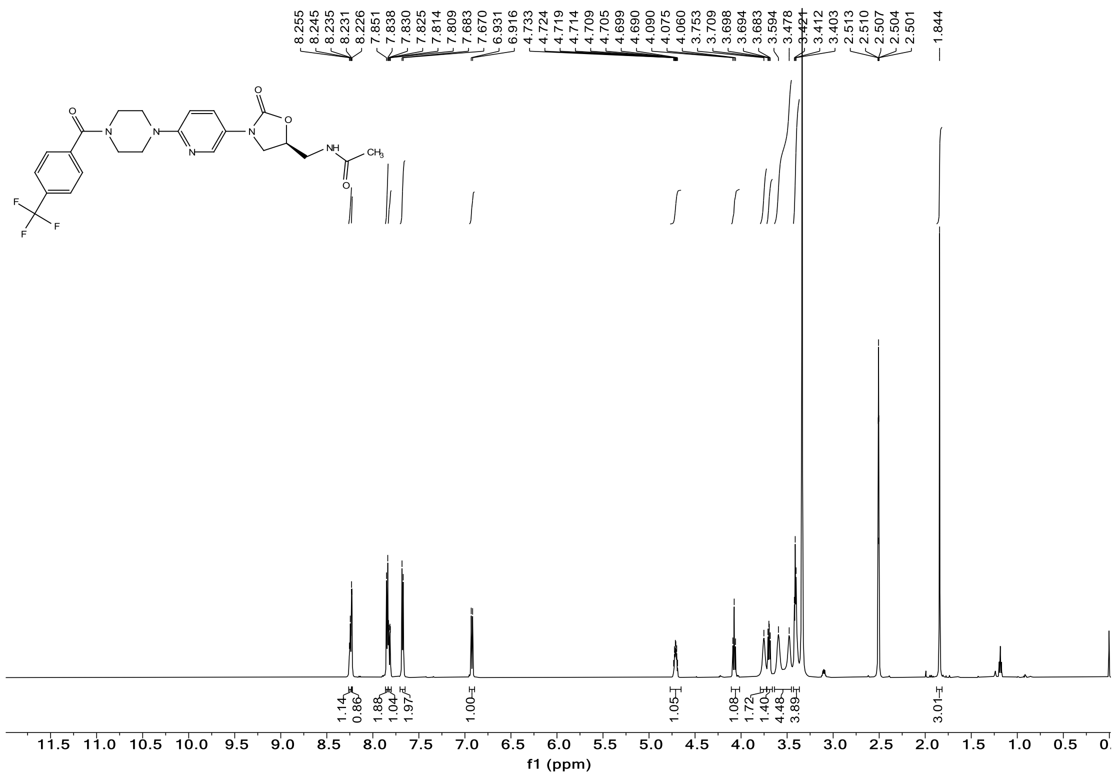

Fig. S64. ^{1}H NMR Spectrum (DMSO-d_6, 400 MHz) of **17c**.

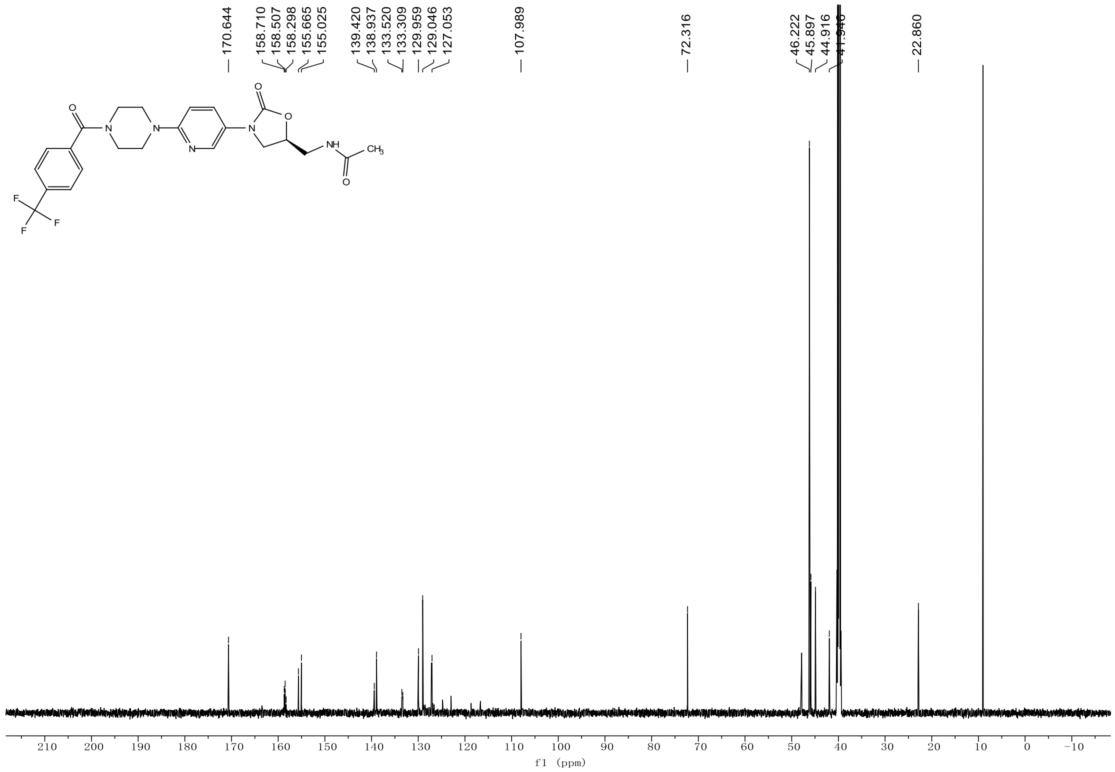

Fig. S65. ^{13}C NMR Spectrum (DMSO-d_6, 75 MHz) of **17c**.

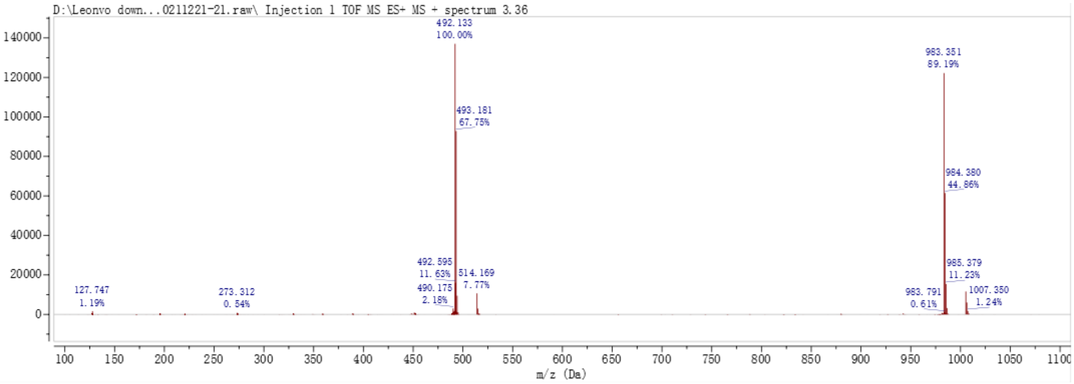

Fig. S66. MS calcd for $C_{23}H_{24}F_3N_5O_4$ (Mwt.: 491.47): m/z 492.133 ([M+H] $^+$, bp) of **17c**.

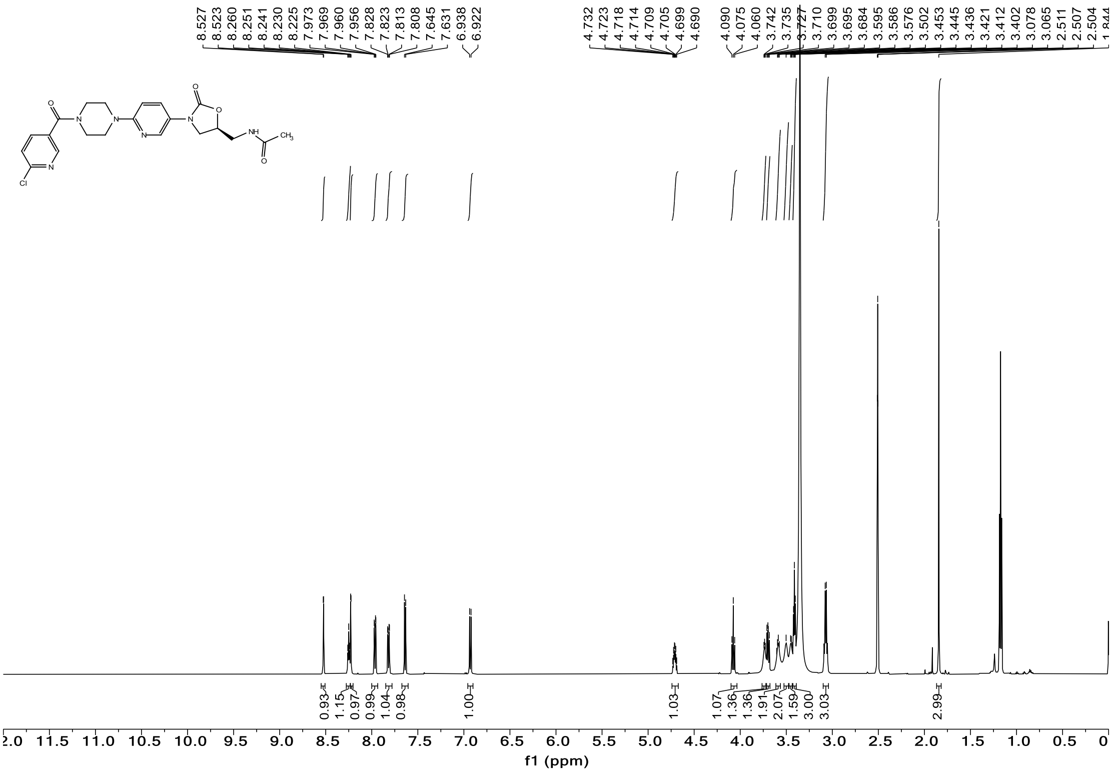

Fig. S67. ^{1}H NMR Spectrum (DMSO-d_6, 400 MHz) of **17d**.

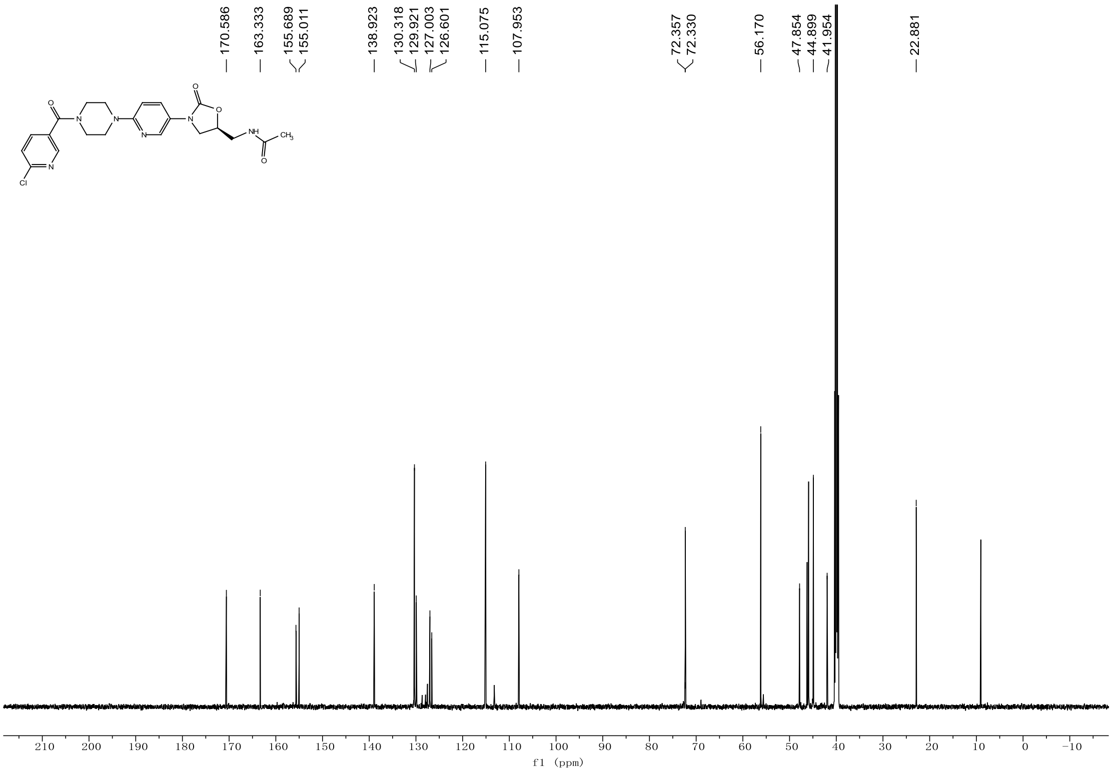

Fig. S68. ^{13}C NMR Spectrum (DMSO-d_6, 75 MHz) of **17d**.

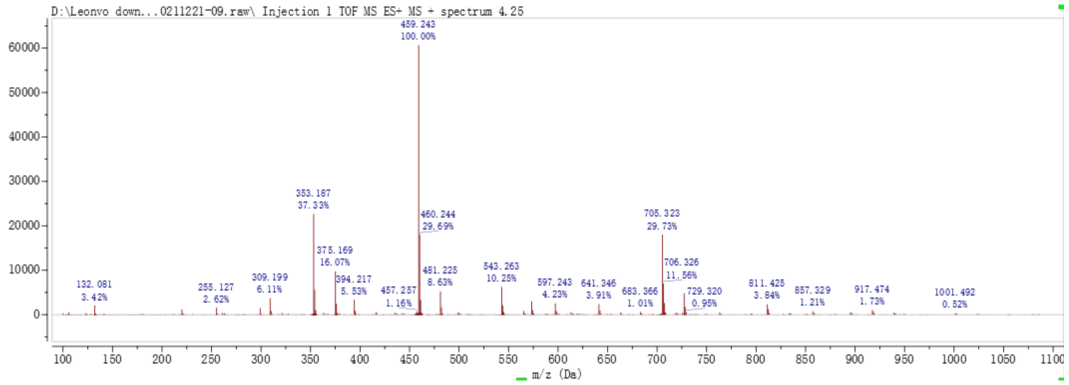

Fig. S69. MS calcd for $C_{21}H_{23}ClN_6O_4$ (Mwt.: 458.90): m/z 459.243 ([M+H] $^+$, bp) of **17d**.

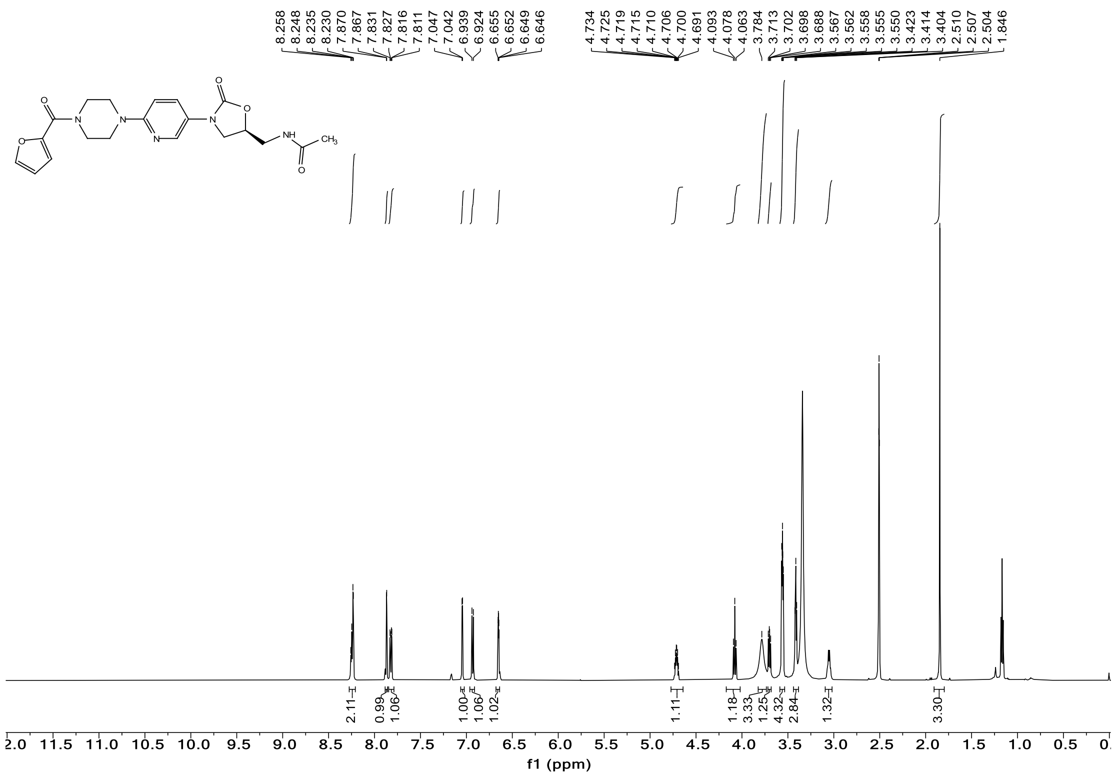

Fig. S70. ^{1}H NMR Spectrum (DMSO-d_6, 400 MHz) of **17e**.

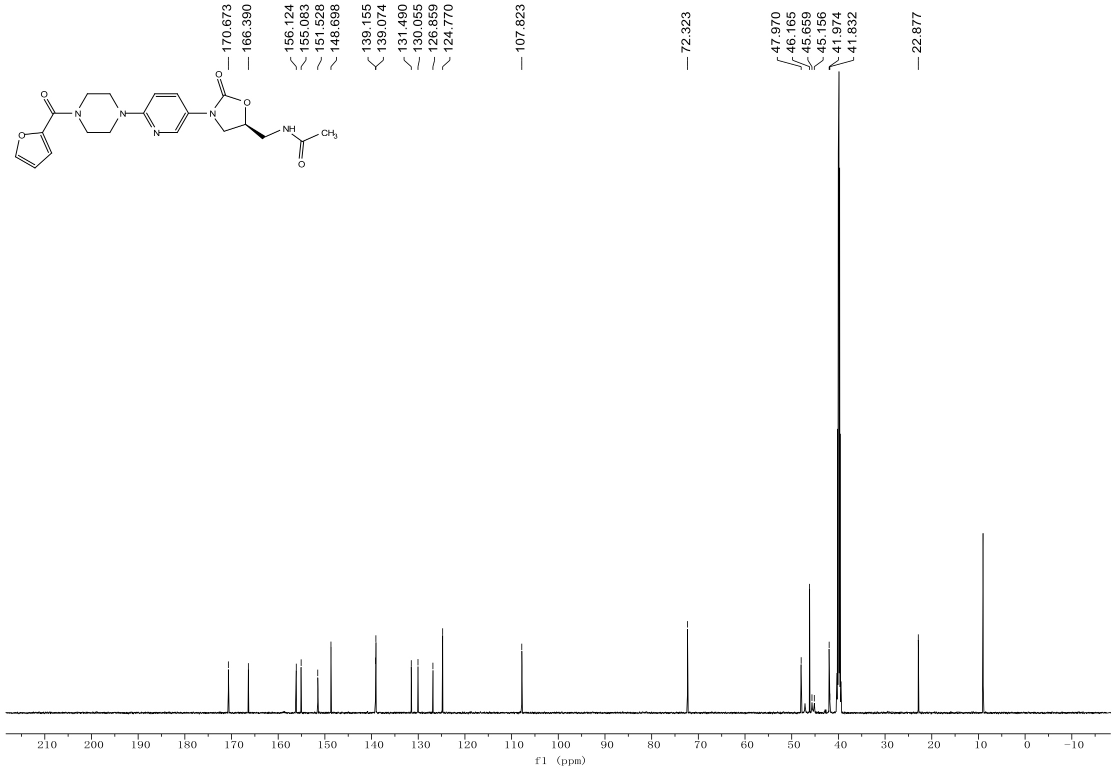

Fig. S71. ^{13}C NMR Spectrum (DMSO-d_6, 75 MHz) of **17e**.

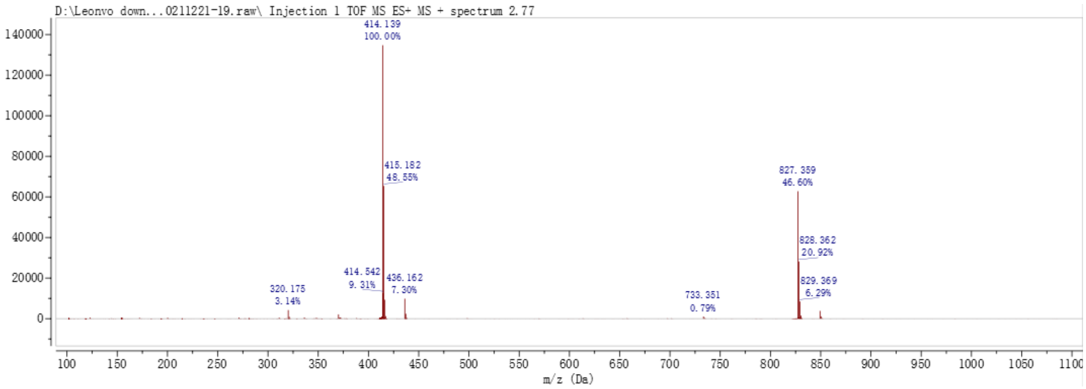

Fig. S72. MS calcd for $C_{20}H_{23}N_5O_5$ (Mwt.: 413.43): m/z 414.139 ([M+H] $^{+}$, bp) of **17e**.

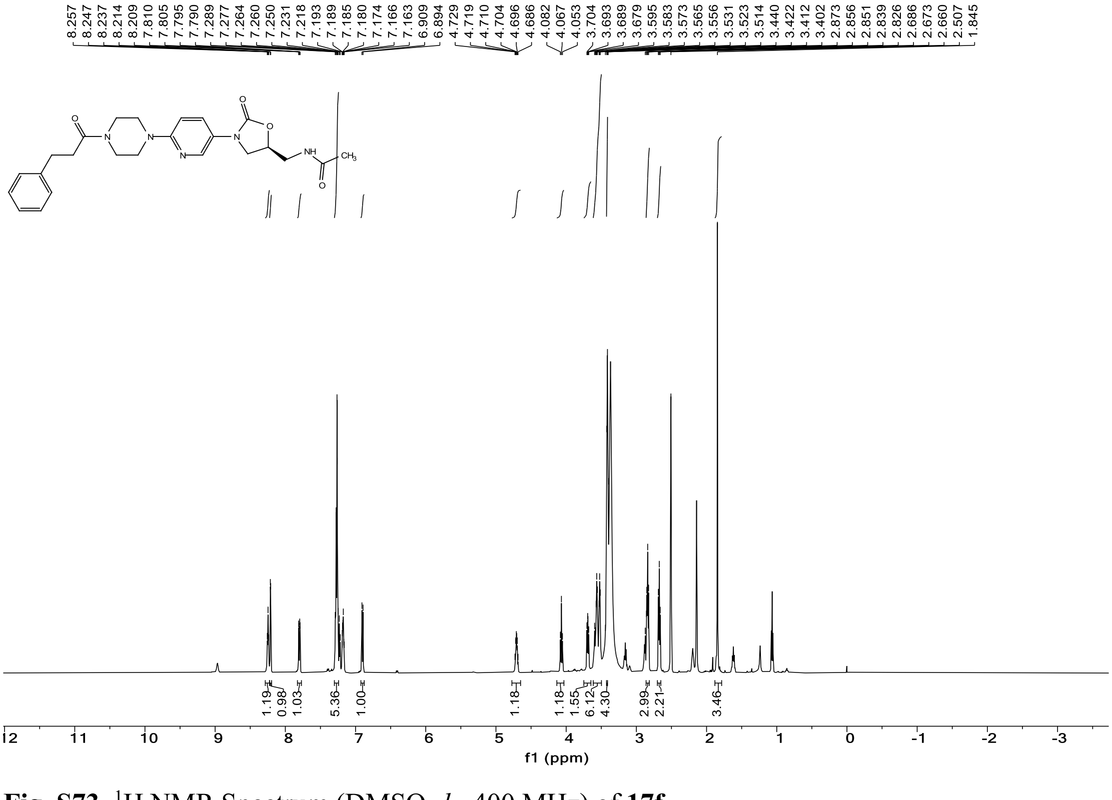

Fig. S73. ^{1}H NMR Spectrum (DMSO-d_6, 400 MHz) of **17f**.

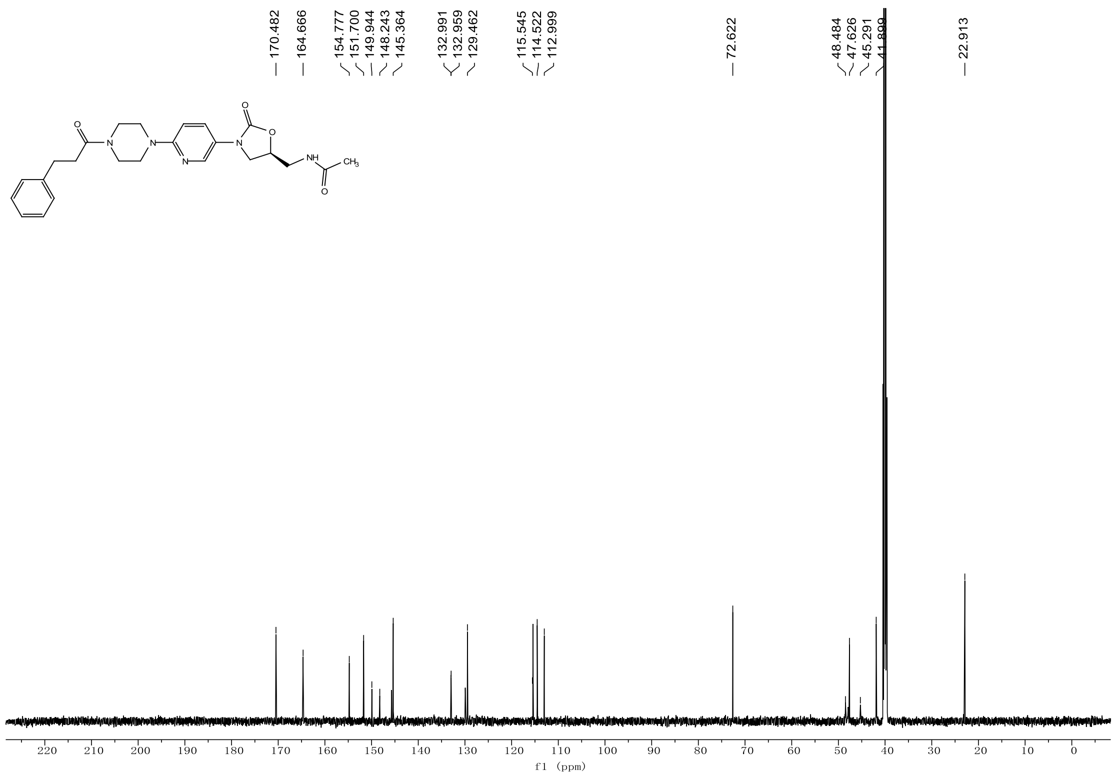

Fig. S74. ^{13}C NMR Spectrum (DMSO-d_6, 75 MHz) of **17f**.

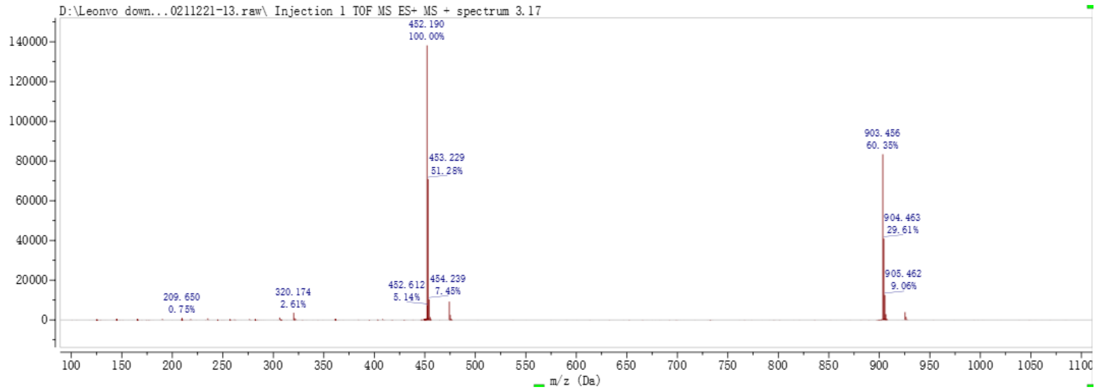

Fig. S75. MS calcd for $C_{24}H_{29}N_5O_4$ (Mwt.: 451.53): m/z 452.190 ([M+H] $^+$, bp) of **17f**.

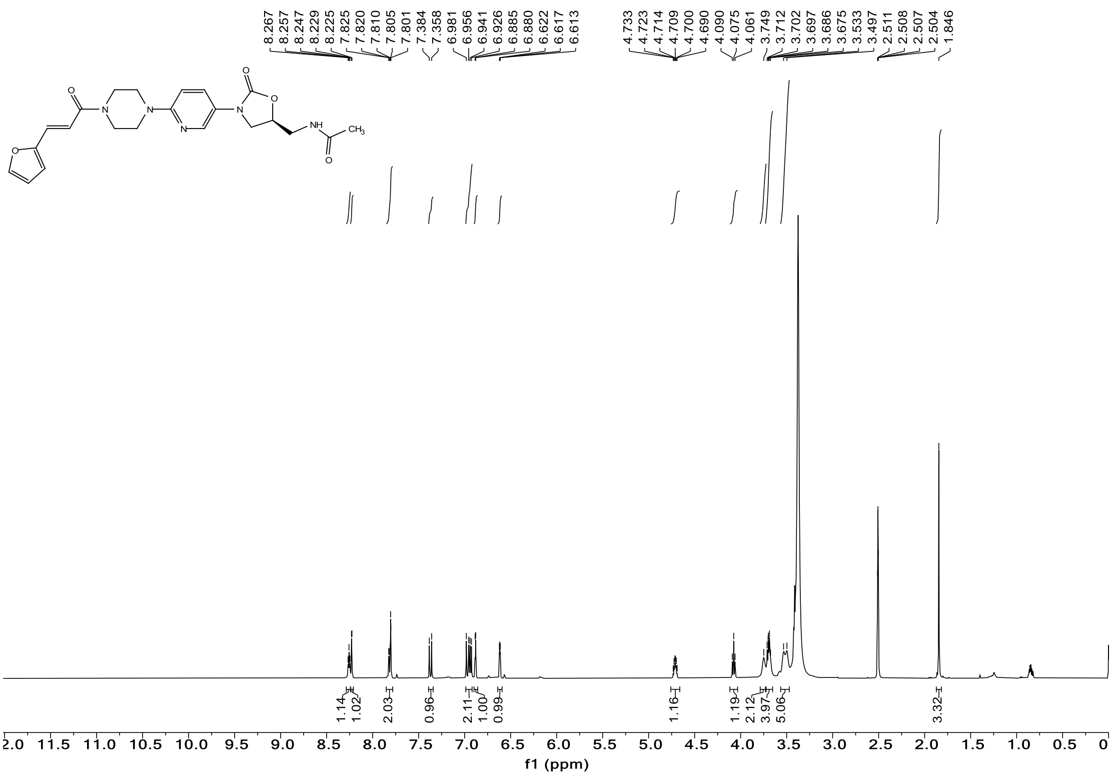

Fig. S76. ^{1}H NMR Spectrum (DMSO-d_6, 400 MHz) of **17g**.

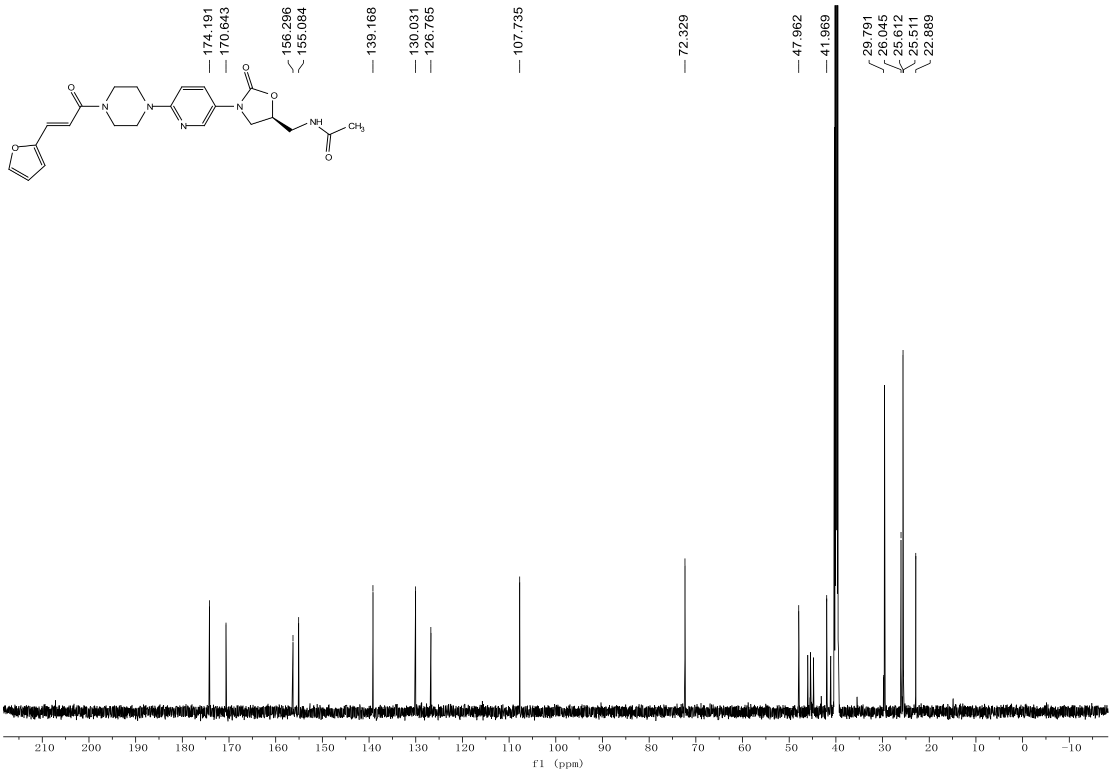

Fig. S77. ^{13}C NMR Spectrum (DMSO-d_6, 75 MHz) of **17g**.

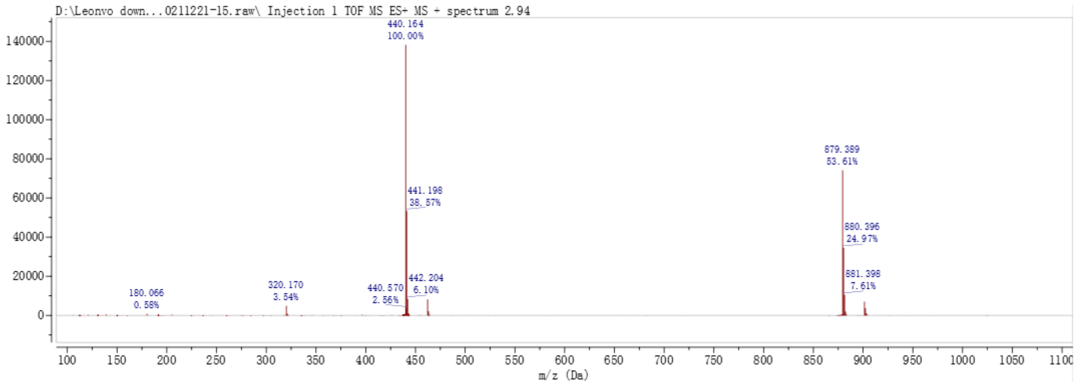

Fig. S78. MS calcd for $C_{22}H_{25}N_5O_5$ (Mwt.: 439.47): m/z 440.164 ([M+H] $^+$, bp) of **17g**.

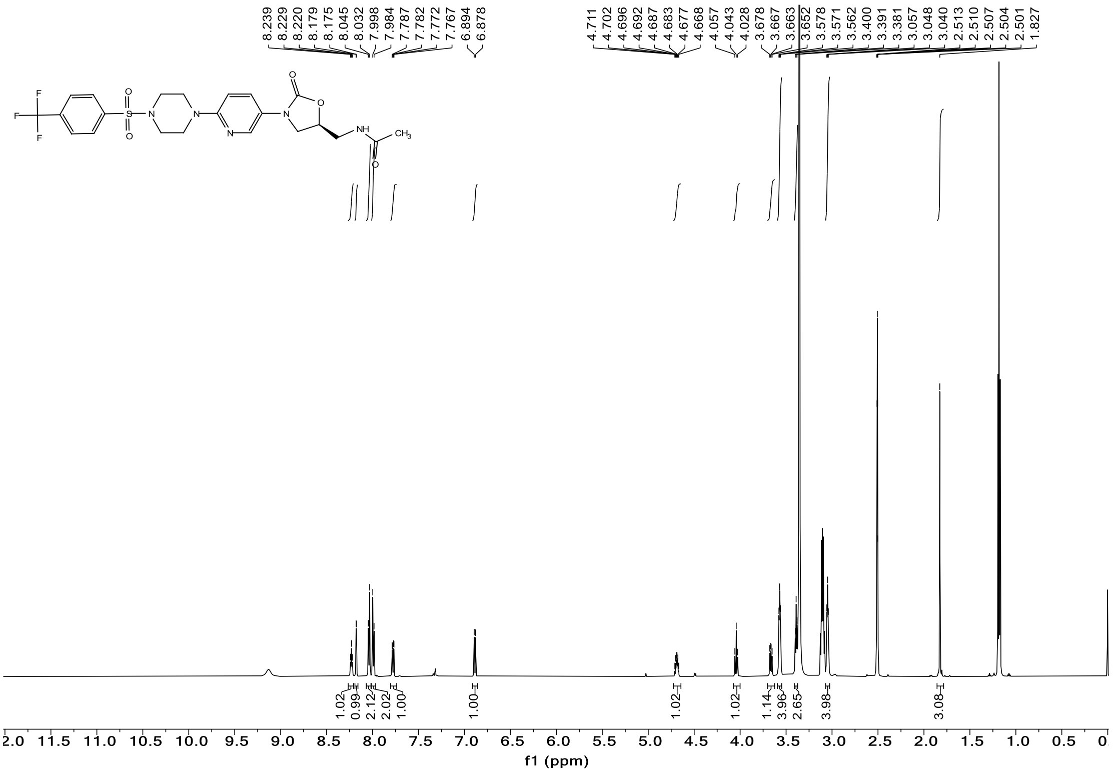

Fig. S79. ^{1}H NMR Spectrum (DMSO-d_6, 400 MHz) of **17h**.

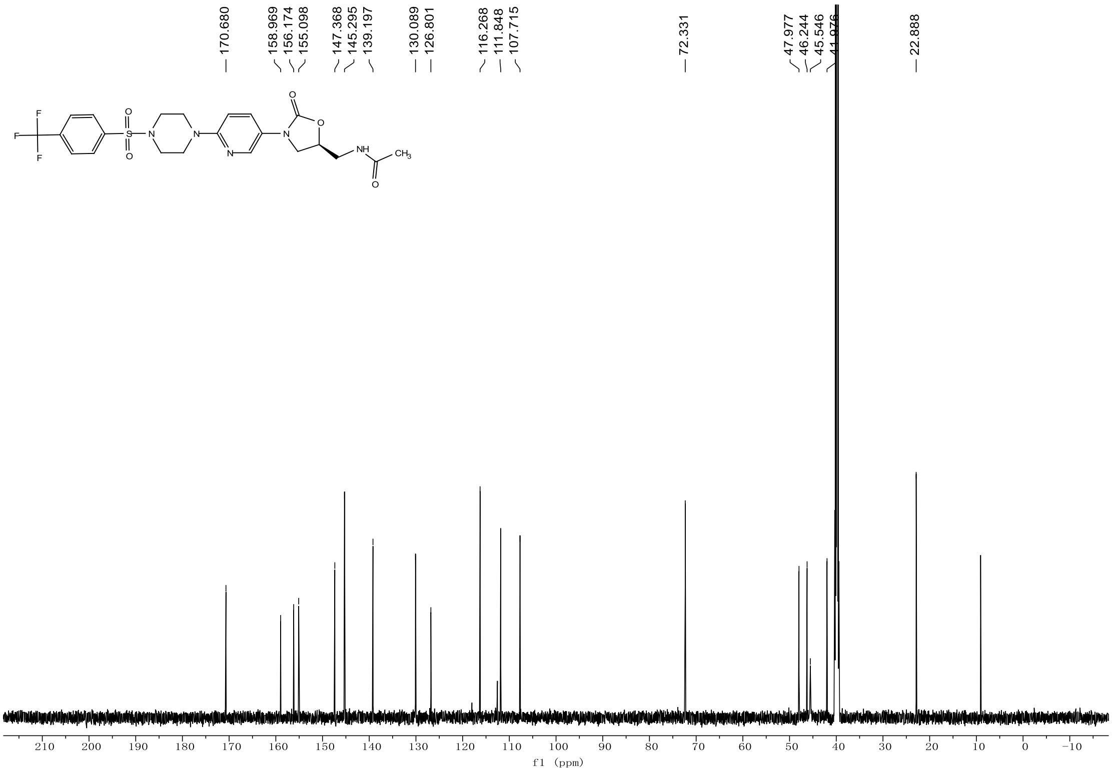

Fig. S80. ^{13}C NMR Spectrum (DMSO-d_6, 75 MHz) of **17h**.

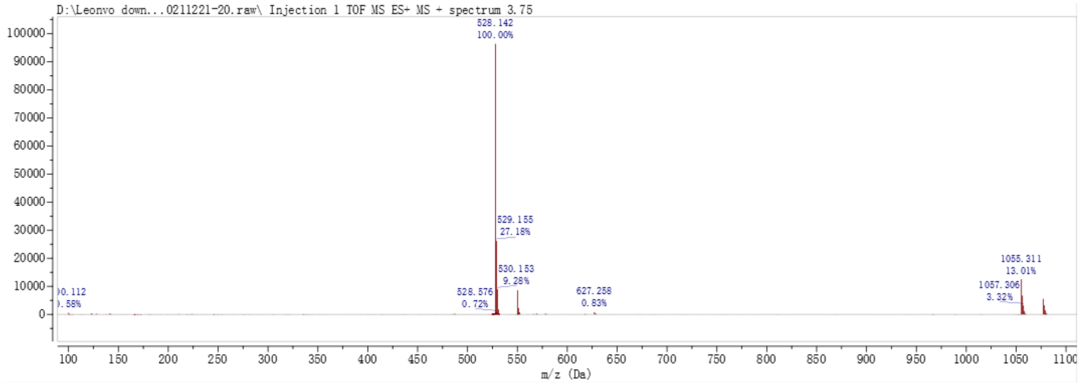

Fig. S81. MS calcd for $C_{22}H_{24}F_3N_5O_5S$ (Mwt.: 527.52): m/z 528.142 ([M+H] $^+$, bp) of **17h**.

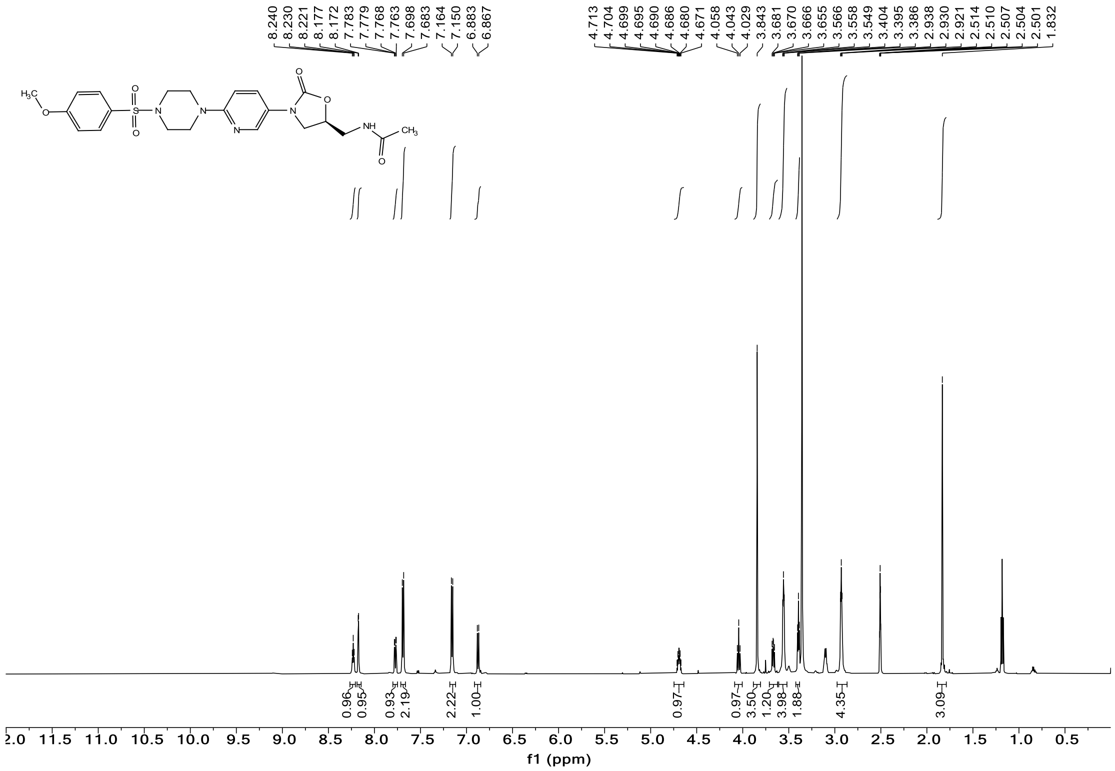

Fig. S82. ^{1}H NMR Spectrum (DMSO-d_6, 400 MHz) of **17i**.

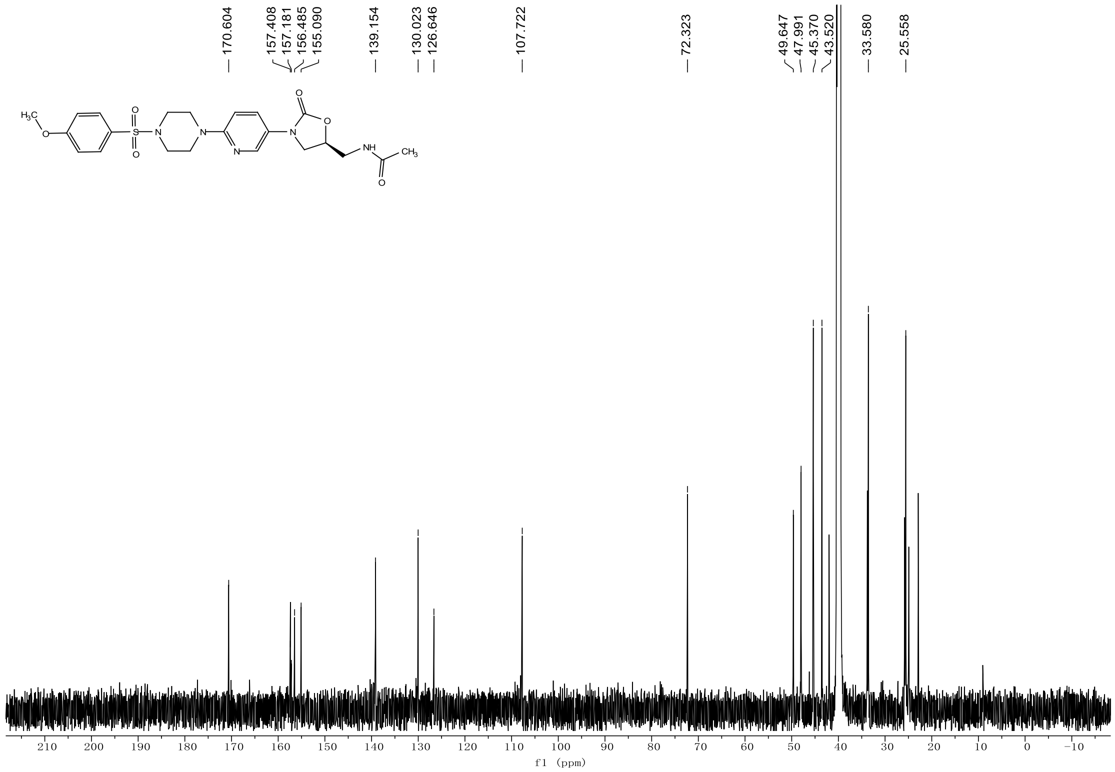

Fig. S83. ^{13}C NMR Spectrum (DMSO-d_6, 75 MHz) of **17i**.

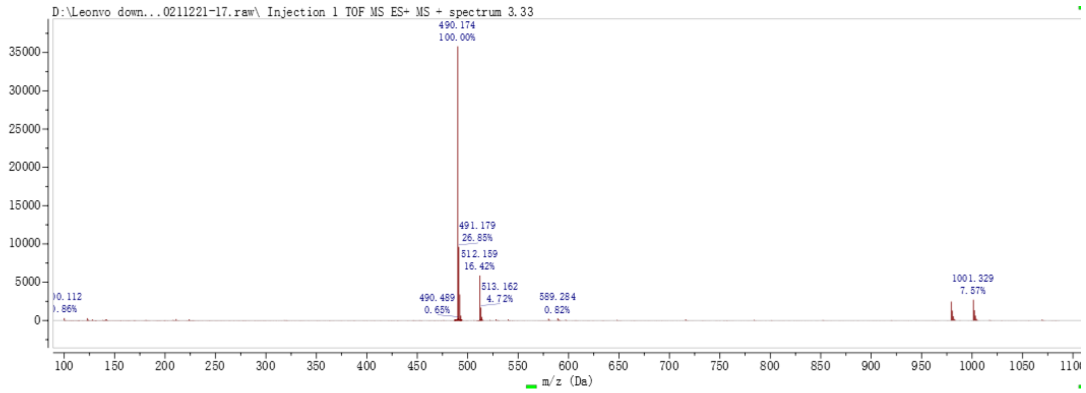

Fig. S84. MS calcd for $C_{22}H_{27}N_5O_6S$ (Mwt.: 489.55): m/z 490.174 ([M+H] $^+$, bp) of **17i**.

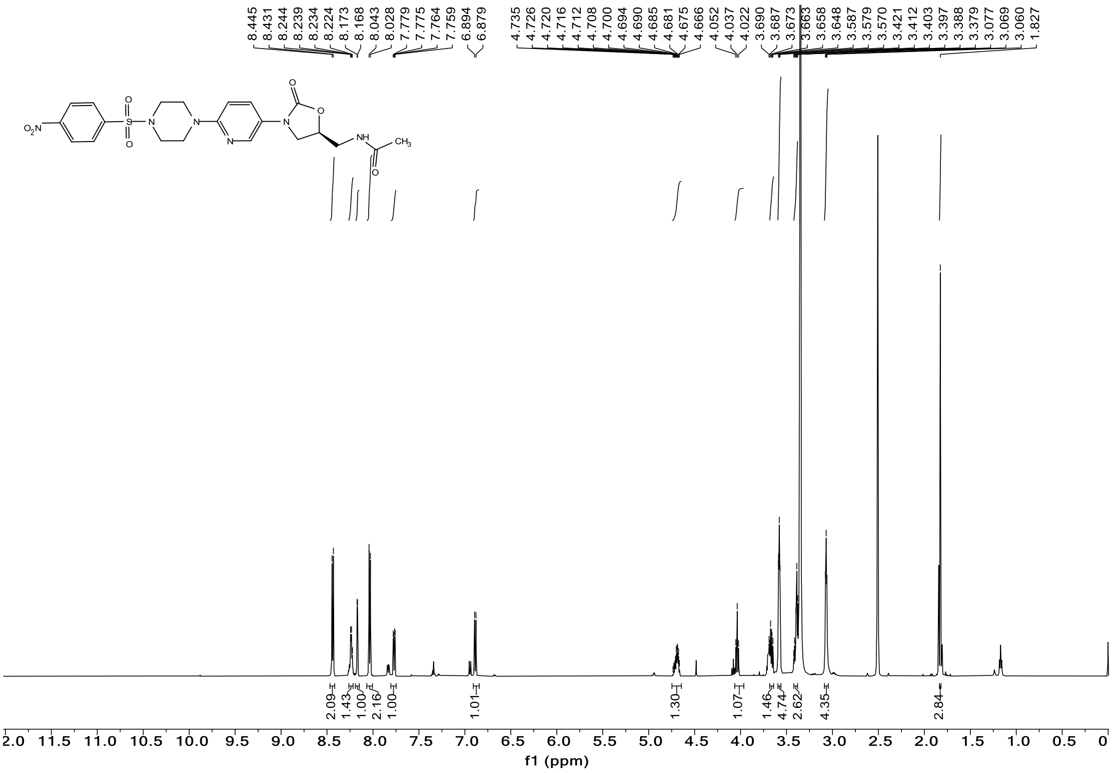

Fig. S85. ^{1}H NMR Spectrum (DMSO-d_6, 400 MHz) of **17j**.

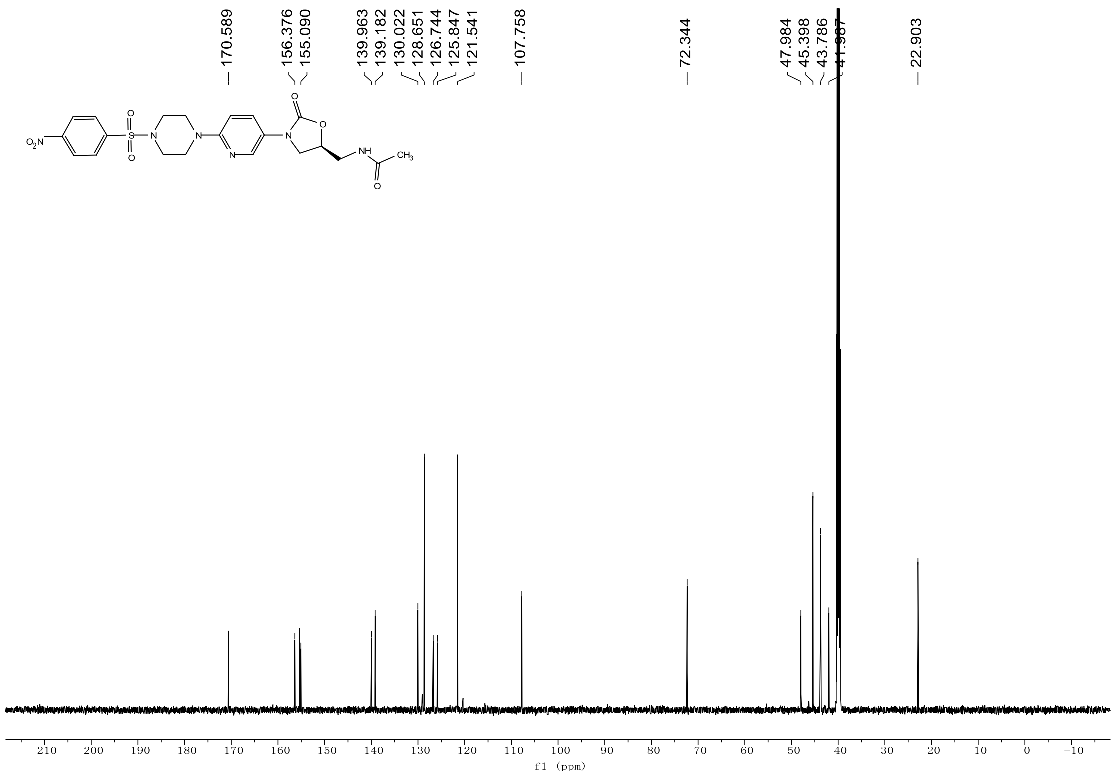

Fig. S86. ^{13}C NMR Spectrum (DMSO-d_6, 75 MHz) of **17j**.

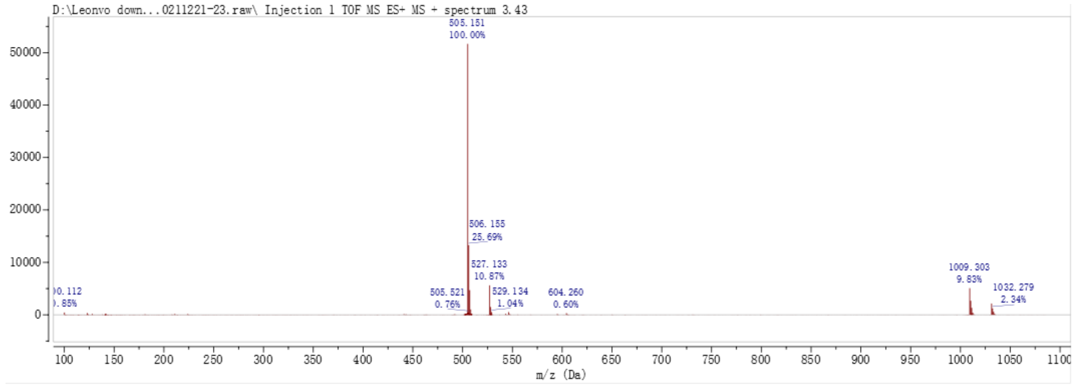

Fig. S87. MS calcd for $C_{21}H_{24}N_6O_7S$ (Mwt.: 504.52): m/z 505.151 ($[M+H]^+$, bp) of **17j**.

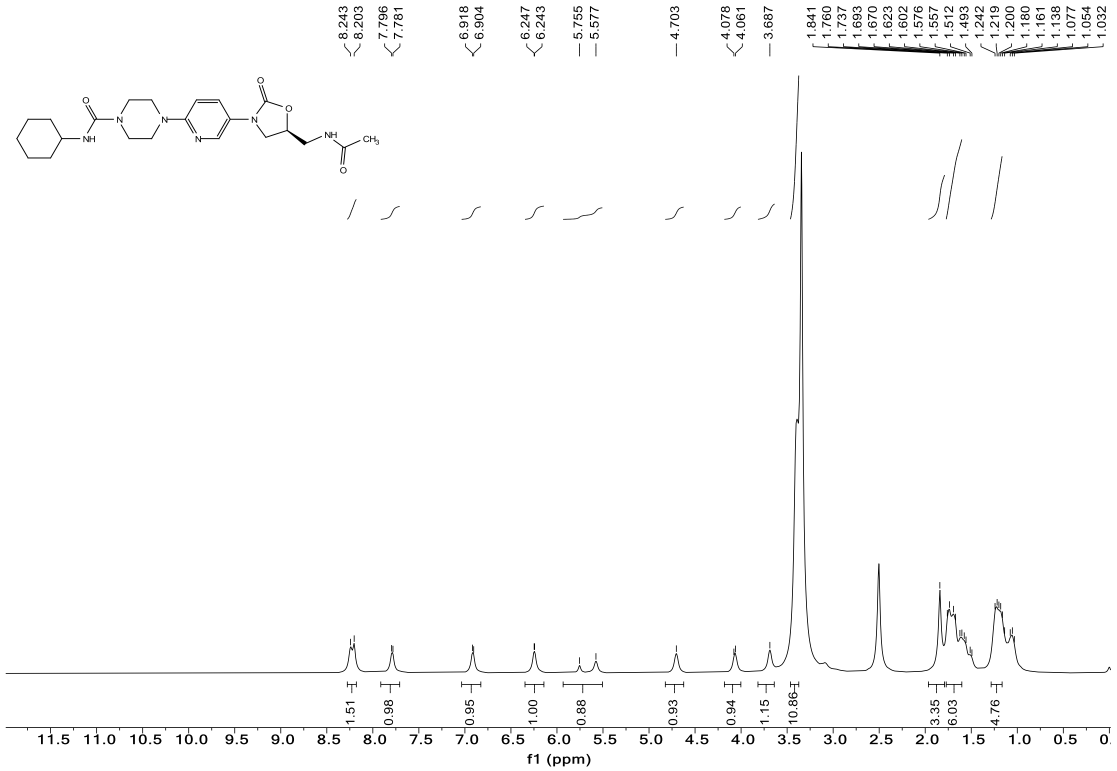

Fig. S88. ^{1}H NMR Spectrum (DMSO-d_6, 400 MHz) of **17k**.

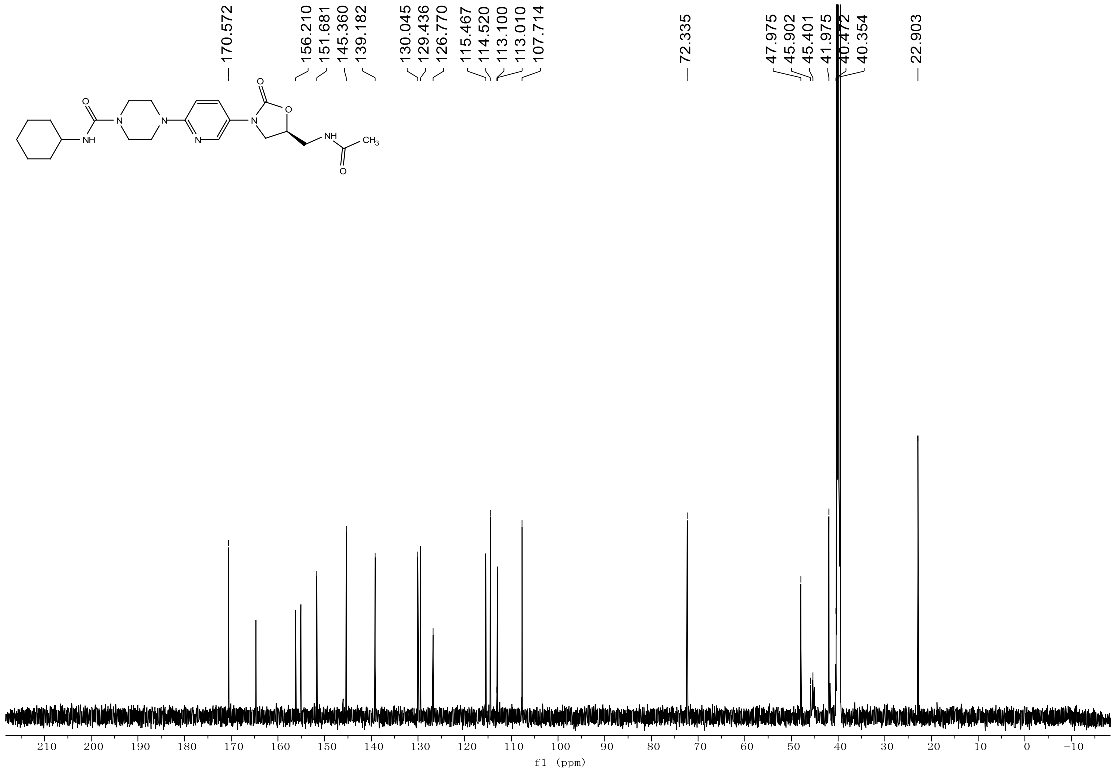

Fig. S89. ^{13}C NMR Spectrum (DMSO-d_6, 75 MHz) of **17k**.

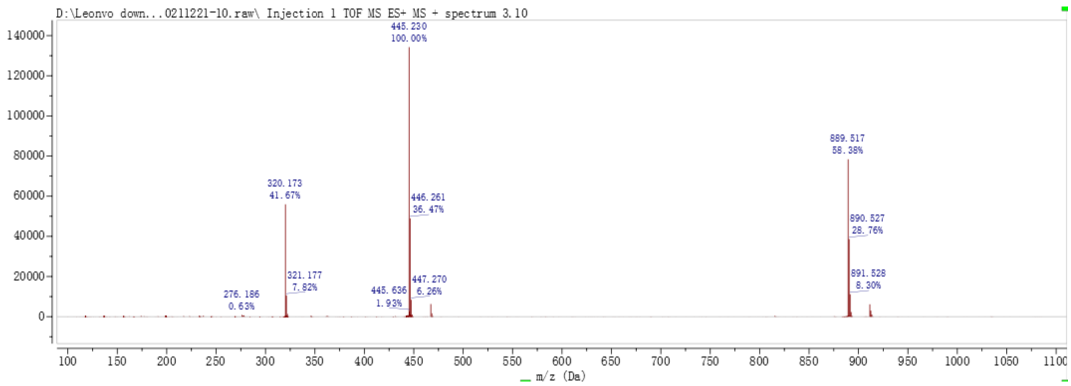

Fig. S90. MS calcd for $C_{22}H_{32}N_6O_4$ (Mwt.: 444.54): m/z 445.230 ([M+H] $^+$, bp) of **17k**.

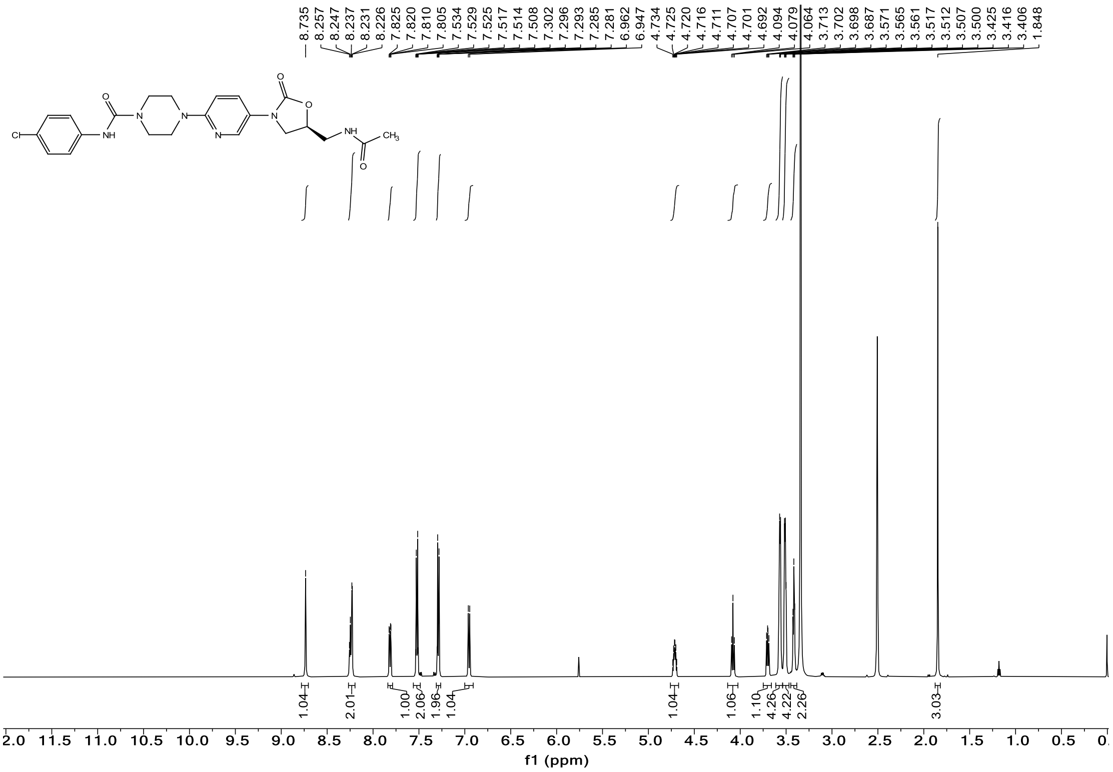

Fig. S91. ^{1}H NMR Spectrum (DMSO-d_6, 400 MHz) of **17l**.

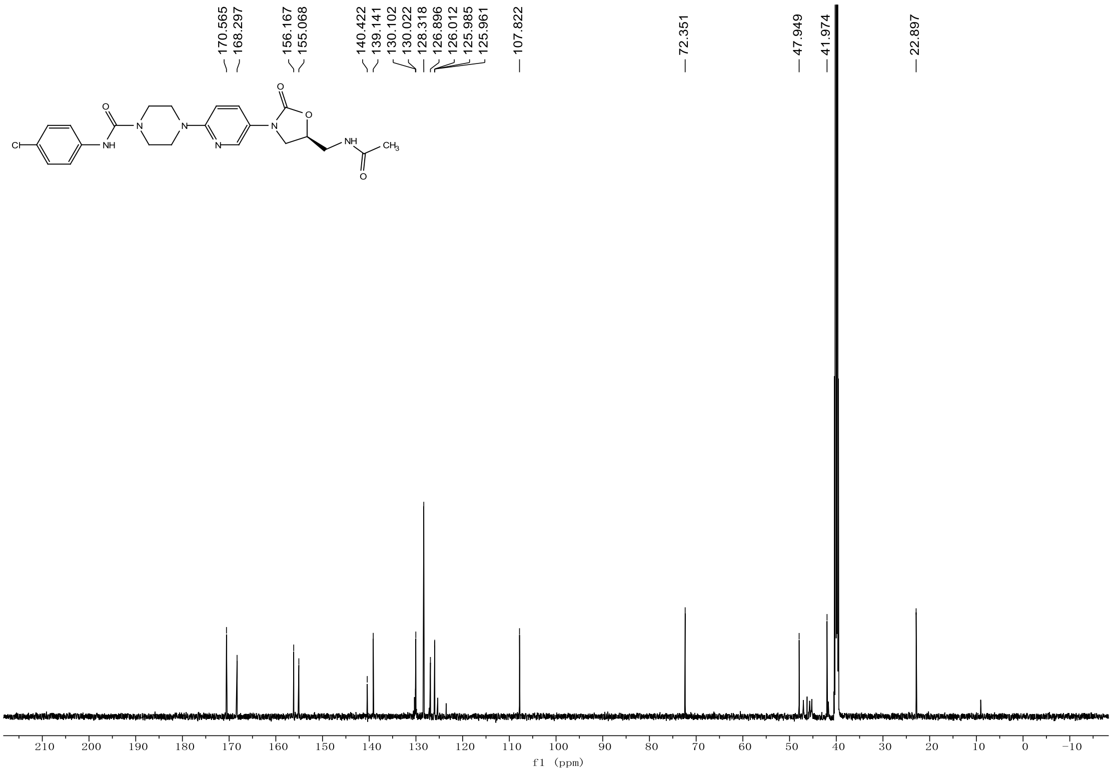

Fig. S92. ^{13}C NMR Spectrum (DMSO-d_6, 75 MHz) of **17l**.

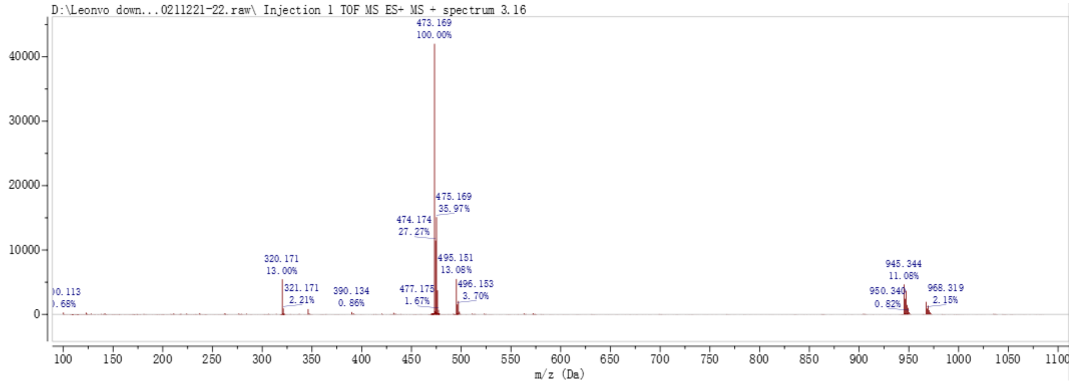

Fig. S93. MS calcd for $C_{22}H_{25}ClN_6O_4$ (Mwt.: 472.93): m/z 473.169 ([M+H] $^+$, bp) of **17l**.

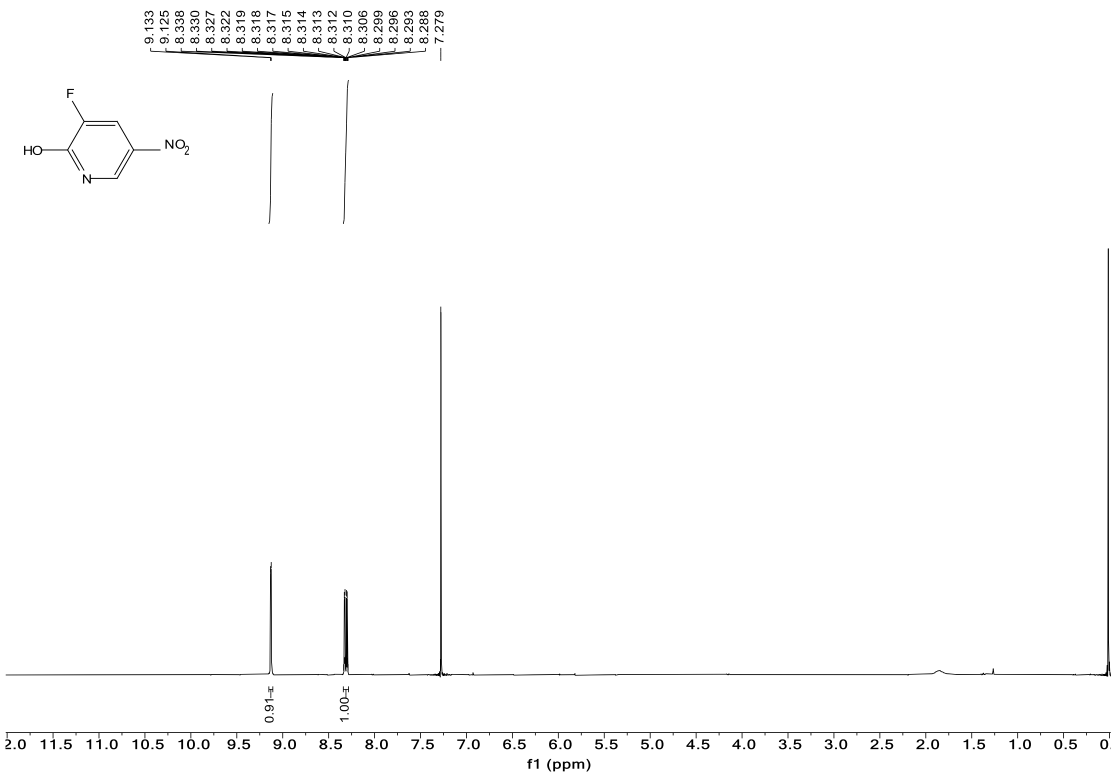

Fig. S94. ^{1}H NMR Spectrum ($CDCl_3$, 400 MHz) of **19**.

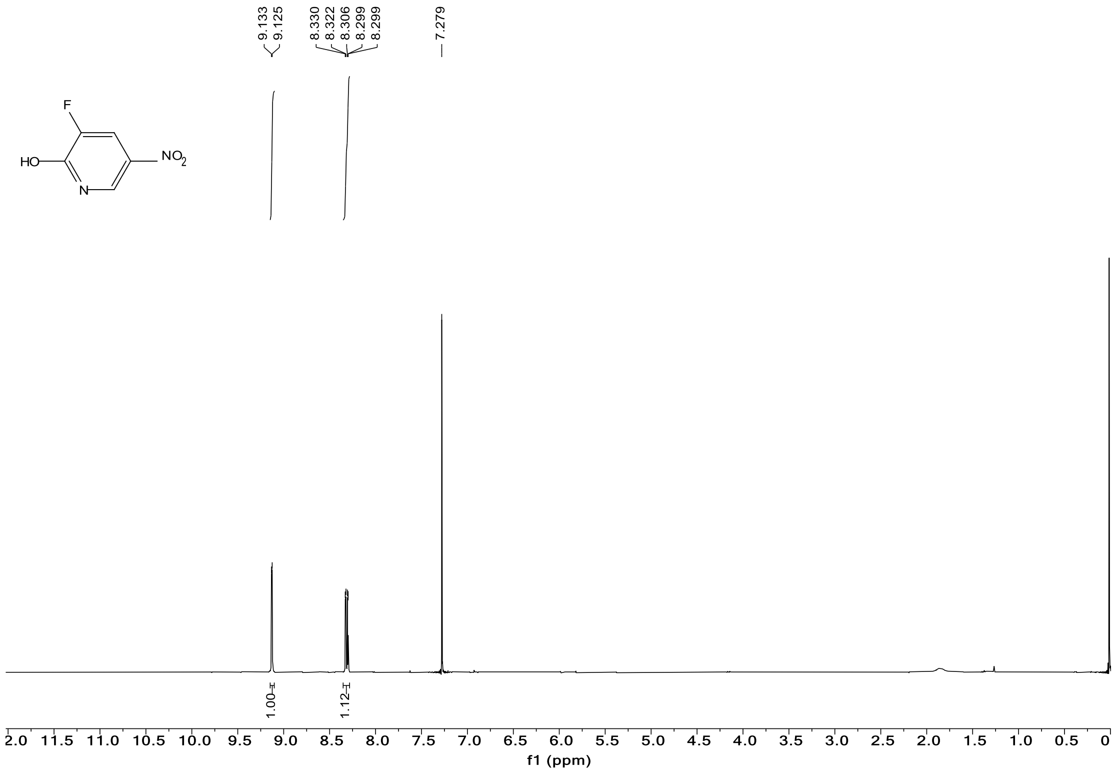

Fig. S95. ^{1}H NMR Spectrum ($CDCl_3$, 400 MHz) of **20**.

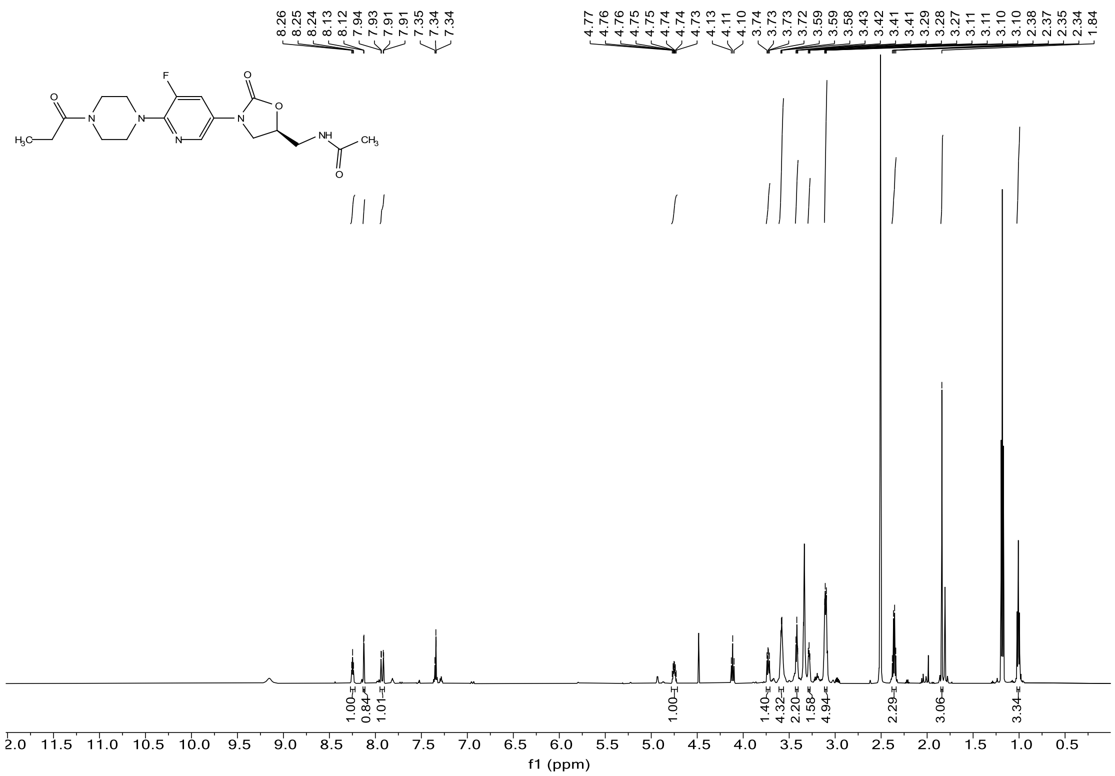

Fig. S96. ^{1}H NMR Spectrum (DMSO-d_6, 400 MHz) of **21a**.

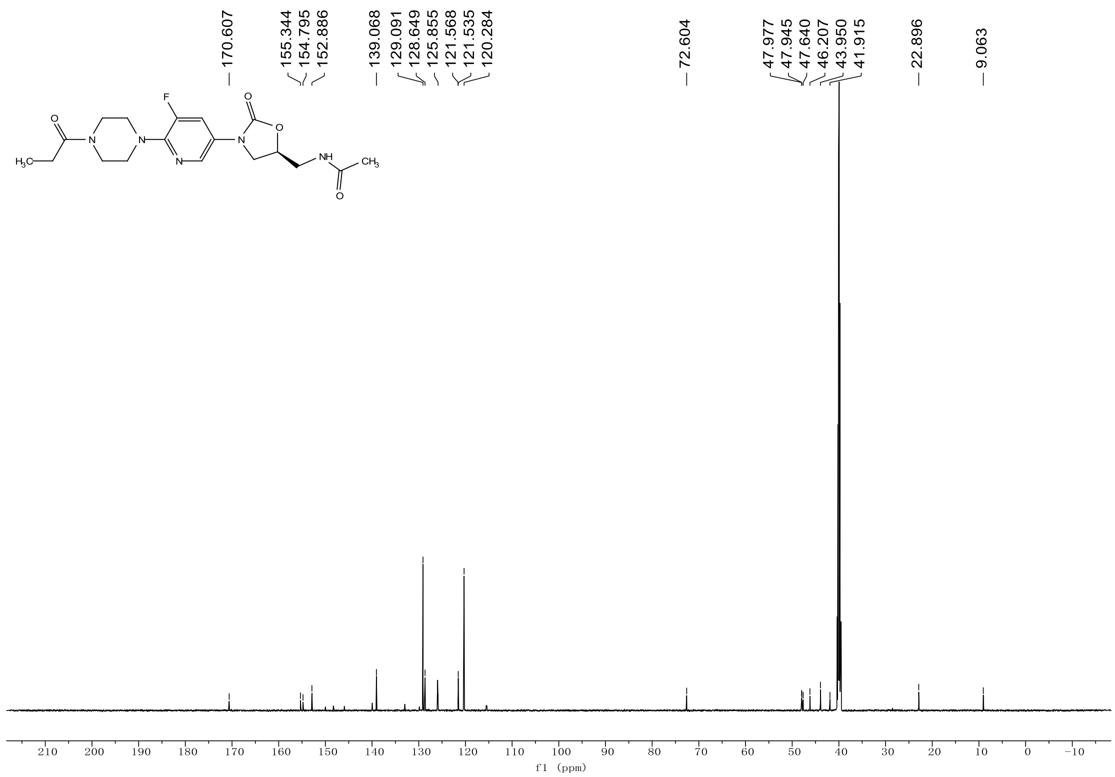

Fig. S97. ^{13}C NMR Spectrum (DMSO-d_6, 75 MHz) of **21a**.

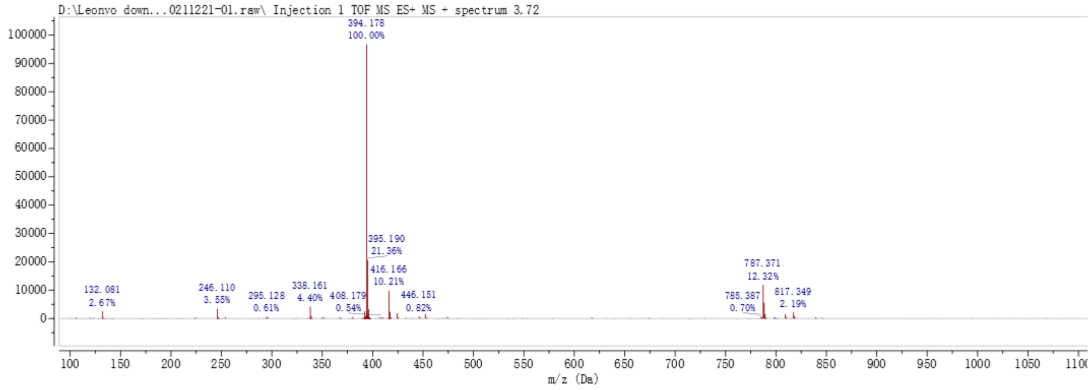

Fig. S98. MS calcd for $C_{18}H_{24}FN_5O_4$ (Mwt.: 393.42): m/z 394.178 ([M+H] $^+$, bp) of **21a**.

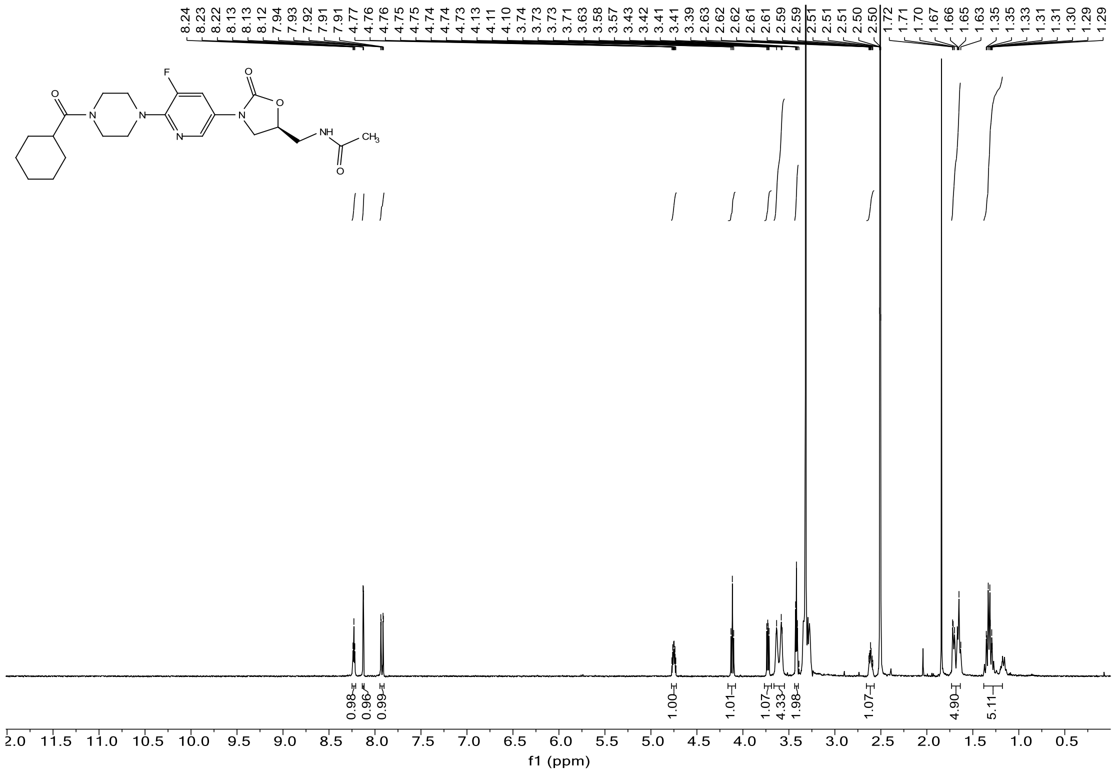

Fig. S99. ^{1}H NMR Spectrum (DMSO-d_6, 400 MHz) of **21b**.

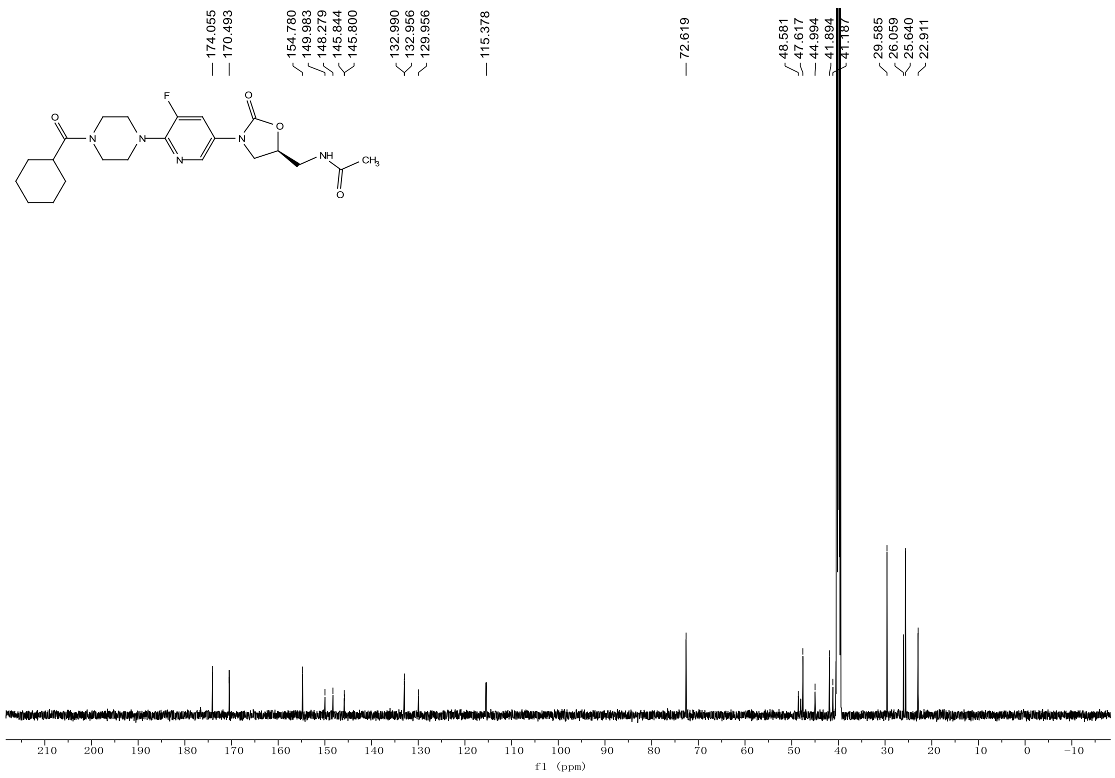

Fig. S100. ^{13}C NMR Spectrum (DMSO-d_6, 75 MHz) of **21b**.

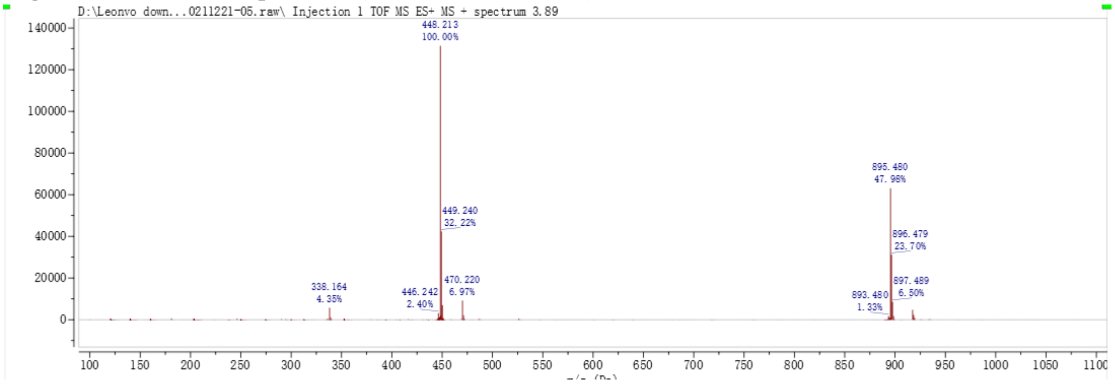

Fig. S101. MS calcd for $C_{22}H_{30}FN_5O_4$ (Mwt.: 447.51): m/z 448.213 ($[M+H]^+$, bp) of **21b**.

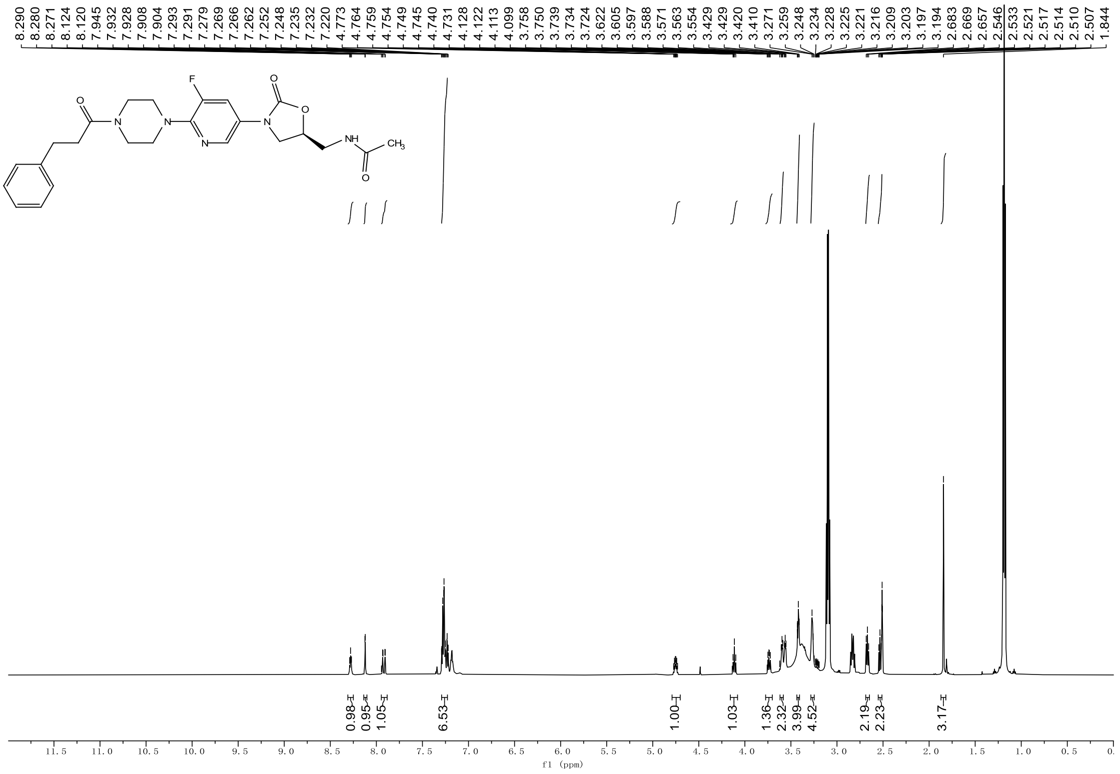

Fig. S102. ^{1}H NMR Spectrum (DMSO-d_6, 400 MHz) of **21c**.

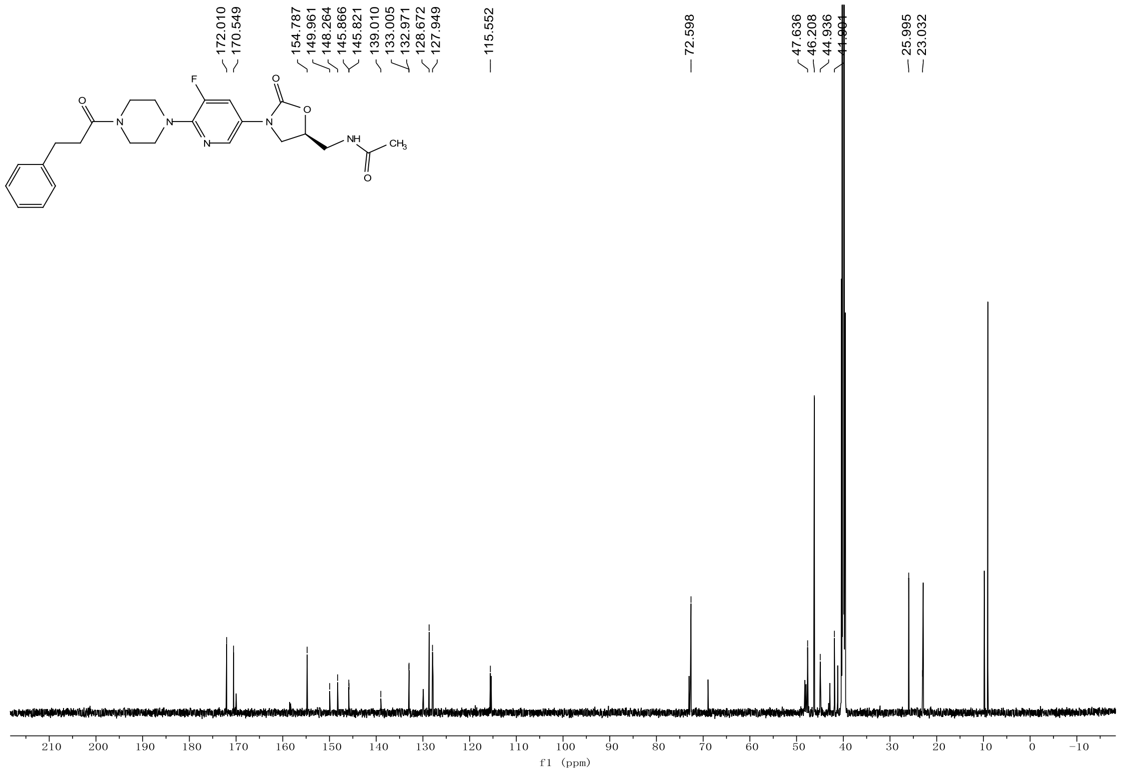

Fig. S103. ^{13}C NMR Spectrum (DMSO-d_6, 75 MHz) of **21c**.

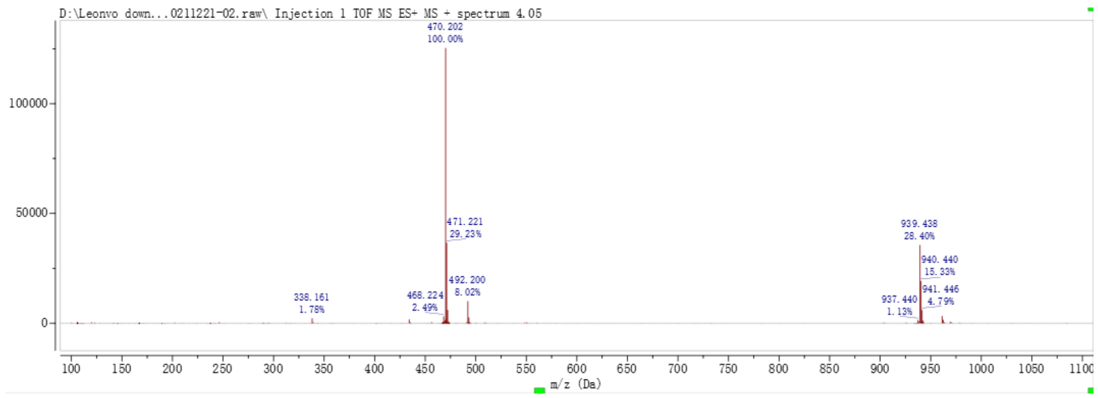

Fig. S104. MS calcd for $C_{24}H_{28}FN_5O_4$ (Mwt.: 469.52): m/z 470.202 ([M+H] $^+$, bp) of **21c**.

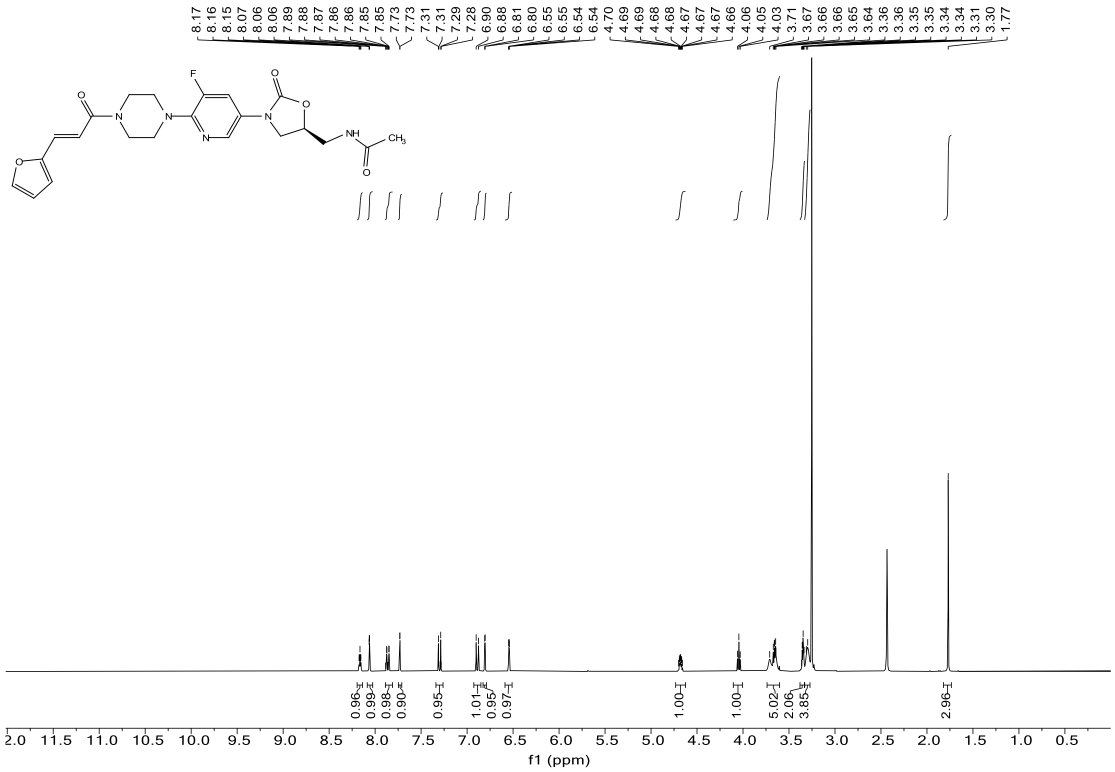

Fig. S105. ^{1}H NMR Spectrum (DMSO-d_6, 400 MHz) of **21d**.

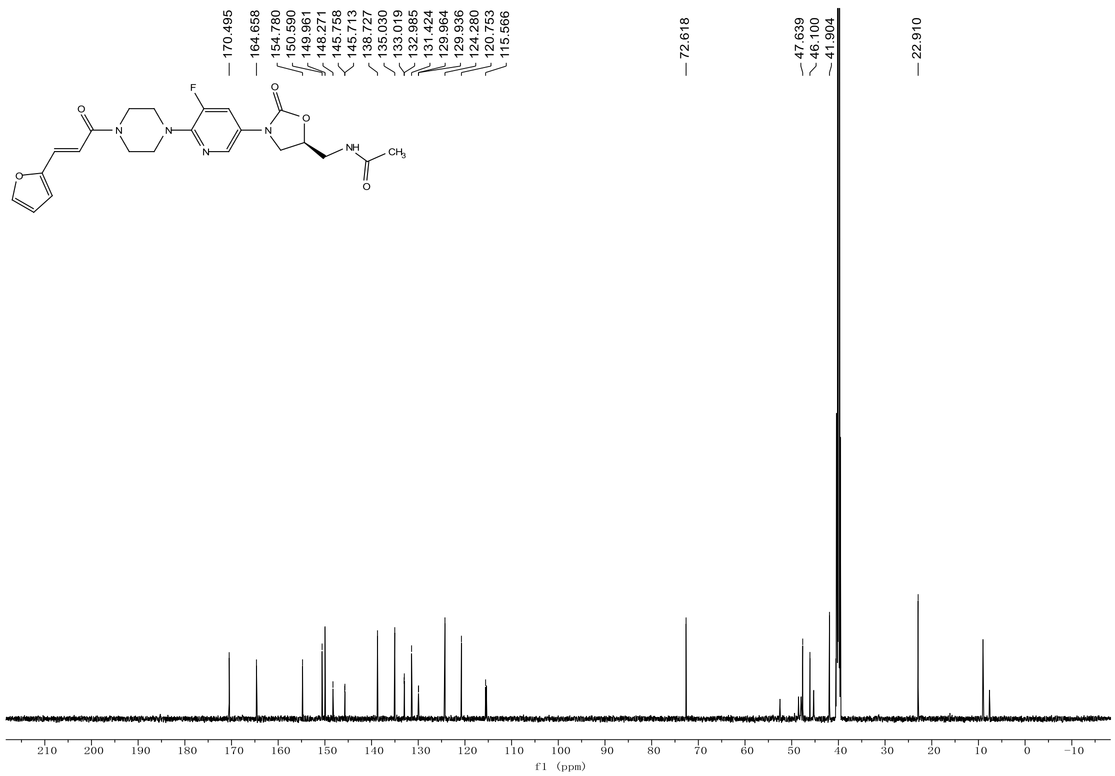

Fig. S106. ^{13}C NMR Spectrum (DMSO-d_6, 75 MHz) of **21d**.

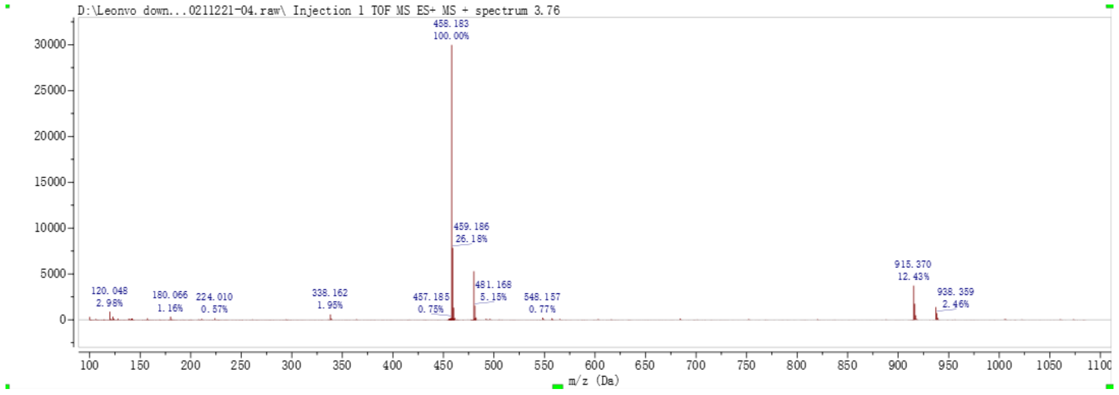

Fig. S107. MS calcd for $C_{22}H_{24}FN_5O_5$ (Mwt.: 457.46): m/z 458.183 ([M+H] $^+$, bp) of **21d**.

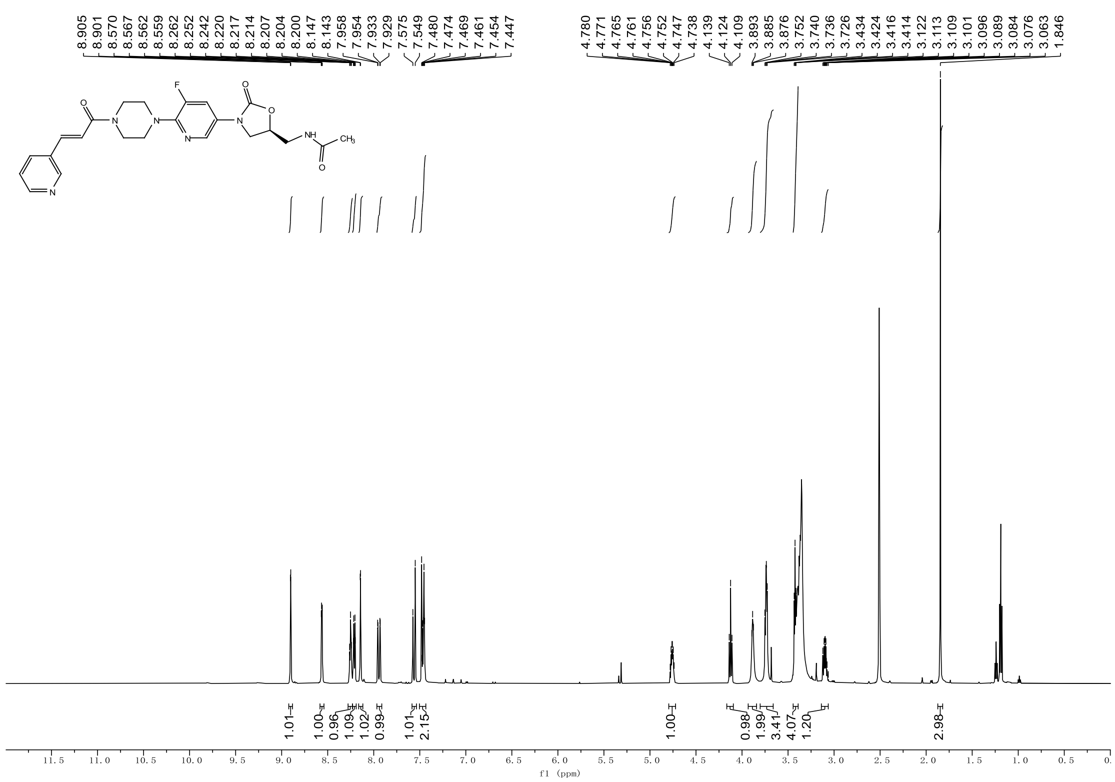

Fig. S108. ^{1}H NMR Spectrum (DMSO-d_6, 400 MHz) of **21e**.

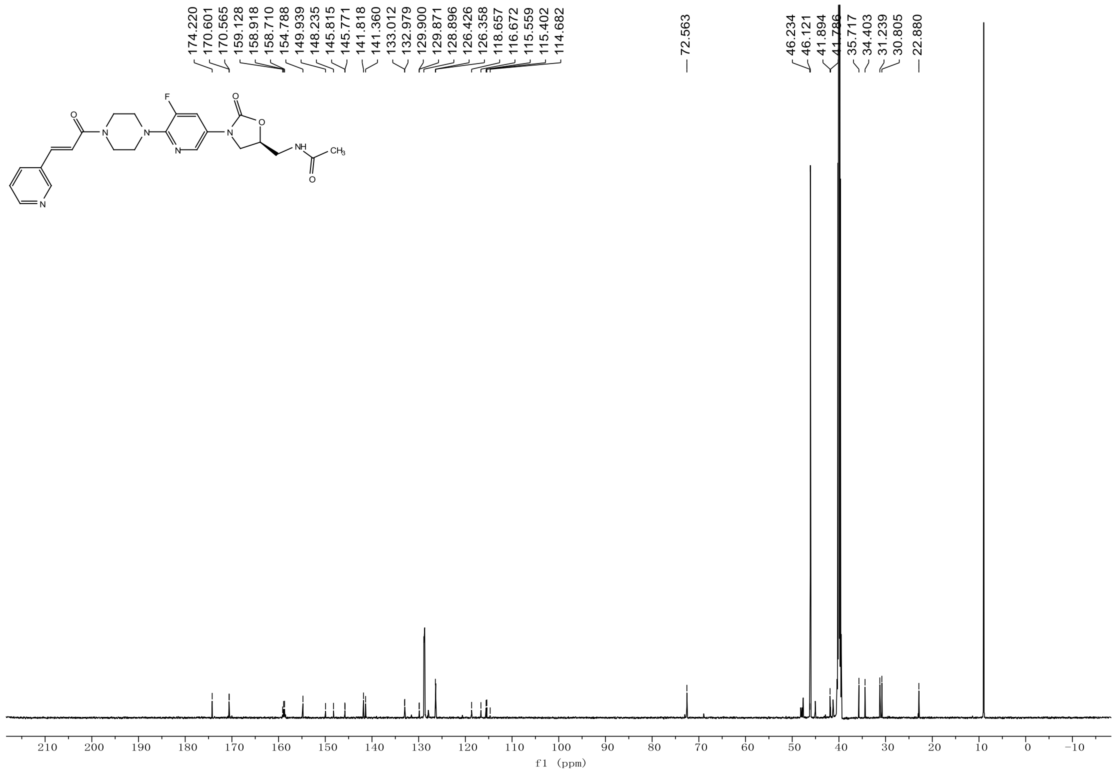

Fig. S109. ^{13}C NMR Spectrum (DMSO-d_6, 75 MHz) of **21e**.

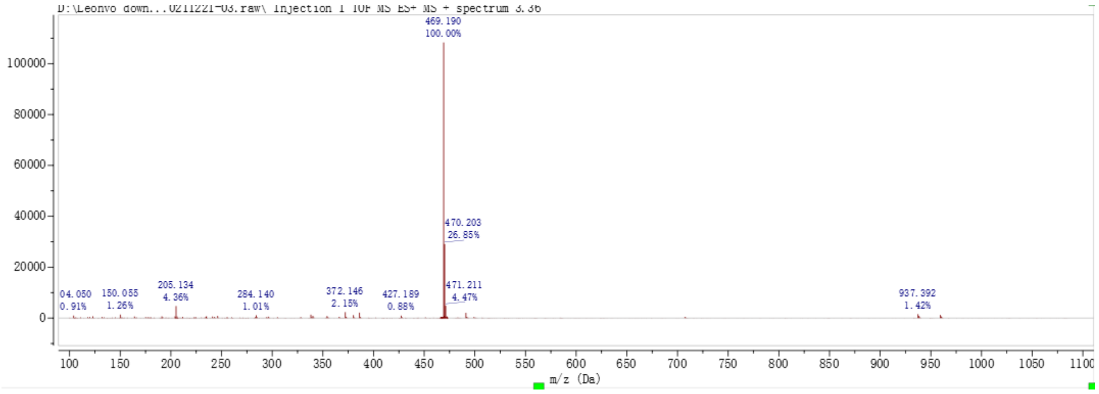

Fig. S110. MS calcd for $C_{23}H_{25}FN_6O_4$ (Mwt.: 468.49): m/z 469.19 ([M+H] $^+$, bp) of **21e**.

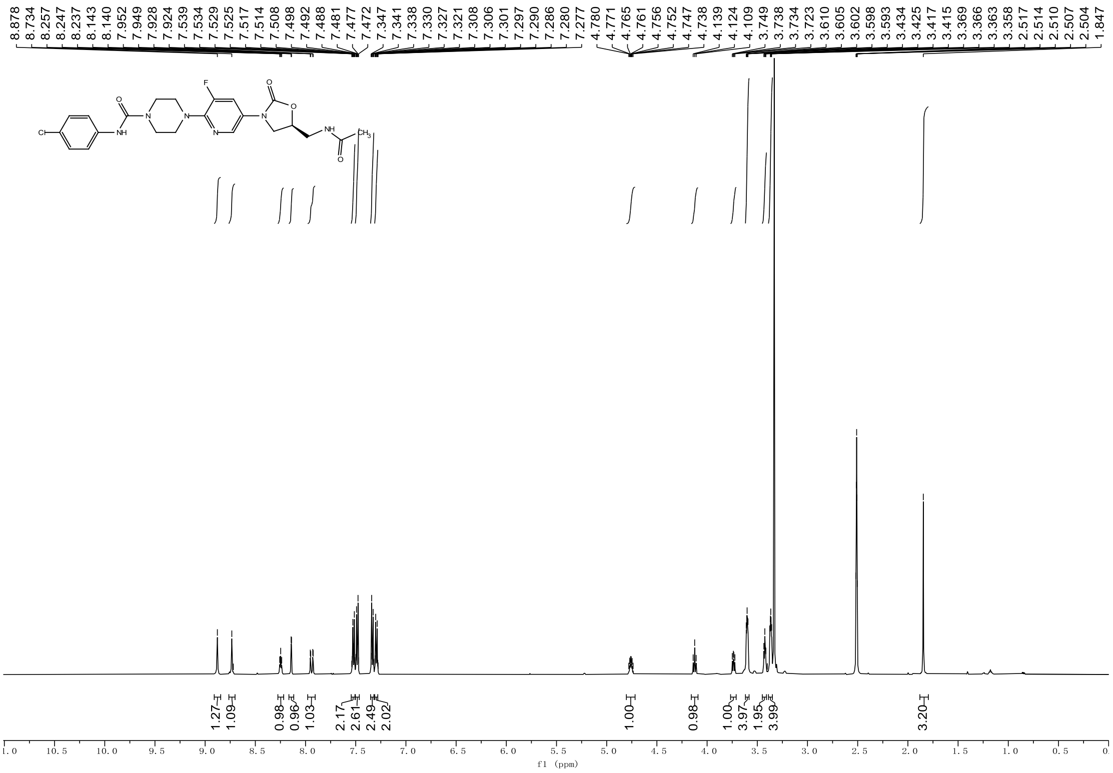

Fig. S111. ^{1}H NMR Spectrum (DMSO-d_6, 400 MHz) of **21f**.

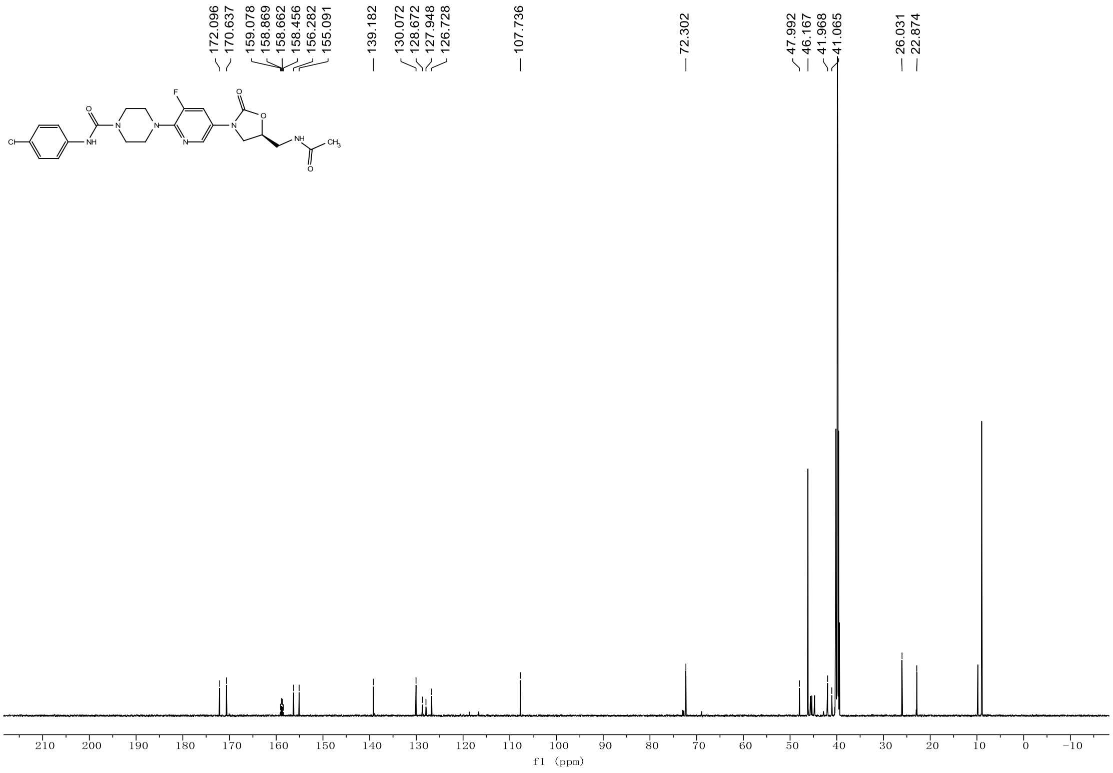

Fig. S112. ^{13}C NMR Spectrum (DMSO-d_6, 75 MHz) of **21f**.

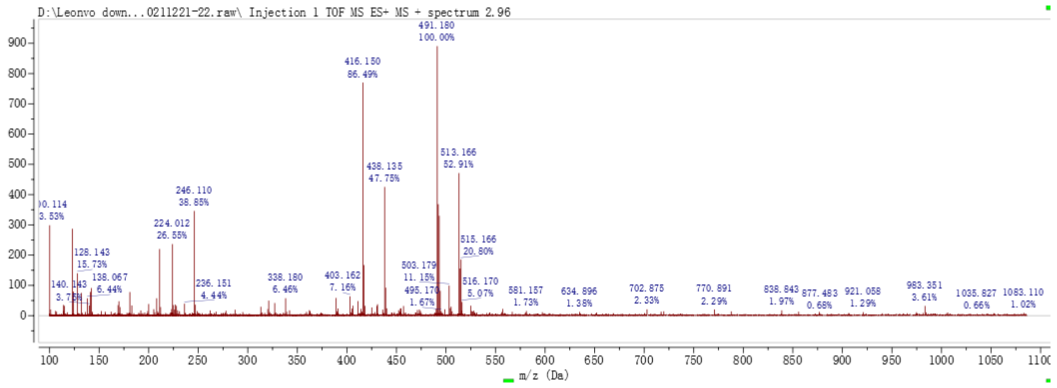

Fig. S113. MS calcd for $C_{22}H_{24}ClFN_6O_4$ (Mwt.: 490.92): m/z 491.180 ([M+H]$^+$, bp) of **21f**.
